# Supplementary material for: One-Pot Synthesis and Molecular Modeling Studies of New Bioactive Spiro-Oxindoles Based on Uracil Derivatives as SARS-CoV-2 Inhibitors Targeting RNA Polymerase and Spike Glycoprotein
Source: Pharmaceuticals (Basel). 2022 Mar 20;15(3):376. doi: 10.3390/ph15030376 (PMC8954694; doi:10.3390/ph15030376)
Supplement: Supplementary file 1 [file pharmaceuticals-15-00376-s001.zip › pharmaceuticals-1604047-supplementary.pdf]

Supplementary material

One-pot synthesis and molecular modeling studies of new bioactive spiro-oxindoles based on uracil derivatives as SARS-CoV-2 inhibitors targeting RNA polymerase and spike glycoprotein

Samar A. El-Kalyoubi[1], Ahmed Ragab[2,*,] Ola A. Abu Ali[3], Yousry A. Ammar[2], Mohamed G. Seadawy[4], Aya Ahmed[5], and Eman A. Fayed[1,*]

[1] Pharmaceutical Organic Chemistry Department, Faculty of Pharmacy (Girls), Al-Azhar University, Cairo 11754, Egypt; s.elkalyoubi@gmail.com; alfayed_e@azhar.edu.eg & alfayed_e@yahoo.com
[2] Chemistry Department, Faculty of Science (Boys), Al-Azhar University, Cairo 11884, Egypt; ahmed_ragab7@ymail.com & ahmed_ragab@azhar.edu.eg; yossry@yahoo.com
[3] Department of Chemistry, College of Science, Taif University, P.O. Box 11099, Taif 21944, Saudi Arabia;
[4] Main Chemical Laboratories, Egypt Army; Biologist202054@yahoo.com
[5] Molecular Virology and Immunology Unit, Cancer Institute, Cairo University, Kasr Al-Ainist., Fom El-Khaleeg, Cairo 11976, Egypt; ayaah2223@yahoo.com

---

✉ Corresponding author: Ahmed Ragab (Ph.D) Tel.: + 202 01009341359; E-mail: ahmed_ragab7@ymail.com & ahmed_ragab@azhar.edu.eg ; Eman A. Fayed (PhD); alfayed_e@azhar.edu.eg & alfayed_e@yahoo.com; and Yousry A. Ammar (PhD); yossry@yahoo.com and yossry@ azhar.edu.eg

---

% of viability for Chloroquine

conc µM/mL	% viability	% viability	% viability		
15	5.597655	6.297361	4.897948	16.79296	5.597655
7.5	4.198241	4.198241	4.198241	12.59472	4.198241
3.75	3.498534	3.498534	4.897948	11.89502	3.965005
1.875	2.798827	4.897948	3.498534	11.19531	3.73177
0.937	3.498534	5.597655	4.897948	13.99414	4.664712
0.468	19.59179	18.89208	23.79003	62.2739	20.75797
0.234	30.08739	33.58593	39.18358	102.8569	34.28563
0.117	81.16599	82.56541	93.76071	257.4921	85.8307
0.0585	100	100	100	300	100
0.029	100	100	100	300	100

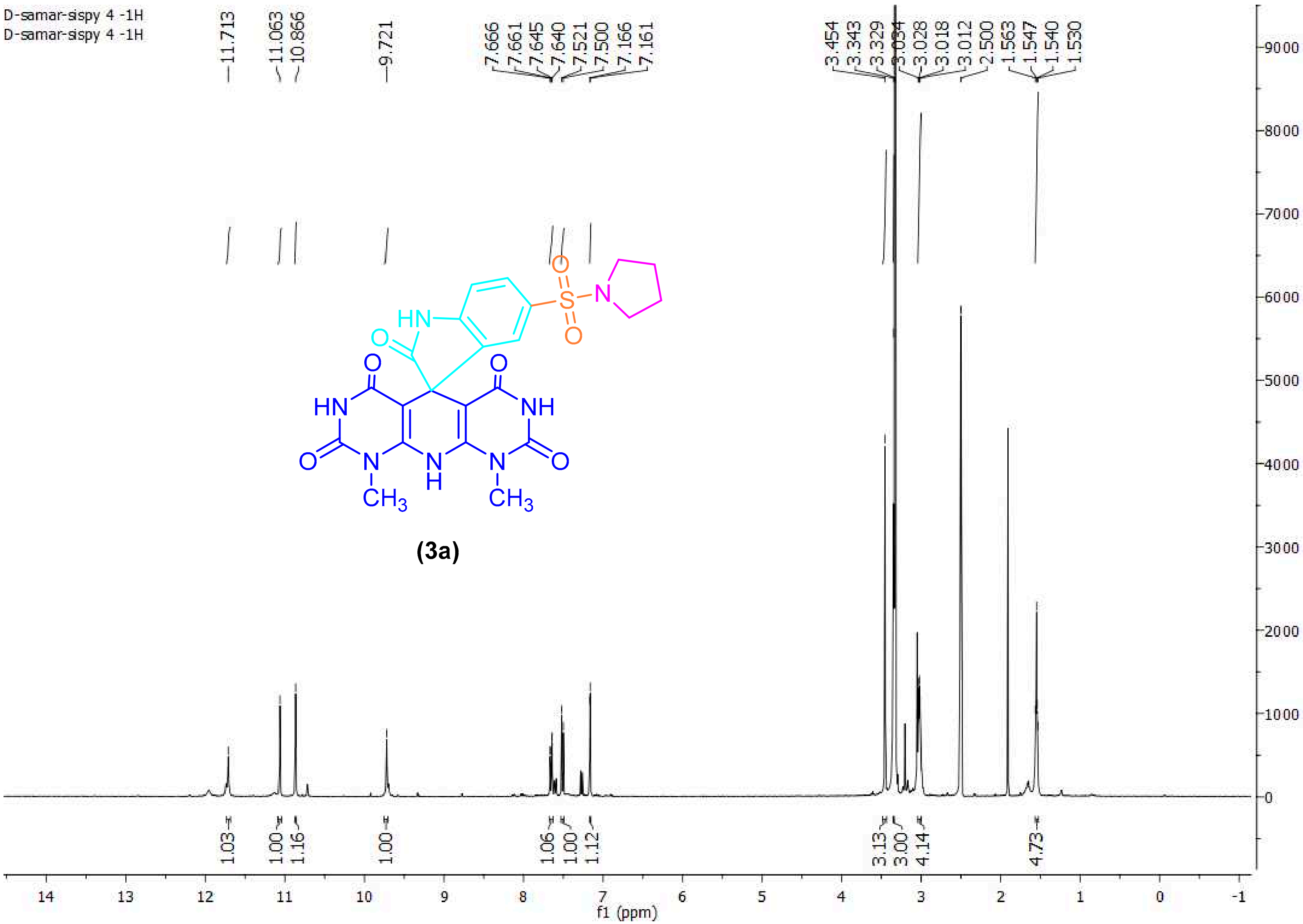
D-samar-sispy 4 -1H
D-samar-sispy 4 -1H
11.713
11.063
10.866
9.721
7.666
7.661
7.645
7.640
7.521
7.500
7.166
7.161
3.454
3.343
3.329
3.028
3.018
3.012
2.500
1.563
1.547
1.540
1.530
1.03
1.00
1.16
1.00
1.06
1.00
1.12
3.13
3.00
4.14
4.73
f1 (ppm)
HN
O
S
N
NH
H
CH3

(3a)

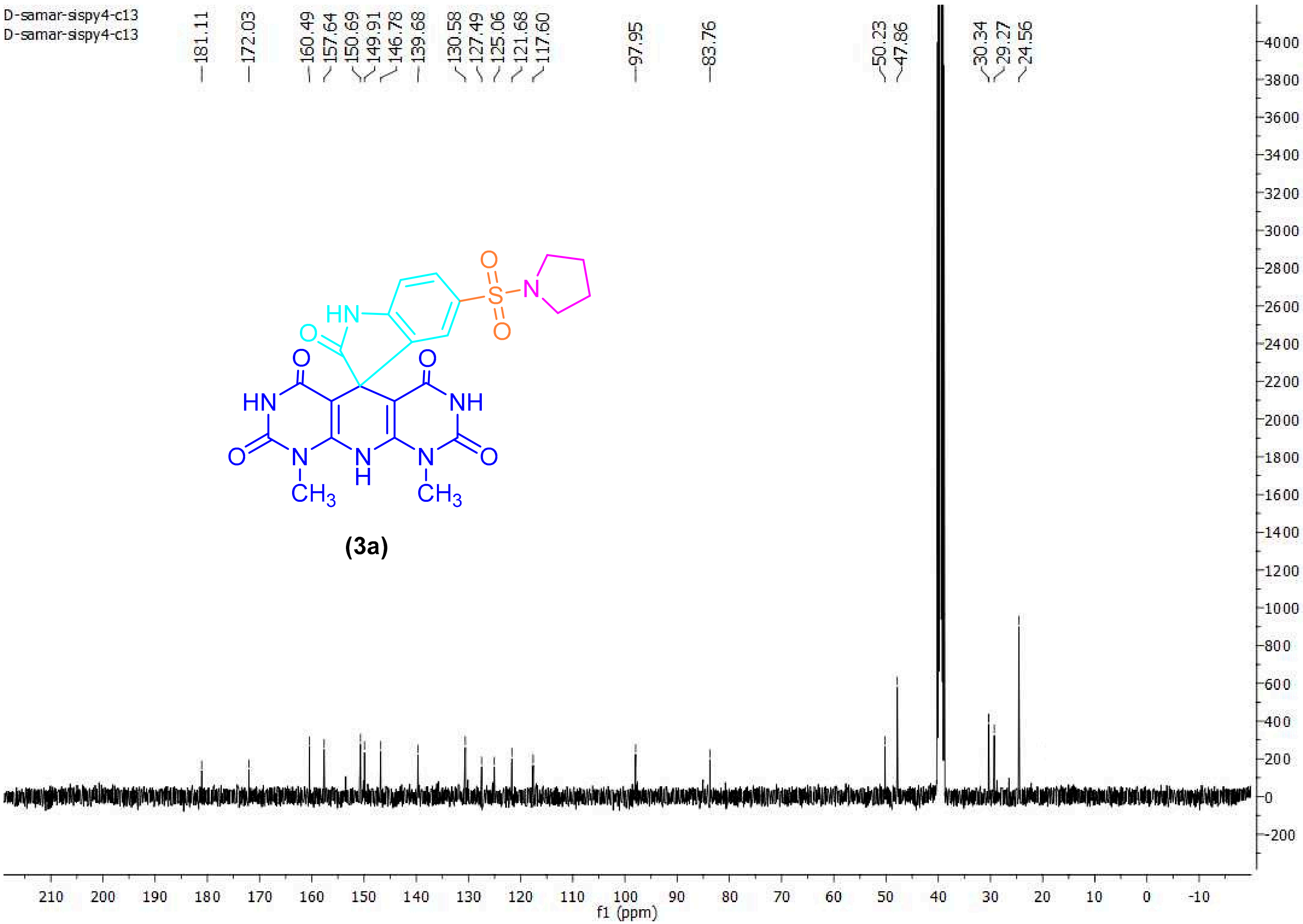
D-samar-sispy4-c13
D-samar-sispy4-c13
181.11
172.03
160.49
157.64
150.69
149.91
146.78
139.68
130.58
127.49
125.06
121.68
117.60
97.95
83.76
50.23
47.86
30.34
29.27
24.56
(3a)
f1 (ppm)

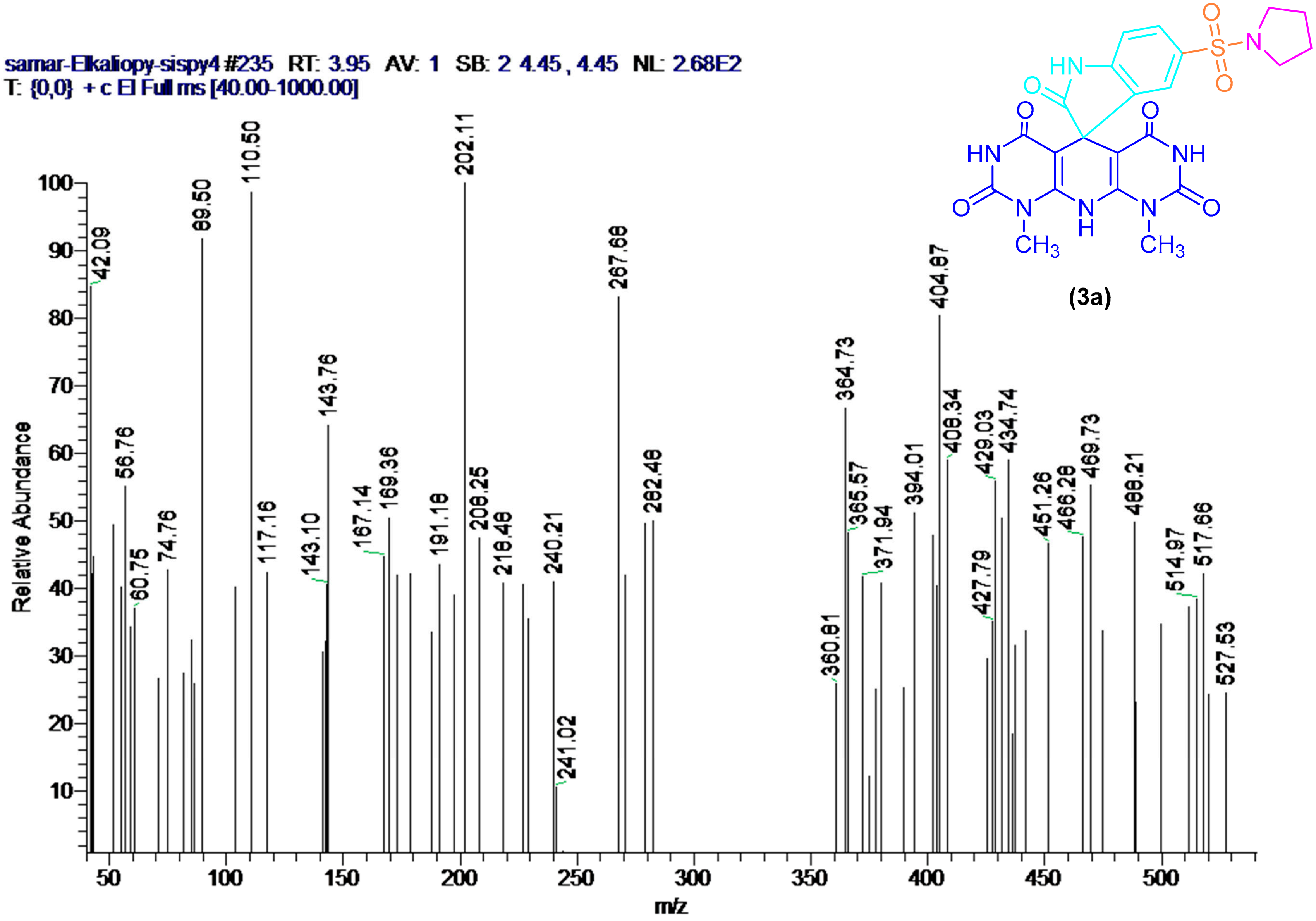
samar-Elkaliopy-sispy4 #235 RT: 3.95 AV: 1 SB: 2 4.45 , 4.45 NL: 2.68E2
T: {0,0} + c EI Full ms [40.00-1000.00]
Relative Abundance
m/z
42.09
56.76
60.75
74.76
89.50
110.50
117.16
143.10
143.76
167.14
169.36
191.18
202.11
208.25
218.48
240.21
241.02
267.68
282.48
360.81
364.73
365.57
371.94
394.01
404.87
406.34
427.79
429.03
434.74
451.26
466.28
469.73
488.21
514.97
517.66
527.53
HN
O
O
S
O
N
O
O
HN
NH
O
N
N
H
N
O
CH3
CH3
(3a)

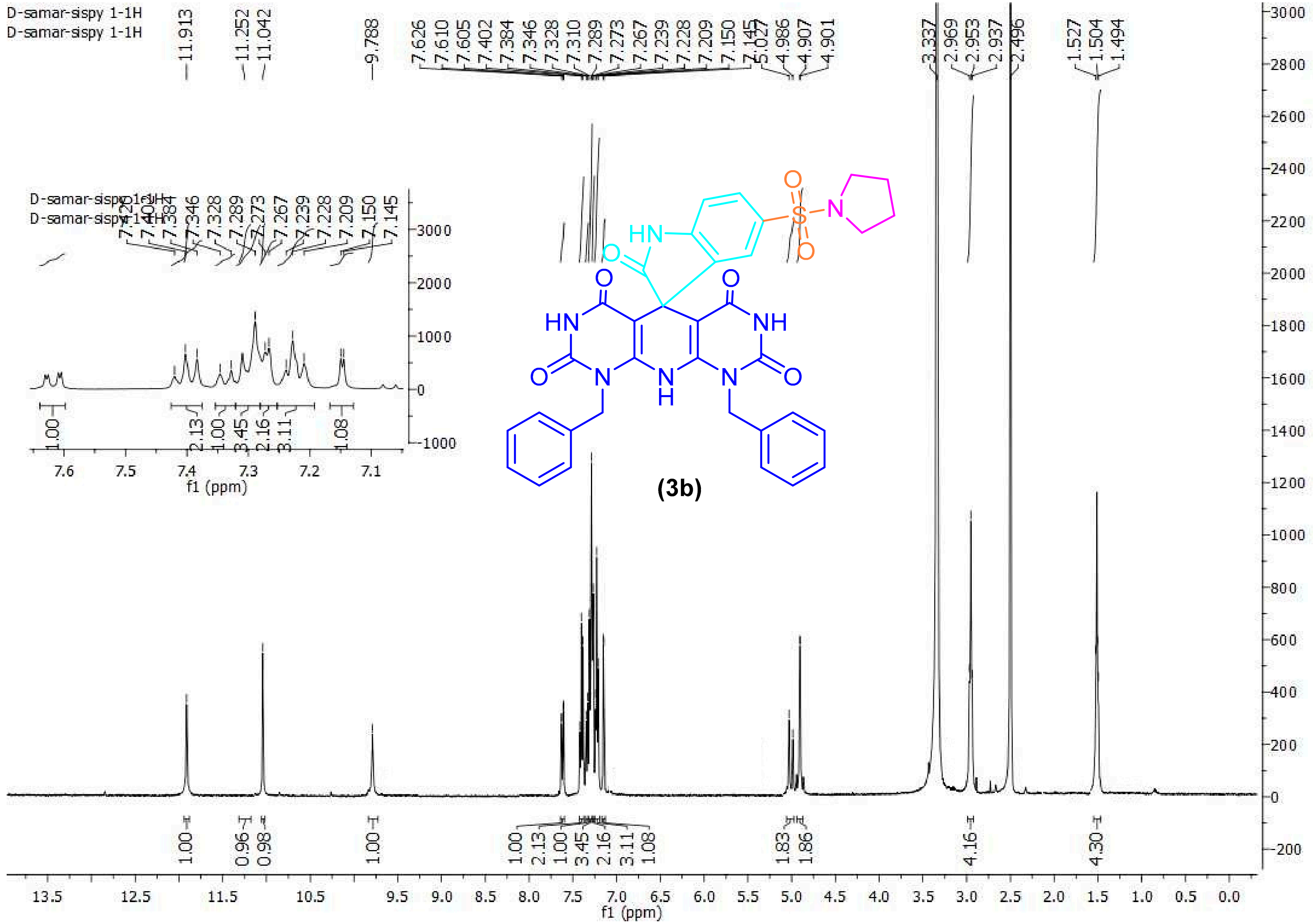
D-samar-sispy 1-1H
D-samar-sispy 1-1H
11.913
11.252
11.042
9.788
7.626
7.610
7.605
7.402
7.384
7.346
7.328
7.310
7.289
7.273
7.267
7.239
7.228
7.209
7.150
7.145
5.027
4.986
4.907
4.901
3.337
2.969
2.953
2.937
2.496
1.527
1.504
1.494
1.00
0.96
0.98
1.00
1.00
2.13
1.00
3.45
2.16
3.11
1.08
1.83
1.86
4.16
4.30
f1 (ppm)
HN
O
S
N
NH
H
(3b)

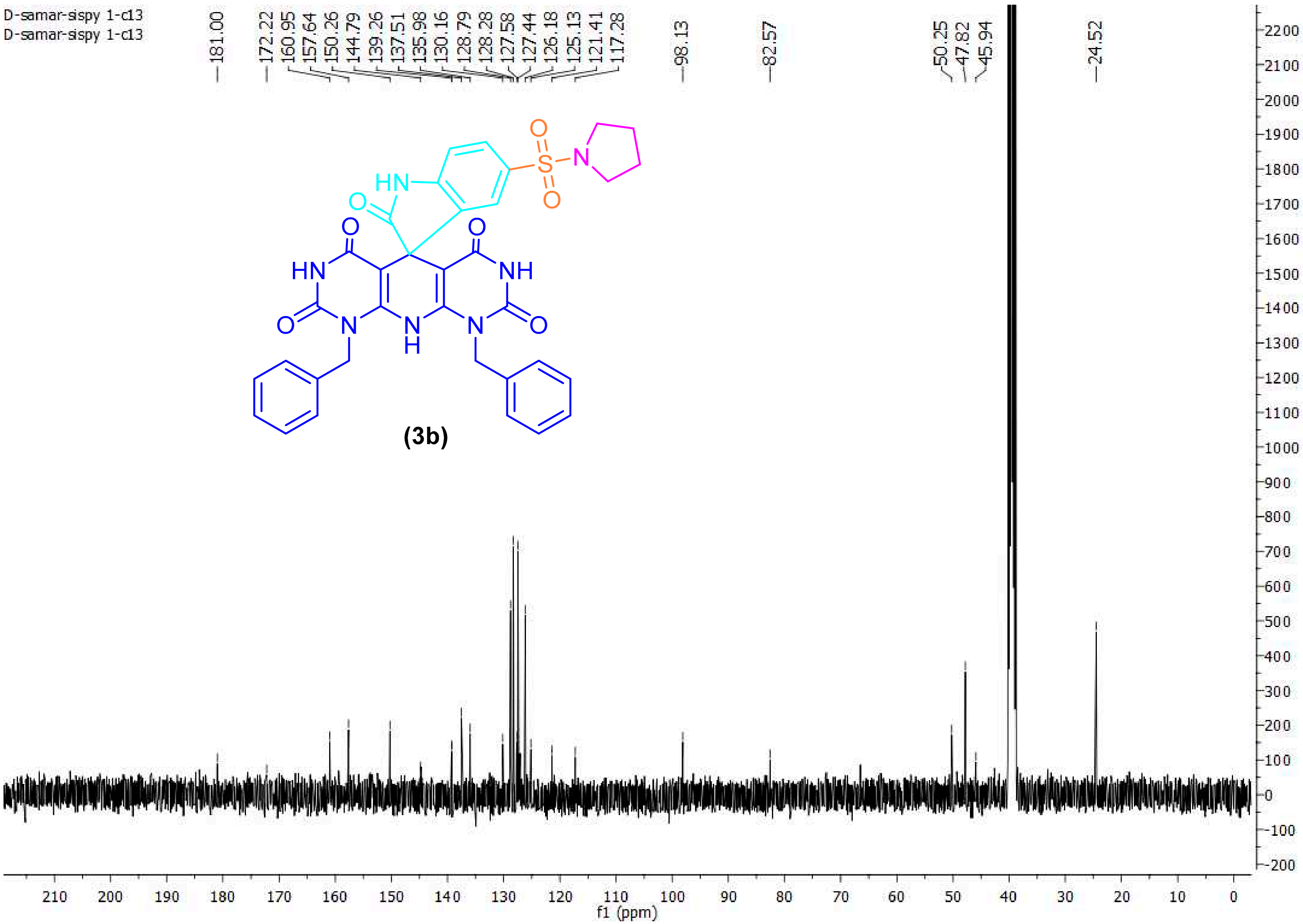

D-samar-sispy 1-c13
D-samar-sispy 1-c13
181.00
172.22
160.95
157.64
150.26
144.79
139.26
137.51
135.98
130.16
128.79
128.28
127.58
127.44
126.18
125.13
121.41
117.28
98.13
82.57
50.25
47.82
45.94
24.52
(3b)
f1 (ppm)

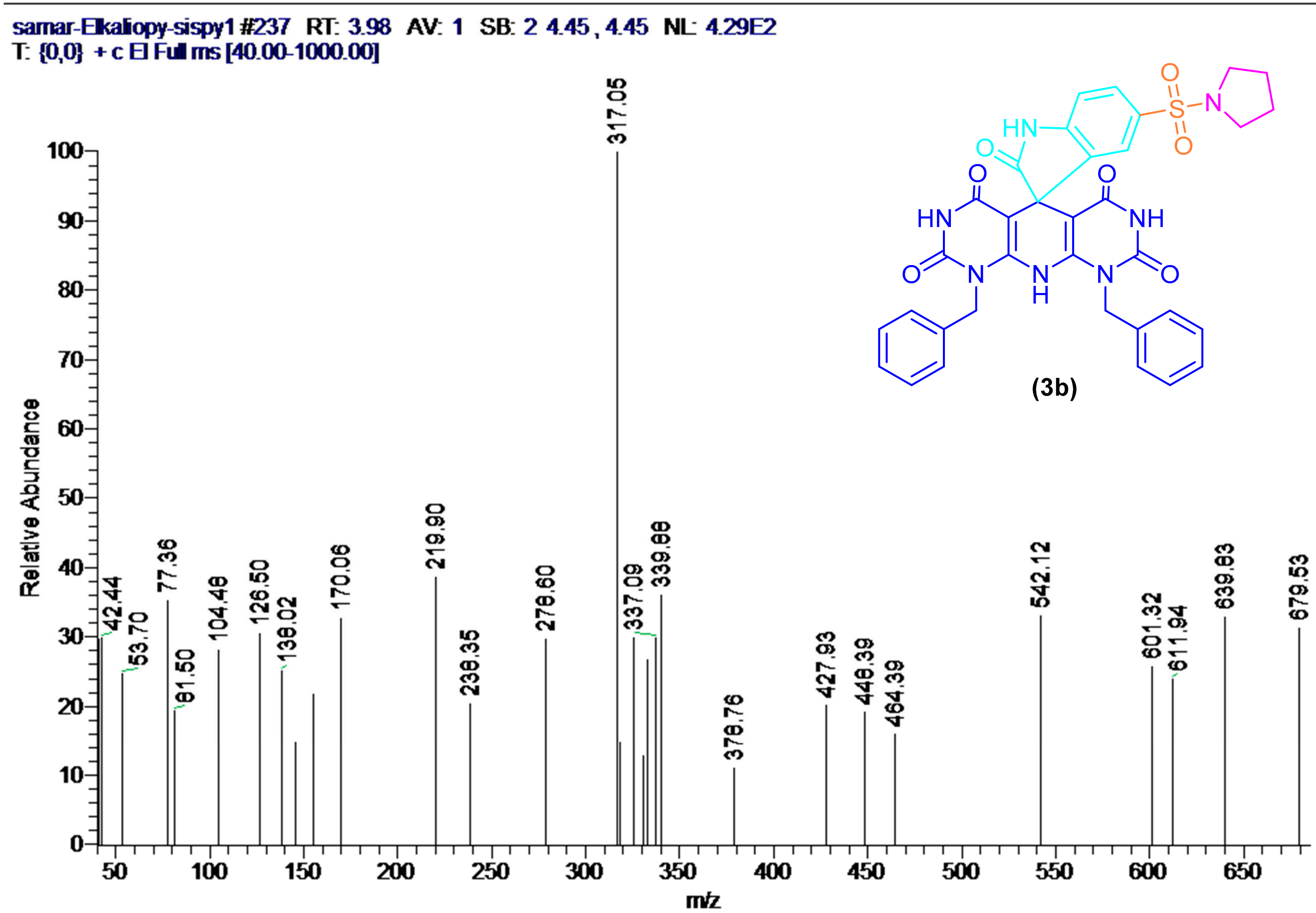
samar-Elkaliopy-sispy1 #237 RT: 3.98 AV: 1 SB: 2 4.45 , 4.45 NL: 4.29E2
T: {0,0} + c EI Full ms [40.00-1000.00]
Relative Abundance
m/z
317.05
42.44
53.70
77.36
81.50
104.48
126.50
138.02
170.06
219.90
238.35
278.60
337.09
339.88
378.76
427.93
448.39
464.39
542.12
601.32
611.94
639.83
679.53
HN
O
O
S
N
NH
N
H
(3b)

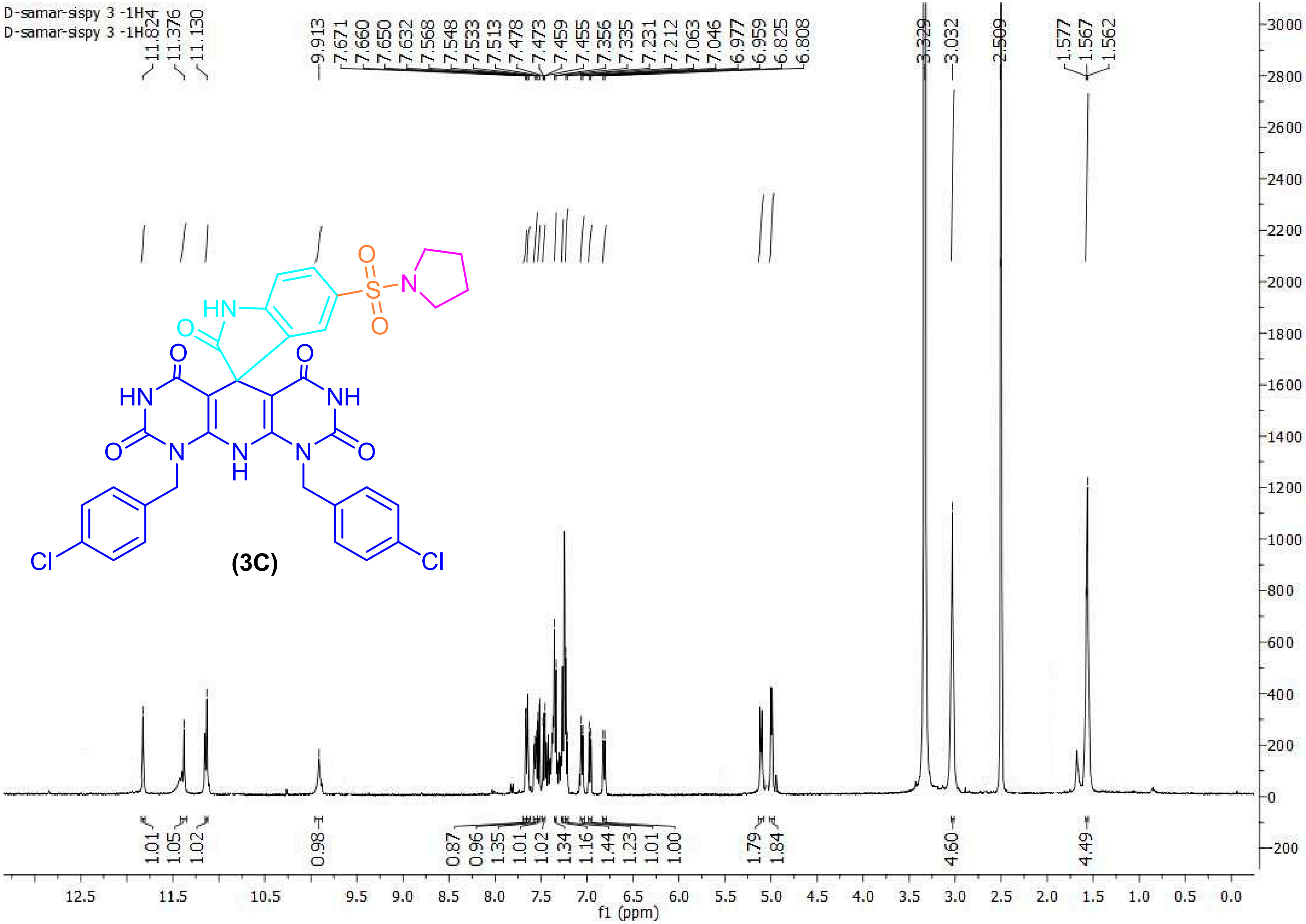
D-samar-sispy 3 -1H
D-samar-sispy 3 -1H
11.824
11.376
11.130
9.913
7.671
7.660
7.650
7.632
7.568
7.548
7.533
7.513
7.478
7.473
7.459
7.455
7.356
7.335
7.231
7.212
7.063
7.046
6.977
6.959
6.825
6.808
3.329
3.032
2.509
1.577
1.567
1.562
(3C)
1.01
1.05
1.02
0.98
0.87
0.96
1.35
1.01
1.02
1.34
1.16
1.44
1.23
1.01
1.00
1.79
1.84
4.60
4.49
f1 (ppm)

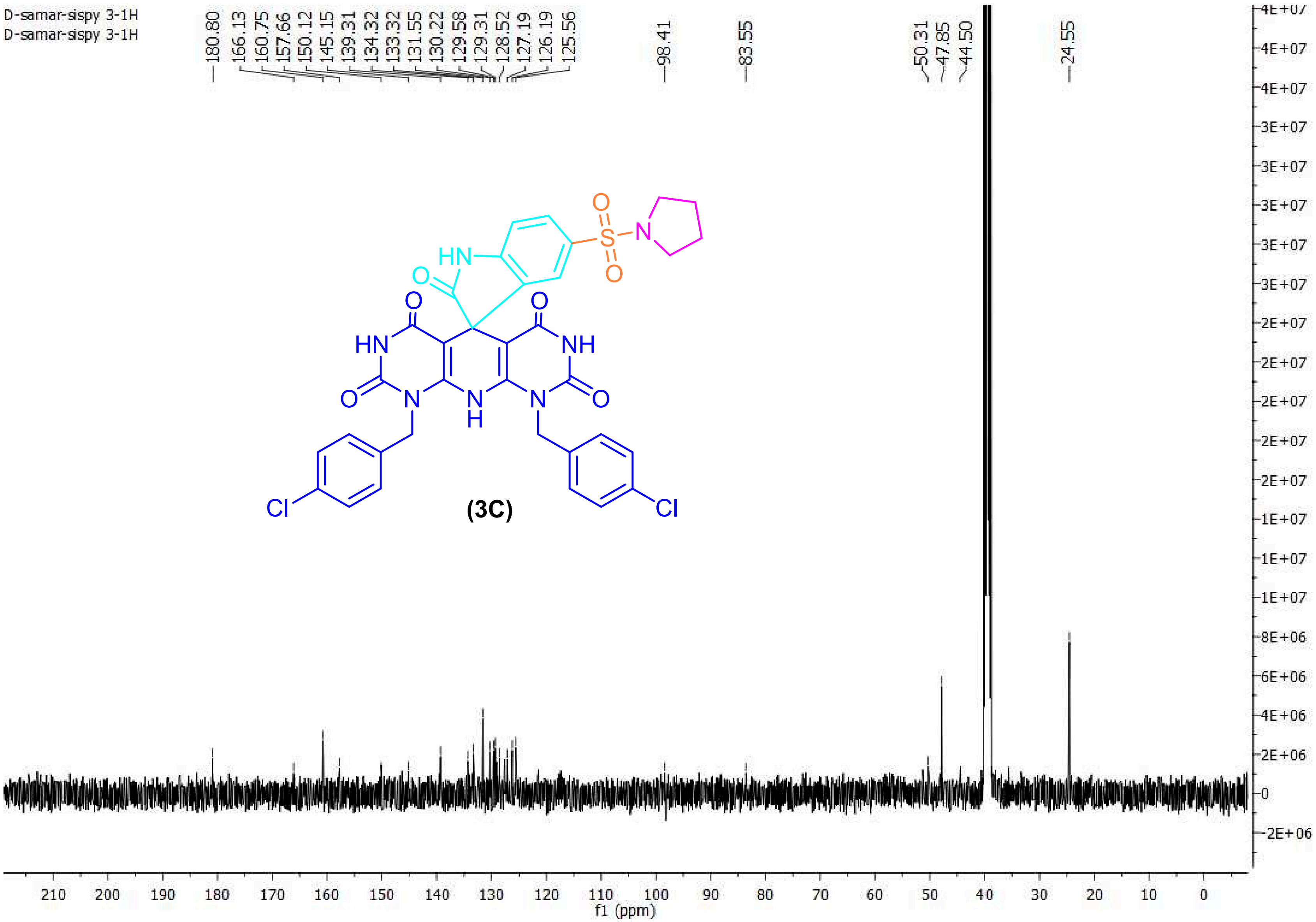
D-samar-sispy 3-1H
D-samar-sispy 3-1H
180.80
166.13
160.75
157.66
150.12
145.15
139.31
134.32
133.32
131.55
130.22
129.58
129.31
128.52
127.19
126.19
125.56
98.41
83.55
50.31
47.85
44.50
24.55
HN
O
S
N
HN
NH
N
N
H
Cl
(3C)
f1 (ppm)

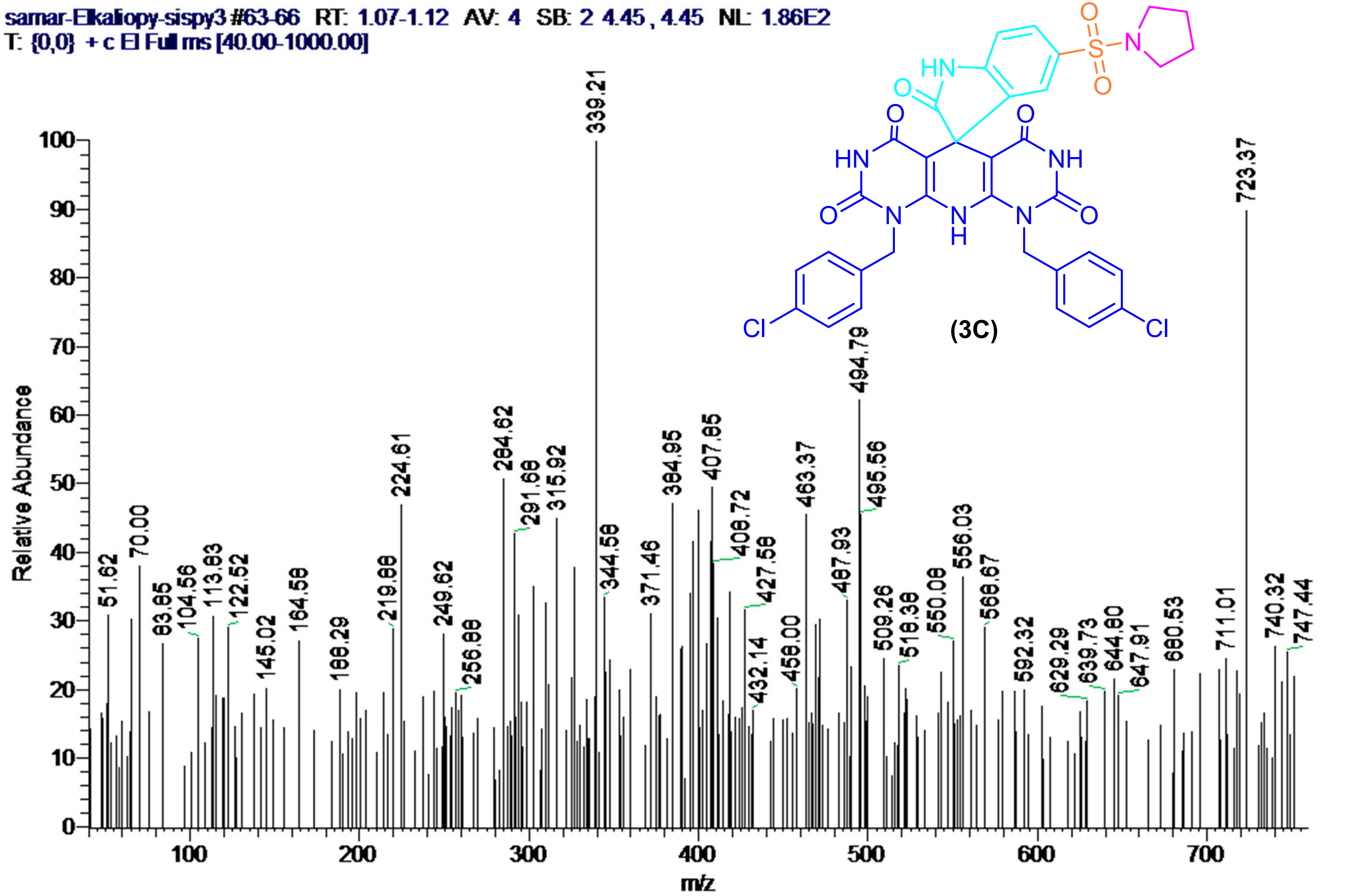
samar-Elkaliopy-sispy3 #63-66 RT: 1.07-1.12 AV: 4 SB: 2 4.45 , 4.45 NL: 1.86E2
T: {0,0} + c EI Full ms [40.00-1000.00]
Relative Abundance
m/z
(3C)
HN
O
S
N
NH
N
H
Cl
339.21
723.37
494.79
284.62
224.61
407.85
384.95
463.37
495.56
315.92
291.68
408.72
70.00
427.58
556.03
487.93
344.58
51.62
113.83
122.52
104.56
83.85
164.58
371.46
568.67
550.08
509.26
518.38
249.62
219.88
740.32
747.44
711.01
680.53
145.02
188.29
256.88
432.14
458.00
592.32
629.29
639.73
644.80
647.91

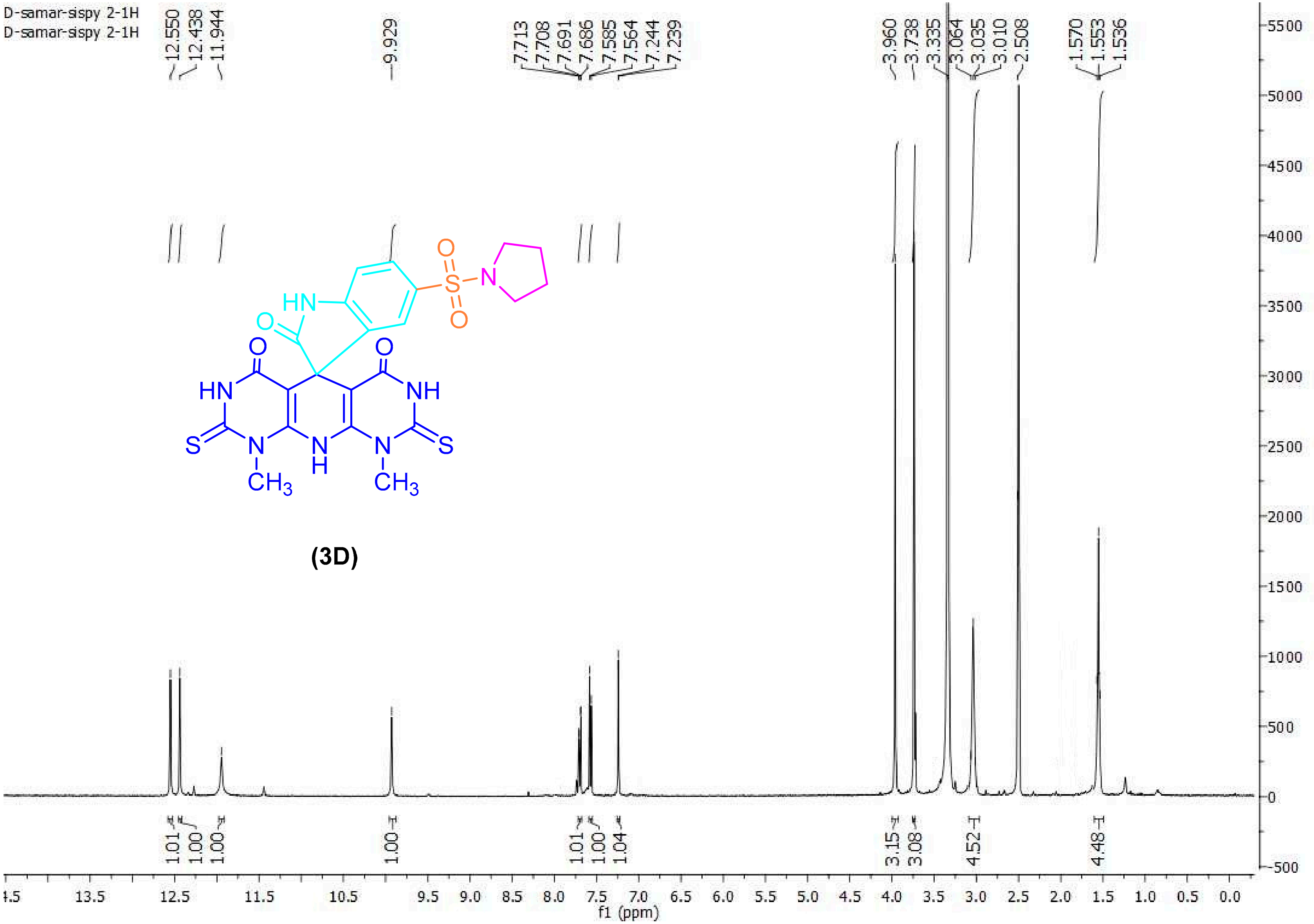
D-samar-sispy 2-1H
D-samar-sispy 2-1H
12.550
12.438
11.944
9.929
7.713
7.708
7.691
7.686
7.585
7.564
7.244
7.239
3.960
3.738
3.335
3.064
3.035
3.010
2.508
1.570
1.553
1.536
HN
O
O
S
N
O
O
HN
NH
S
N
N
H
N
S
CH3
CH3
(3D)
1.01
1.00
1.00
1.00
1.01
1.00
1.04
3.15
3.08
4.52
4.48
f1 (ppm)

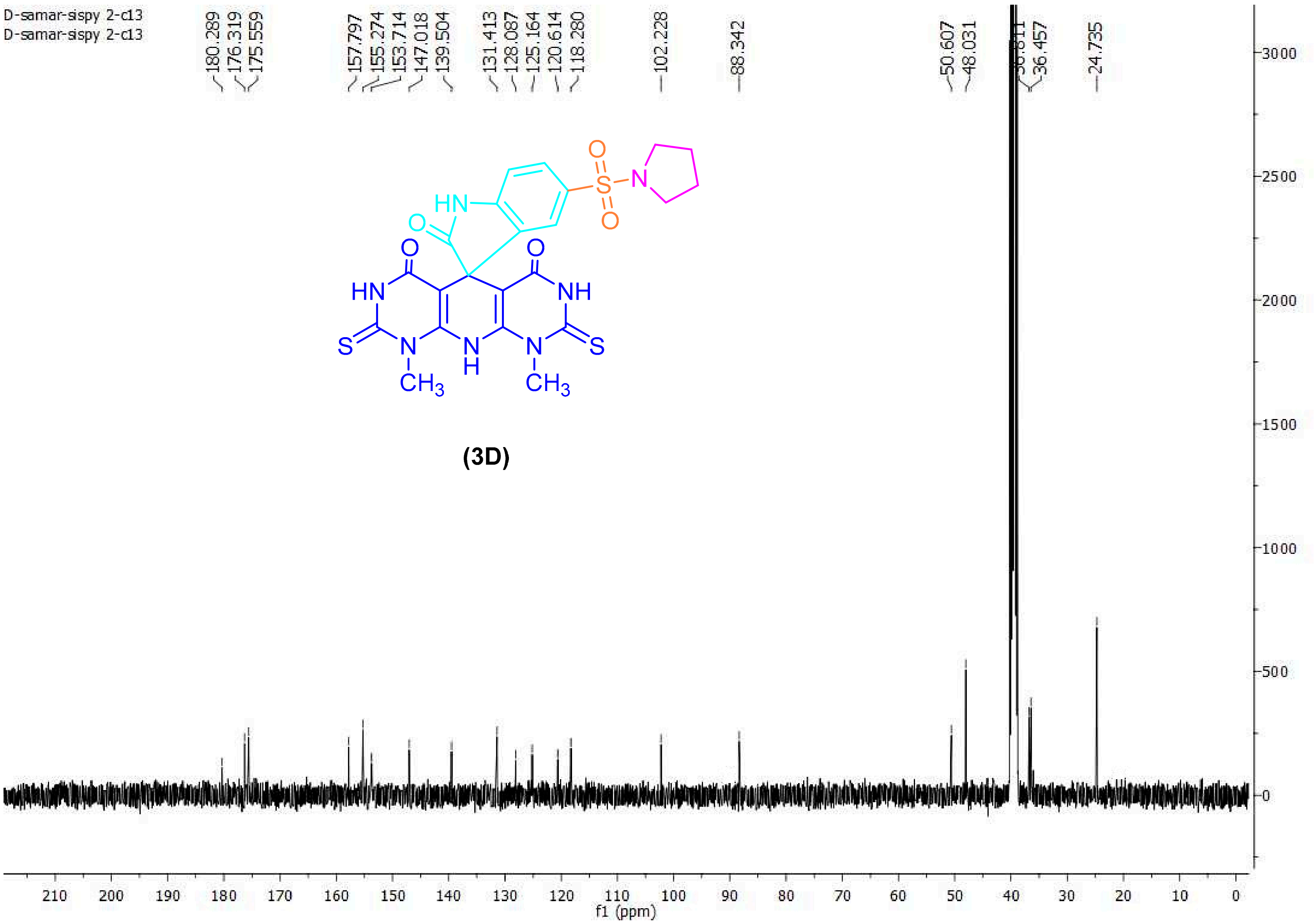
D-samar-sispy 2-c13
D-samar-sispy 2-c13
180.289
176.319
175.559
157.797
155.274
153.714
147.018
139.504
131.413
128.087
125.164
120.614
118.280
102.228
88.342
50.607
48.031
36.457
24.735
f1 (ppm)

(3D)

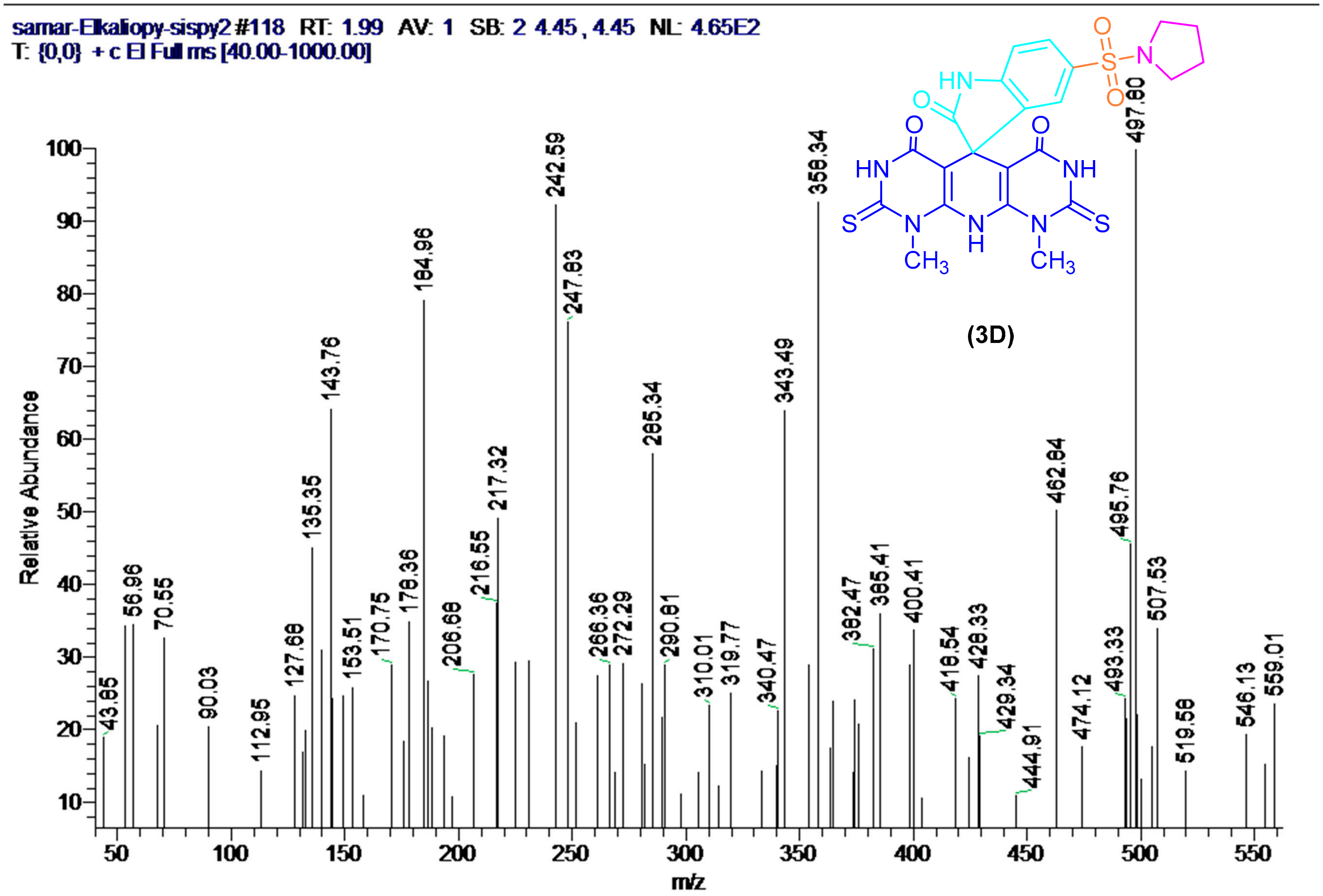
samar-Elkaliopy-sispy2 #118 RT: 1.99 AV: 1 SB: 2 4.45 , 4.45 NL: 4.65E2
T: {0,0} + c EI Full ms [40.00-1000.00]
Relative Abundance
m/z
43.65
56.96
70.55
90.03
112.95
127.68
135.35
143.76
153.51
170.75
178.36
184.96
206.68
216.55
217.32
242.59
247.83
266.36
272.29
285.34
290.61
310.01
319.77
340.47
343.49
358.34
382.47
385.41
400.41
418.54
428.33
429.34
444.91
462.84
474.12
493.33
495.76
497.80
507.53
519.58
546.13
559.01
HN
O
S
O
O
N
O
O
HN
NH
S
N
N
H
N
S
CH3
CH3

(3D)

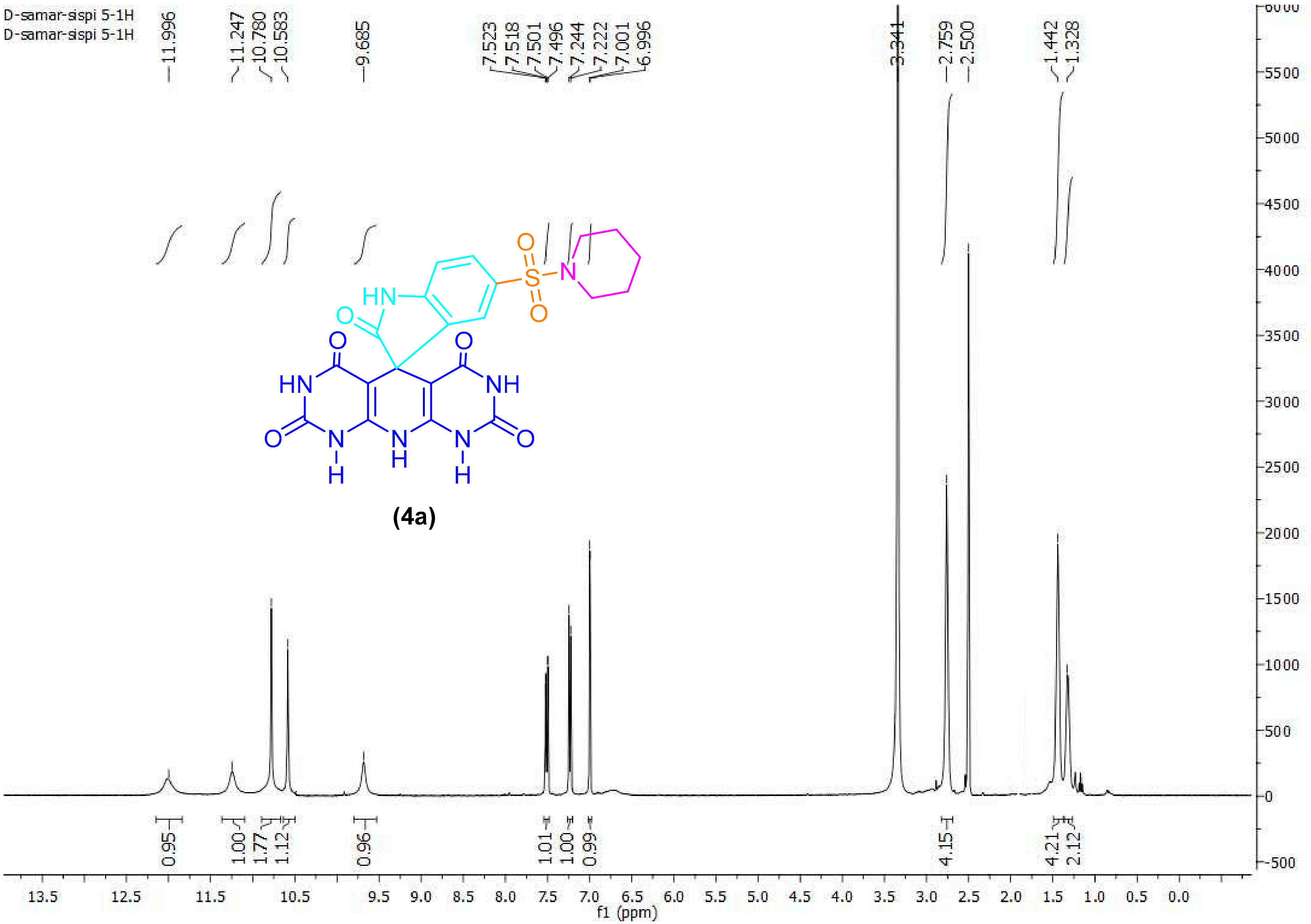
D-samar-sispi 5-1H
D-samar-sispi 5-1H
11.996
11.247
10.780
10.583
9.685
7.523
7.518
7.501
7.496
7.244
7.222
7.001
6.996
3.341
2.759
2.500
1.442
1.328
0.95
1.00
1.77
1.12
0.96
1.01
1.00
0.99
4.15
4.21
2.12
HN
O
S
N
NH
H
(4a)
f1 (ppm)

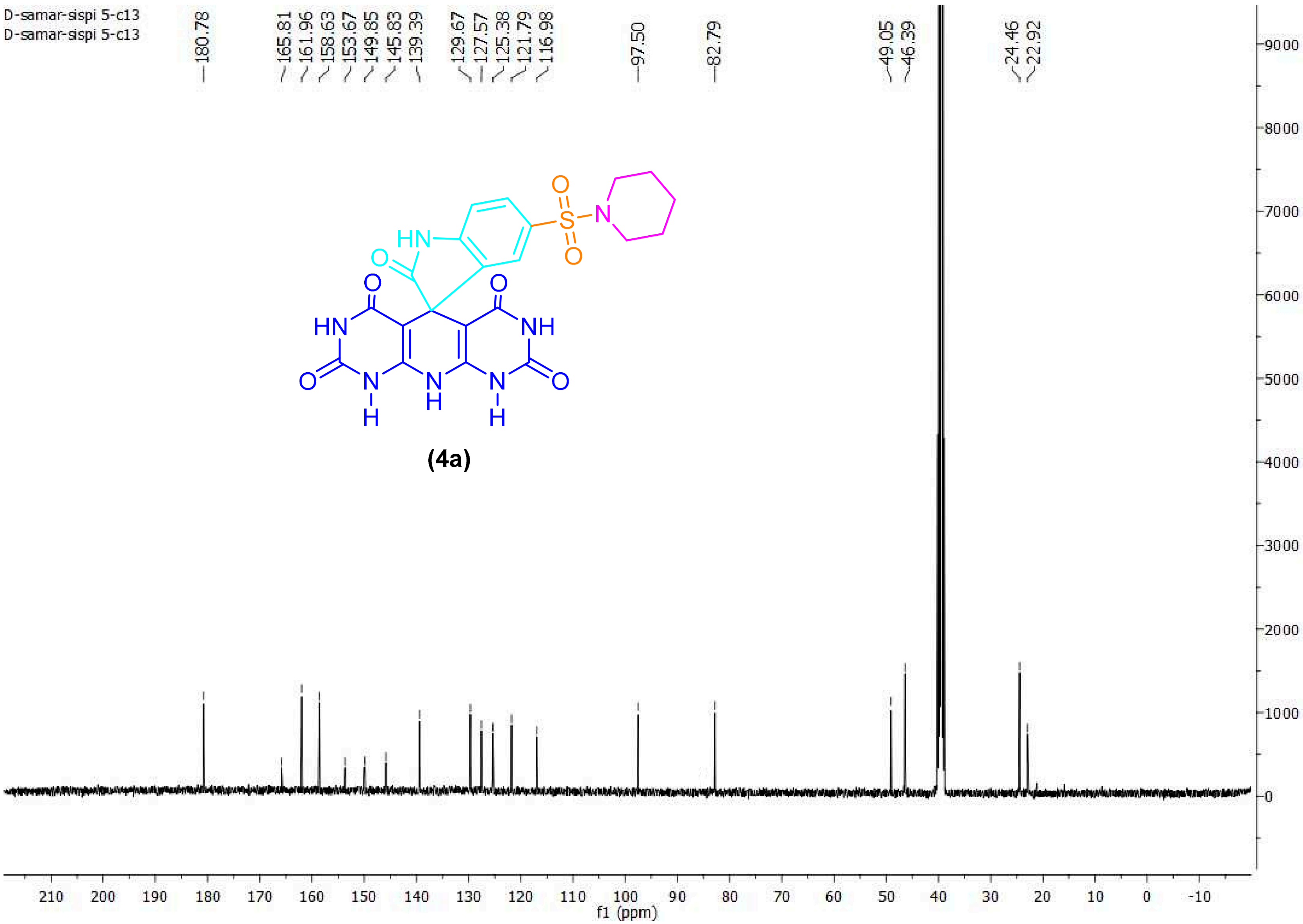
D-samar-sispi 5-c13
D-samar-sispi 5-c13
180.78
165.81
161.96
158.63
153.67
149.85
145.83
139.39
129.67
127.57
125.38
121.79
116.98
97.50
82.79
49.05
46.39
24.46
22.92
(4a)
f1 (ppm)

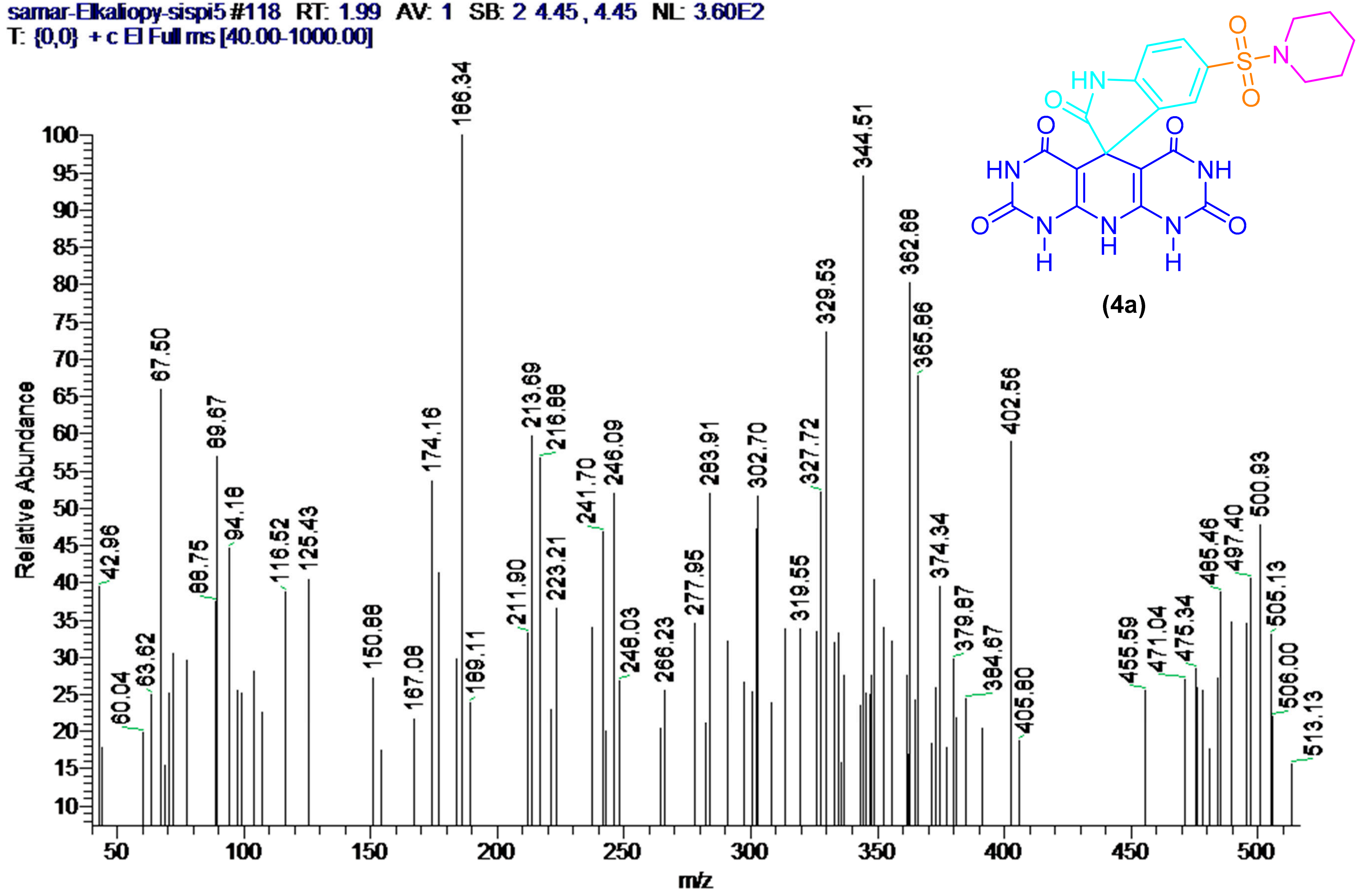

samar-Elkaliopy-sispi5 #118 RT: 1.99 AV: 1 SB: 2 4.45 , 4.45 NL: 3.60E2
T: {0,0} + c EI Full ms [40.00-1000.00]
Relative Abundance
m/z
186.34
344.51
362.68
329.53
365.86
67.50
213.69
216.88
402.56
89.67
174.16
246.09
283.91
302.70
327.72
500.93
241.70
94.16
42.96
116.52
125.43
88.75
374.34
485.46
497.40
505.13
223.21
277.95
319.55
211.90
150.88
63.62
189.11
249.03
266.23
379.87
384.67
455.59
471.04
475.34
167.08
60.04
405.80
506.00
513.13
(4a)

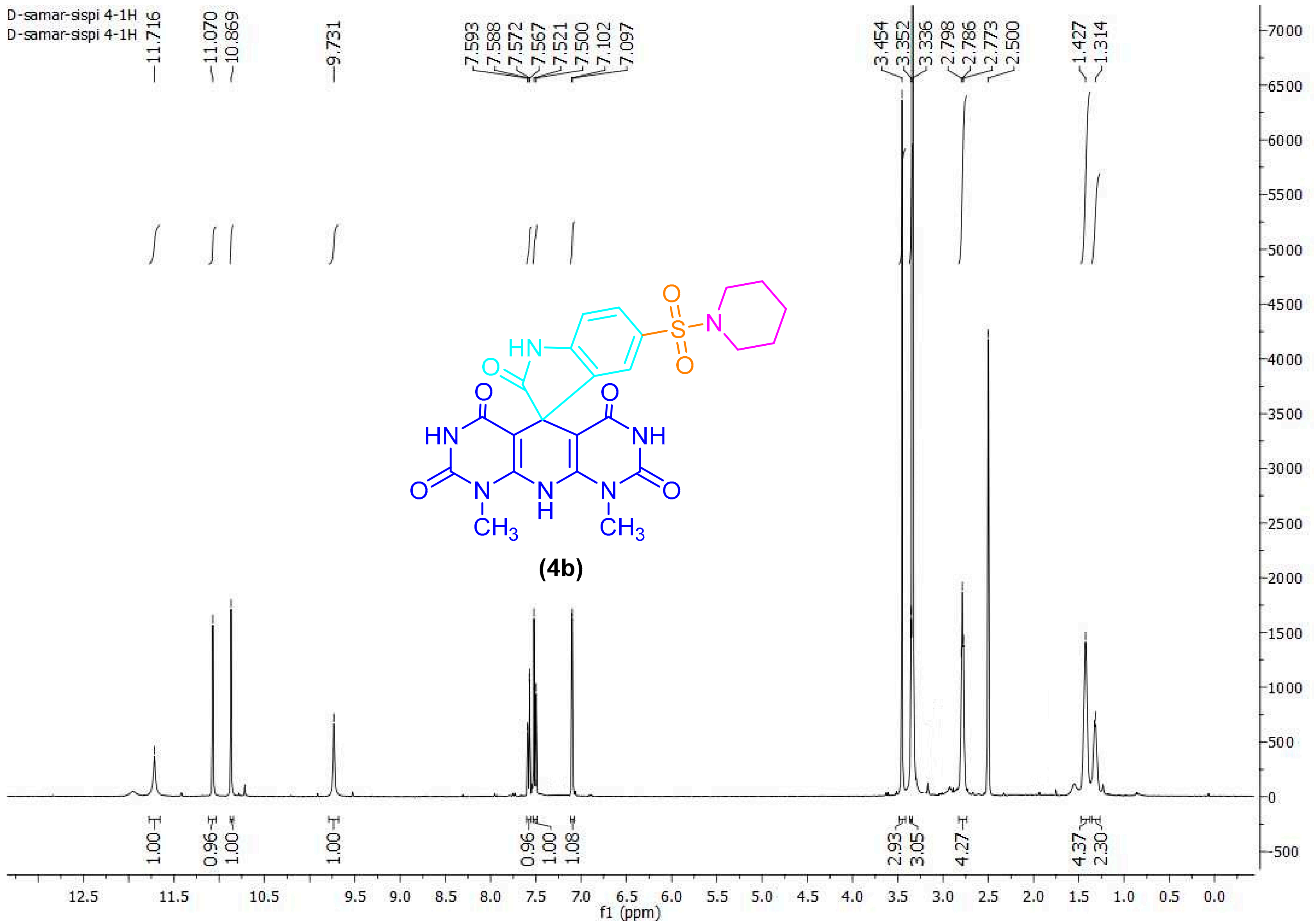
D-samar-sispi 4-1H
D-samar-sispi 4-1H
11.716
11.070
10.869
9.731
7.593
7.588
7.572
7.567
7.521
7.500
7.102
7.097
3.454
3.352
3.336
2.798
2.786
2.773
2.500
1.427
1.314
(4b)
1.00
0.96
1.00
1.00
0.96
1.00
1.08
2.93
3.05
4.27
4.37
2.30
f1 (ppm)

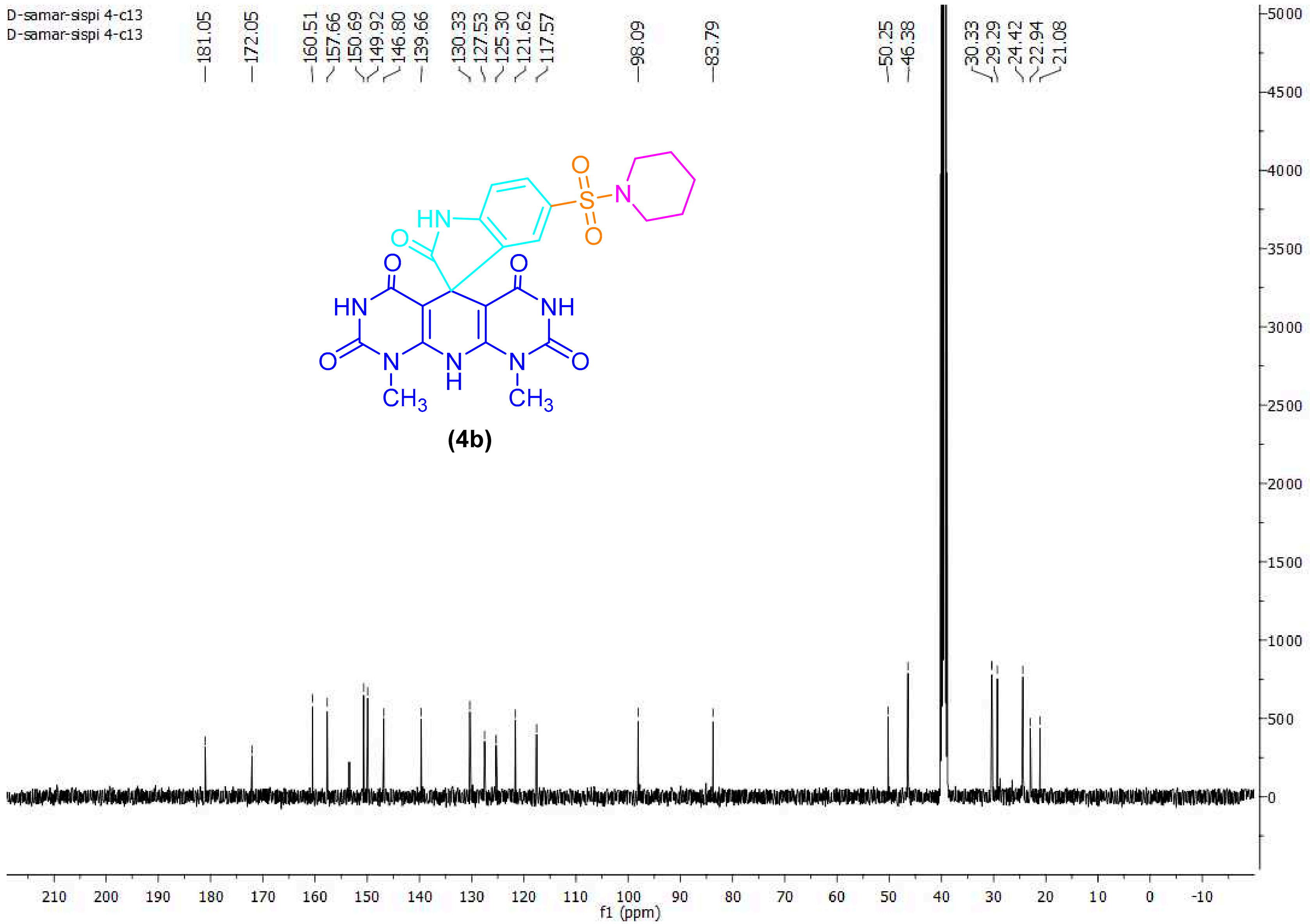
D-samar-sispi 4-c13
D-samar-sispi 4-c13
181.05
172.05
160.51
157.66
150.69
149.92
146.80
139.66
130.33
127.53
125.30
121.62
117.57
98.09
83.79
50.25
46.38
30.33
29.29
24.42
22.94
21.08
HN
O
S
N
NH
H
CH3
(4b)
f1 (ppm)

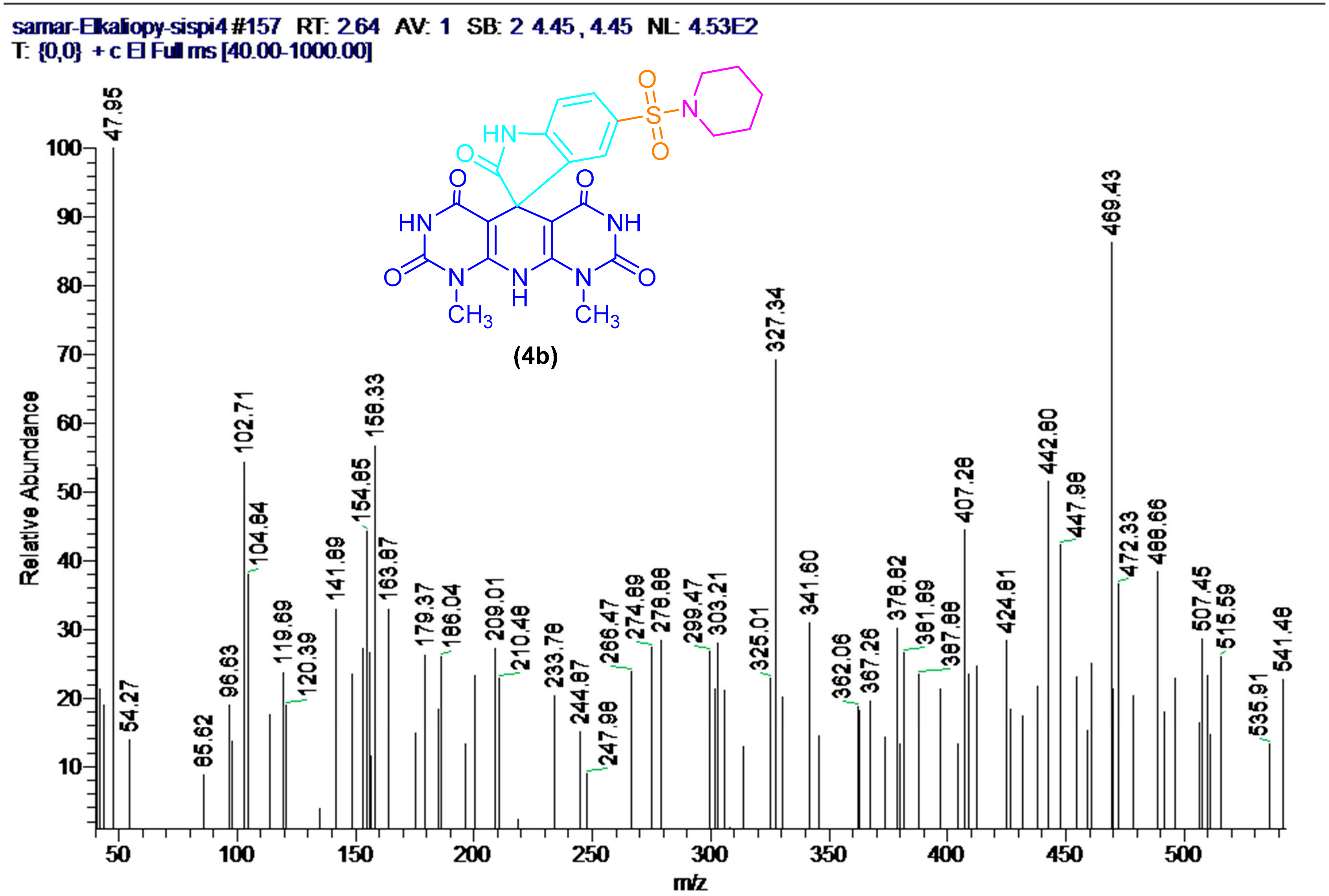
samar-Elkaliopy-sispi4 #157 RT: 2.64 AV: 1 SB: 2 4.45 , 4.45 NL: 4.53E2
T: {0,0} + c EI Full ms [40.00-1000.00]
Relative Abundance
m/z
(4b)
HN
O
HN
NH
N
H
CH3
S
47.95
54.27
85.62
96.63
102.71
104.84
119.69
120.39
141.89
154.85
156.33
163.87
179.37
186.04
209.01
210.48
233.76
244.87
247.98
266.47
274.89
278.88
299.47
303.21
325.01
327.34
341.60
362.06
367.26
378.82
381.89
387.88
407.28
424.81
442.80
447.98
469.43
472.33
488.66
507.45
515.59
535.91
541.48

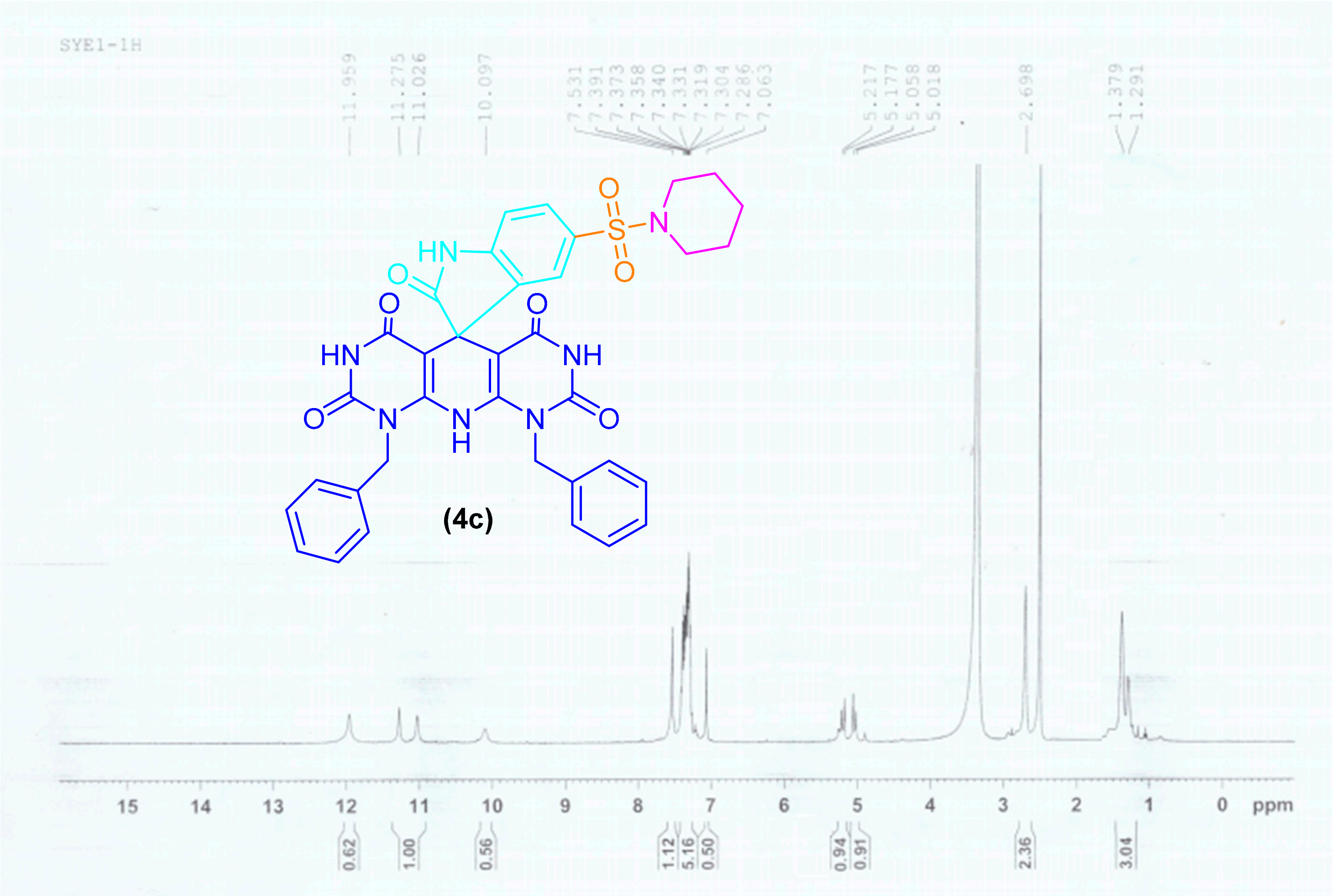
SYE1-1H
11.959
11.275
11.026
10.097
7.531
7.391
7.373
7.358
7.340
7.331
7.319
7.304
7.286
7.063
5.217
5.177
5.058
5.018
2.698
1.379
1.291
(4c)
HN
O
S
N
NH
H
15 14 13 12 11 10 9 8 7 6 5 4 3 2 1 0 ppm
0.62
1.00
0.56
1.12
5.16
0.50
0.94
0.91
2.36
3.04

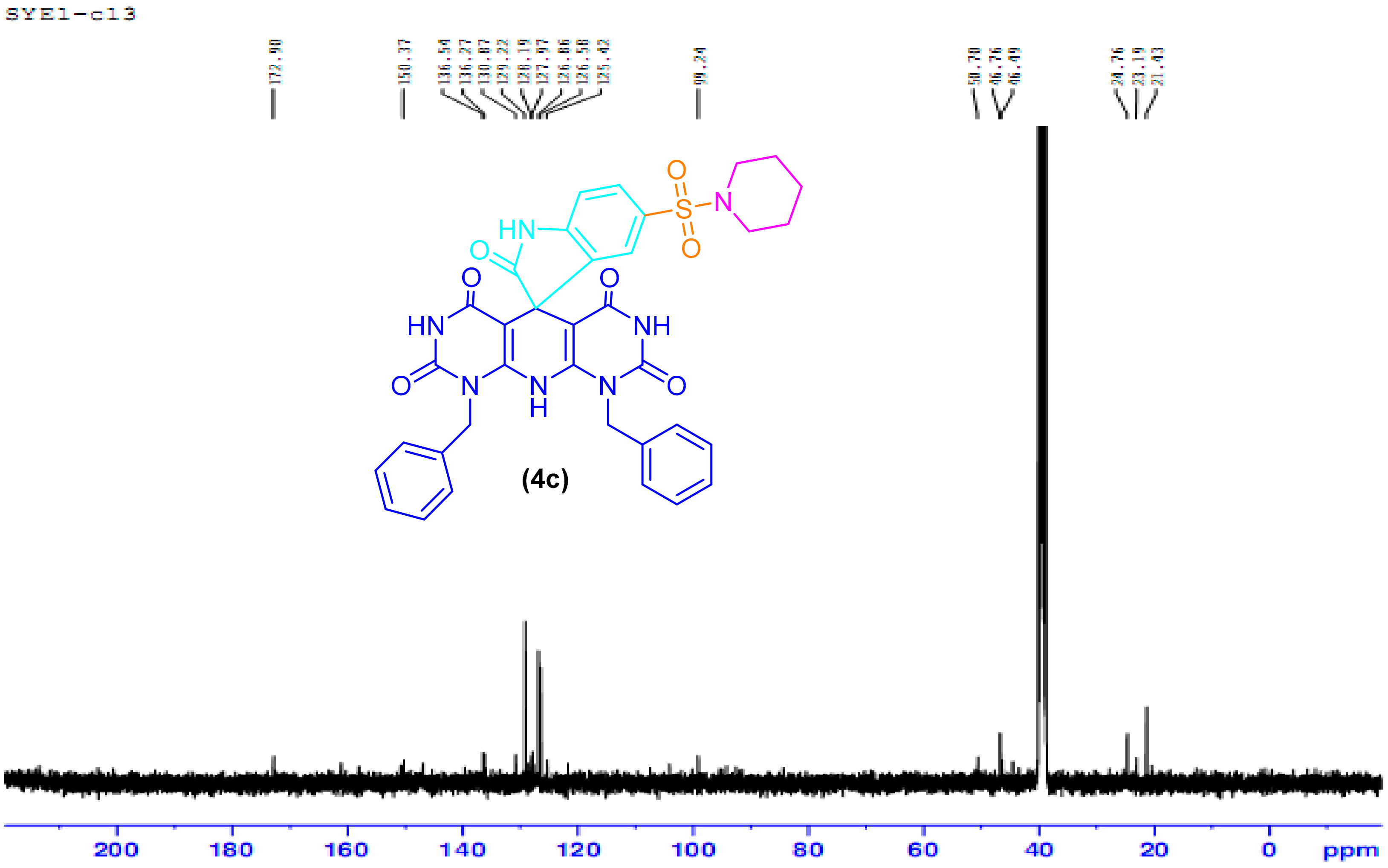
SYE1-c13
HN
O
S
N
NH
H
(4c)
200
180
160
140
120
100
80
60
40
20
0
ppm

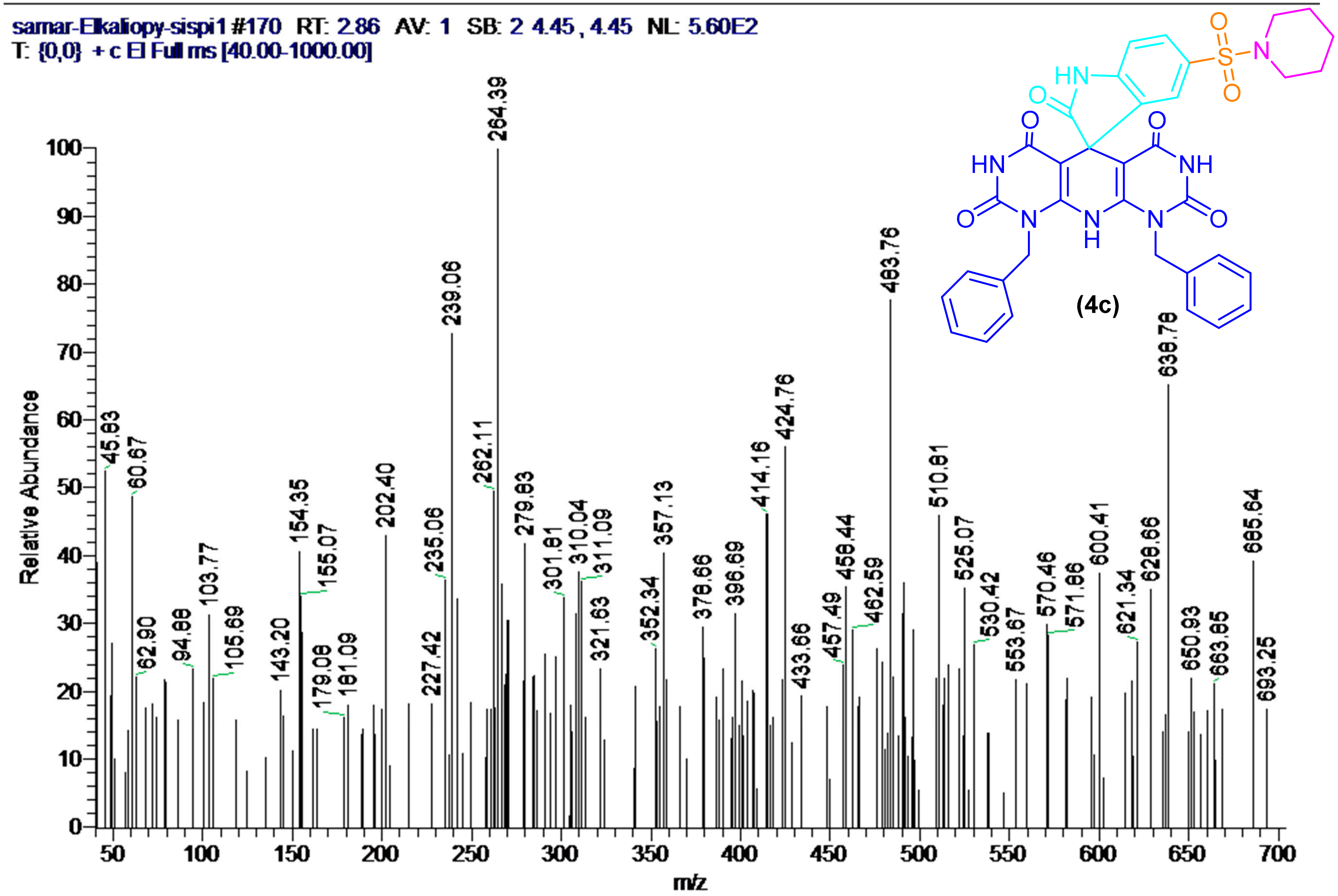
samar-Elkaliopy-sispi1 #170 RT: 2.86 AV: 1 SB: 2 4.45 , 4.45 NL: 5.60E2
T: {0,0} + c EI Full ms [40.00-1000.00]
Relative Abundance
m/z
264.39
239.06
483.76
638.76
424.76
45.83
60.67
414.16
262.11
510.81
202.40
154.35
279.83
685.64
357.13
600.41

(4c)

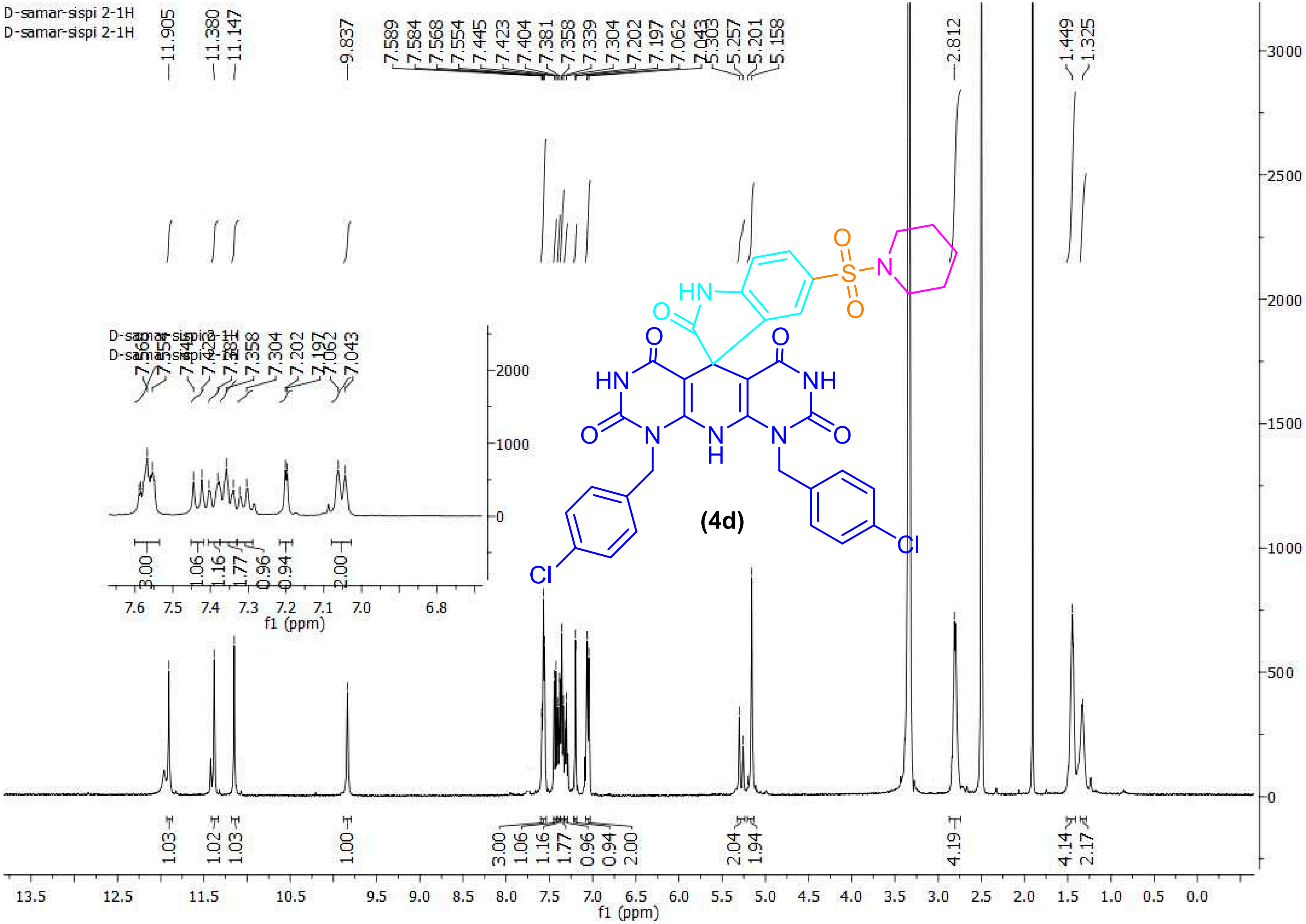
D-samar-sispi 2-1H
D-samar-sispi 2-1H
11.905
11.380
11.147
9.837
7.589
7.584
7.568
7.554
7.445
7.423
7.404
7.381
7.358
7.339
7.304
7.202
7.197
7.062
7.043
5.303
5.257
5.201
5.158
2.812
1.449
1.325
HN
O
S
N
O
HN
NH
N
N
H
Cl
Cl
(4d)
1.03
1.02
1.03
1.00
3.00
1.06
1.16
1.77
0.96
0.94
2.00
2.04
1.94
4.19
4.14
2.17
f1 (ppm)

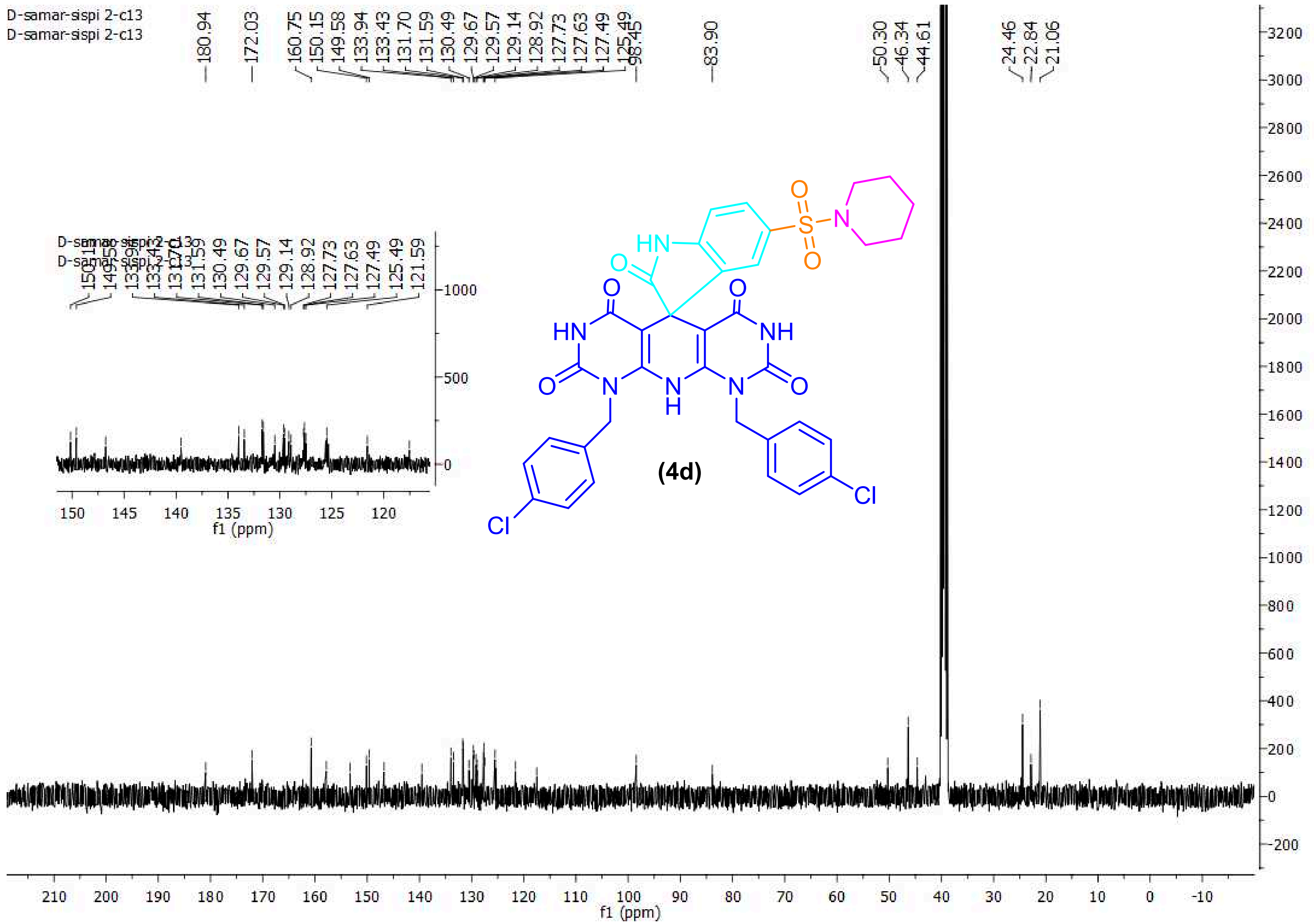
D-samar-sispi 2-c13
D-samar-sispi 2-c13
180.94
172.03
160.75
150.15
149.58
133.94
133.43
131.70
131.59
130.49
129.67
129.57
129.14
128.92
127.73
127.63
127.49
125.49
98.45
83.90
50.30
46.34
44.61
24.46
22.84
21.06
(4d)
f1 (ppm)

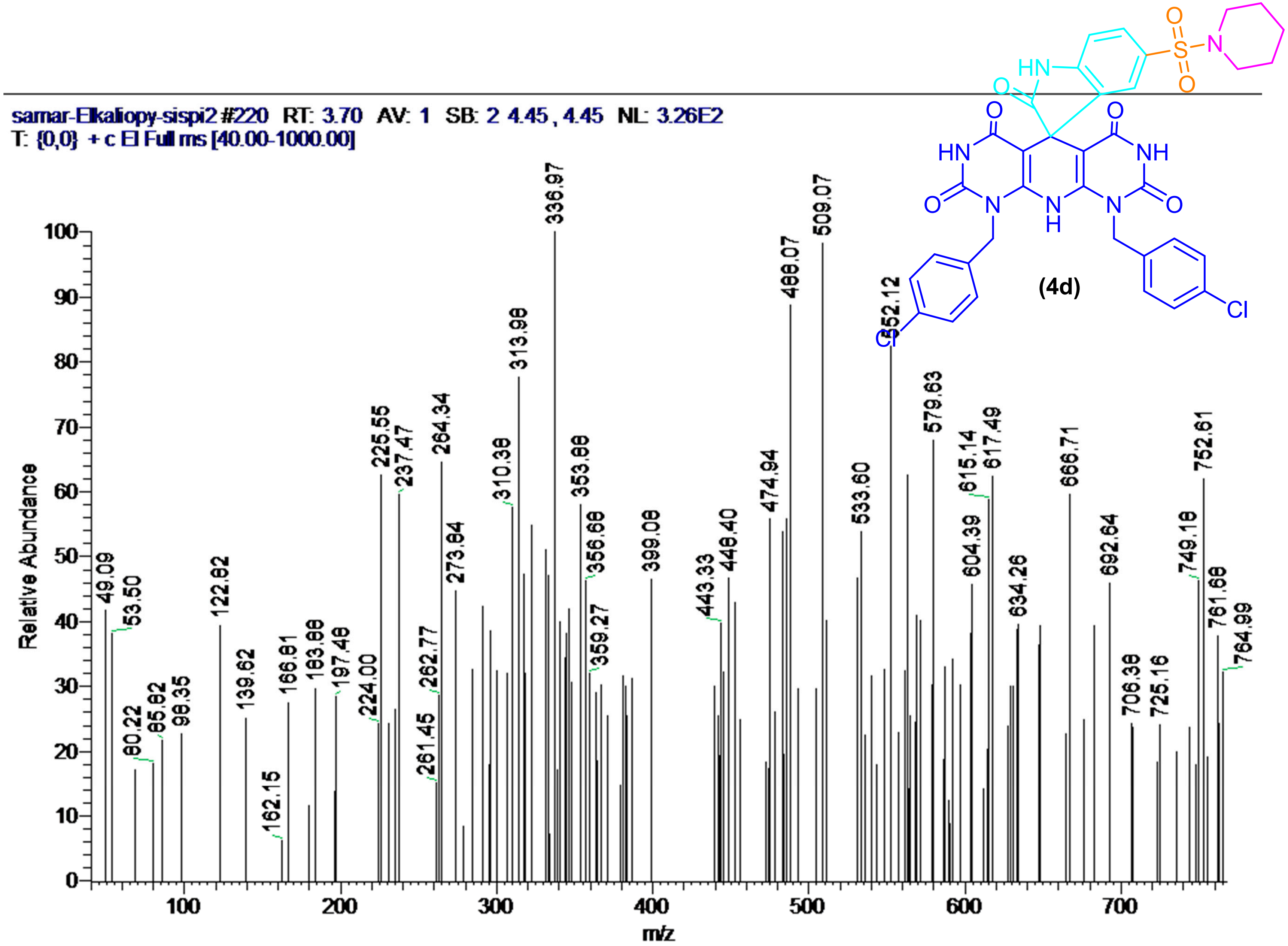
samar-Elkaliopy-sispi2 #220 RT: 3.70 AV: 1 SB: 2 4.45 , 4.45 NL: 3.26E2
T: {0,0} + c EI Full ms [40.00-1000.00]
Relative Abundance
m/z
49.09
53.50
60.22
65.62
98.35
122.62
139.62
162.15
166.81
183.66
197.48
224.00
225.55
237.47
261.45
262.77
264.34
273.84
310.38
313.98
336.97
353.66
356.68
359.27
399.06
443.33
448.40
474.94
488.07
509.07
533.60
552.12
579.63
604.39
615.14
617.49
634.26
666.71
692.64
706.38
725.16
749.18
752.61
761.68
764.99
HN
O
S
N
NH
Cl

(4d)

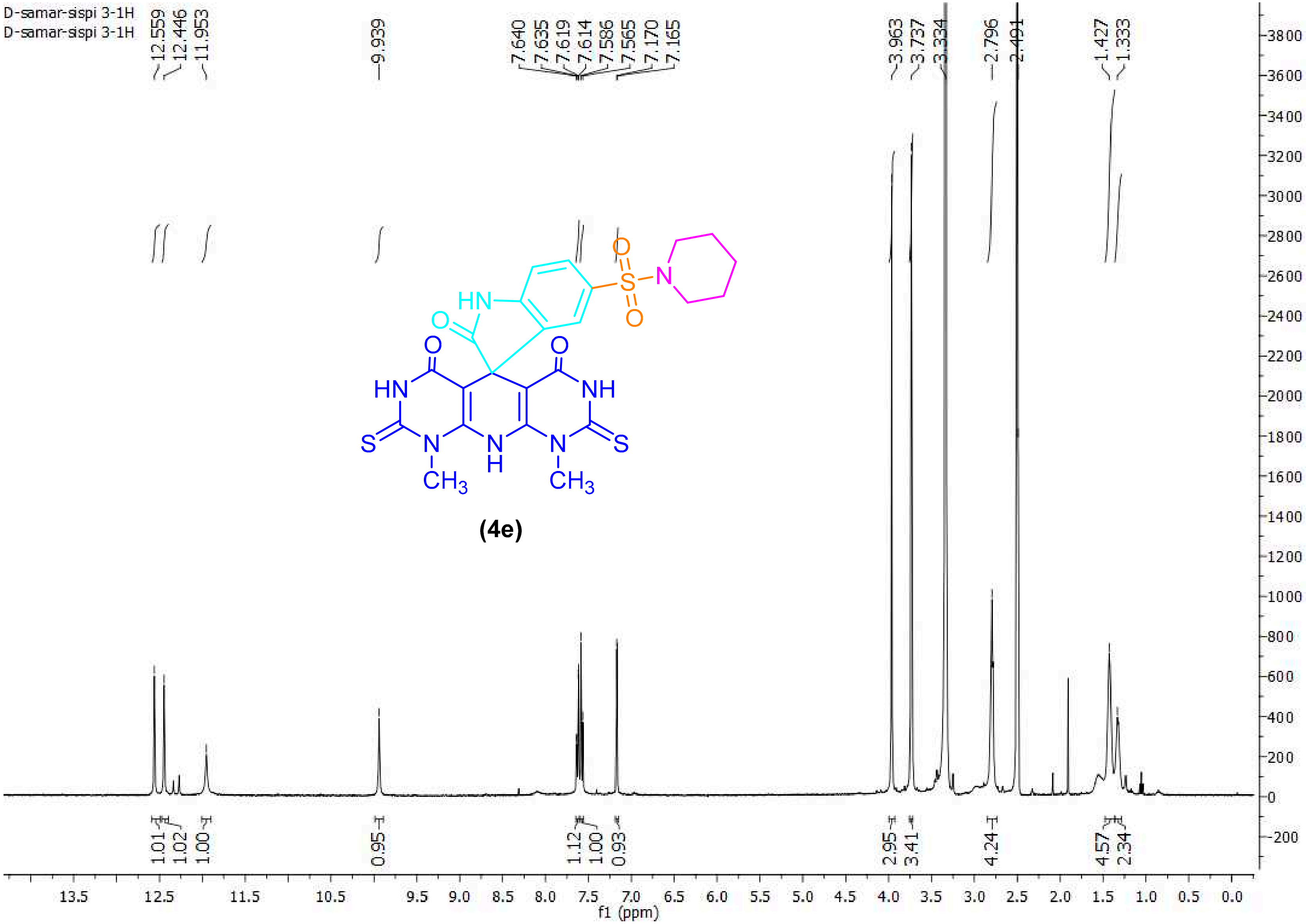
D-samar-sispi 3-1H
D-samar-sispi 3-1H
12.559
12.446
11.953
9.939
7.640
7.635
7.619
7.614
7.586
7.565
7.170
7.165
3.963
3.737
3.334
2.796
2.491
1.427
1.333
HN
O
O
S
N
O
HN
NH
O
S
N
N
H
N
S
CH3
CH3
(4e)
1.01
1.02
1.00
0.95
1.12
1.00
0.93
2.95
3.41
4.24
4.57
2.34
f1 (ppm)

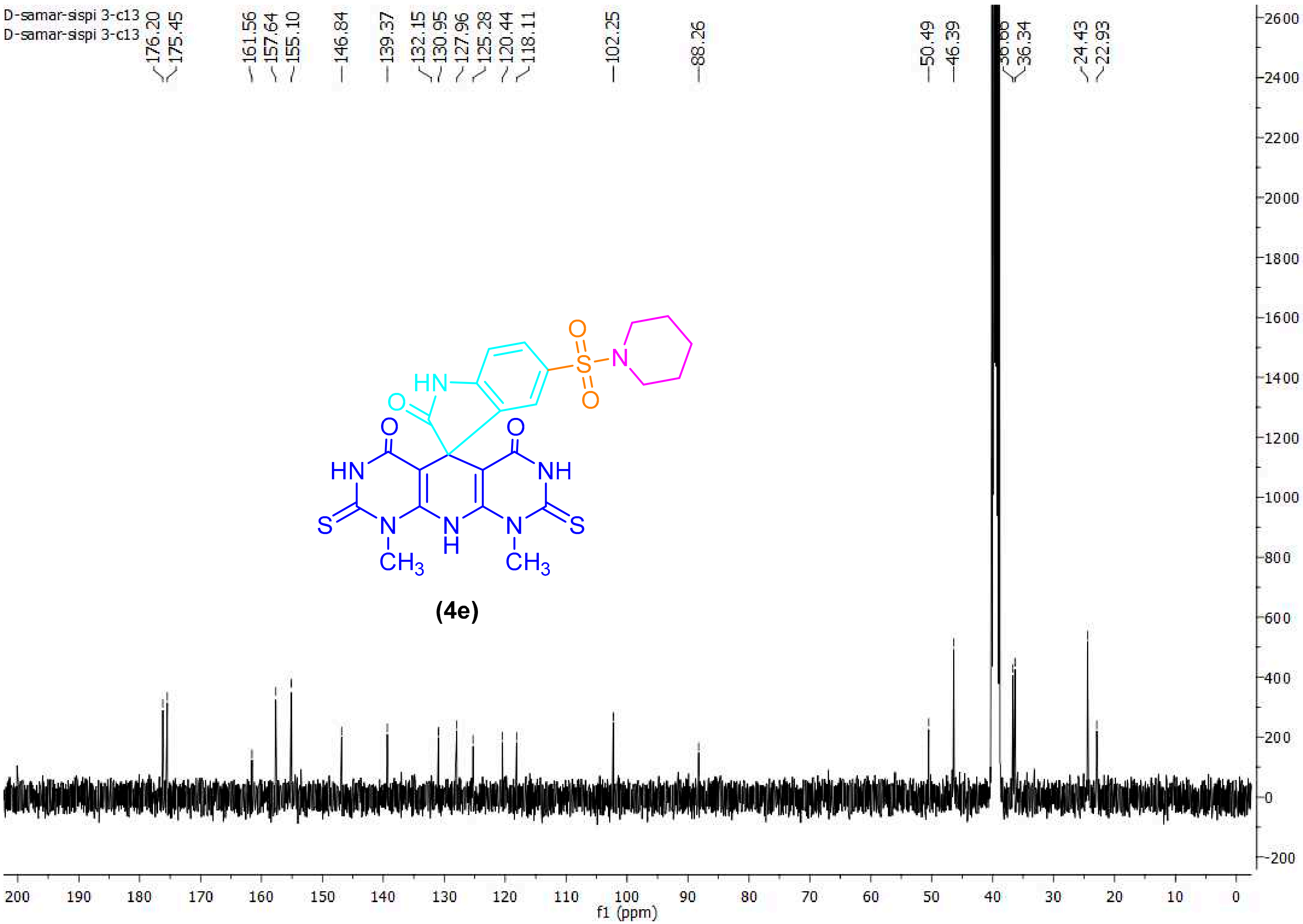
D-samar-sispi 3-c13
D-samar-sispi 3-c13
176.20
175.45
161.56
157.64
155.10
146.84
139.37
132.15
130.95
127.96
125.28
120.44
118.11
102.25
88.26
50.49
46.39
36.34
24.43
22.93
(4e)
f1 (ppm)

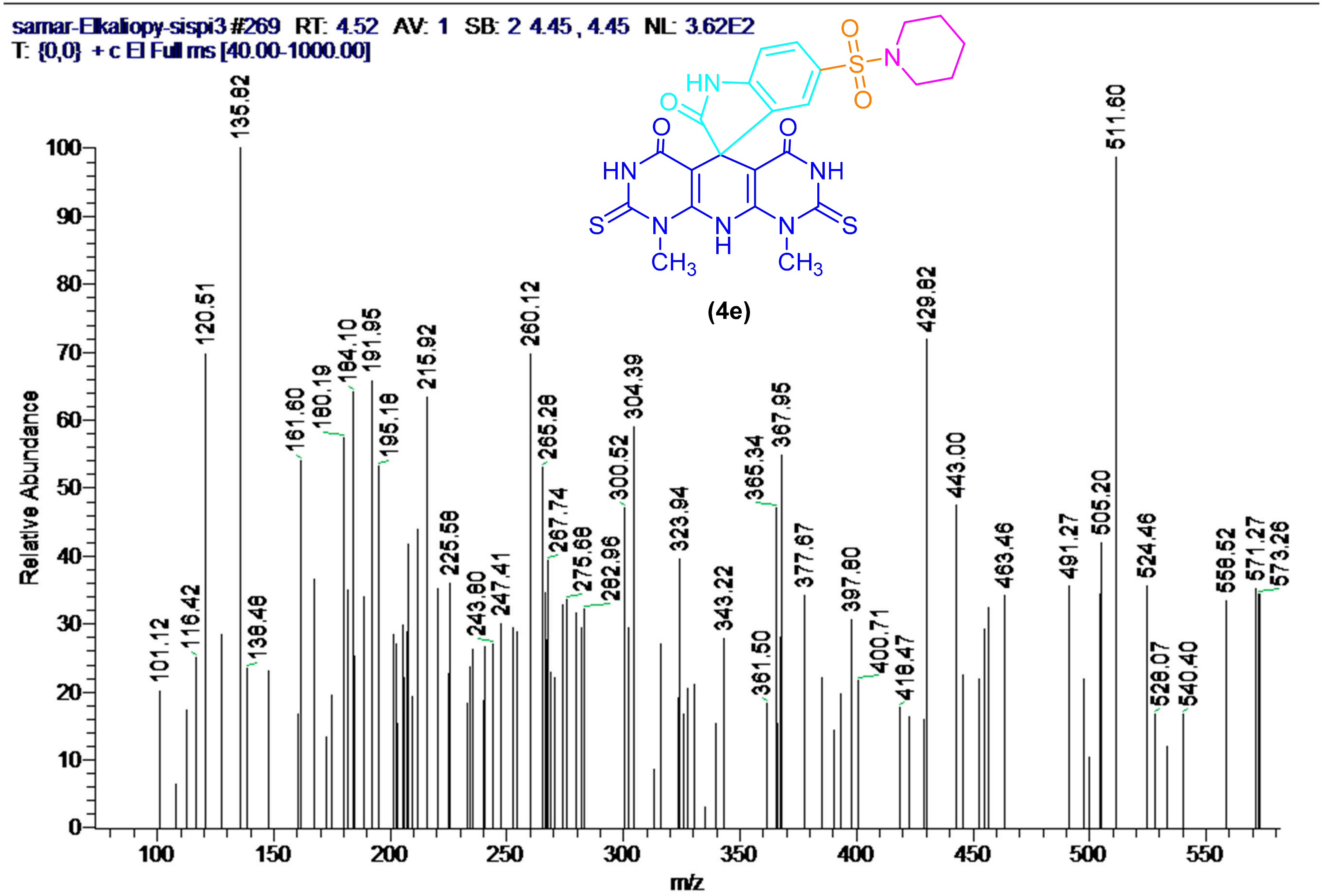
samar-Elkaliopy-sispi3 #269 RT: 4.52 AV: 1 SB: 2 4.45 , 4.45 NL: 3.62E2
T: {0,0} + c EI Full ms [40.00-1000.00]
(4e)
Relative Abundance
m/z
135.82
511.60
120.51
260.12
429.82
215.92
191.95
184.10
180.19
161.60
195.18
304.39
265.28
300.52
367.95
365.34
443.00
505.20
323.94
267.74
275.68
282.96
225.58
243.60
247.41
101.12
116.42
138.48
343.22
377.67
397.60
400.71
361.50
418.47
463.46
491.27
524.46
528.07
540.40
558.52
571.27
573.26

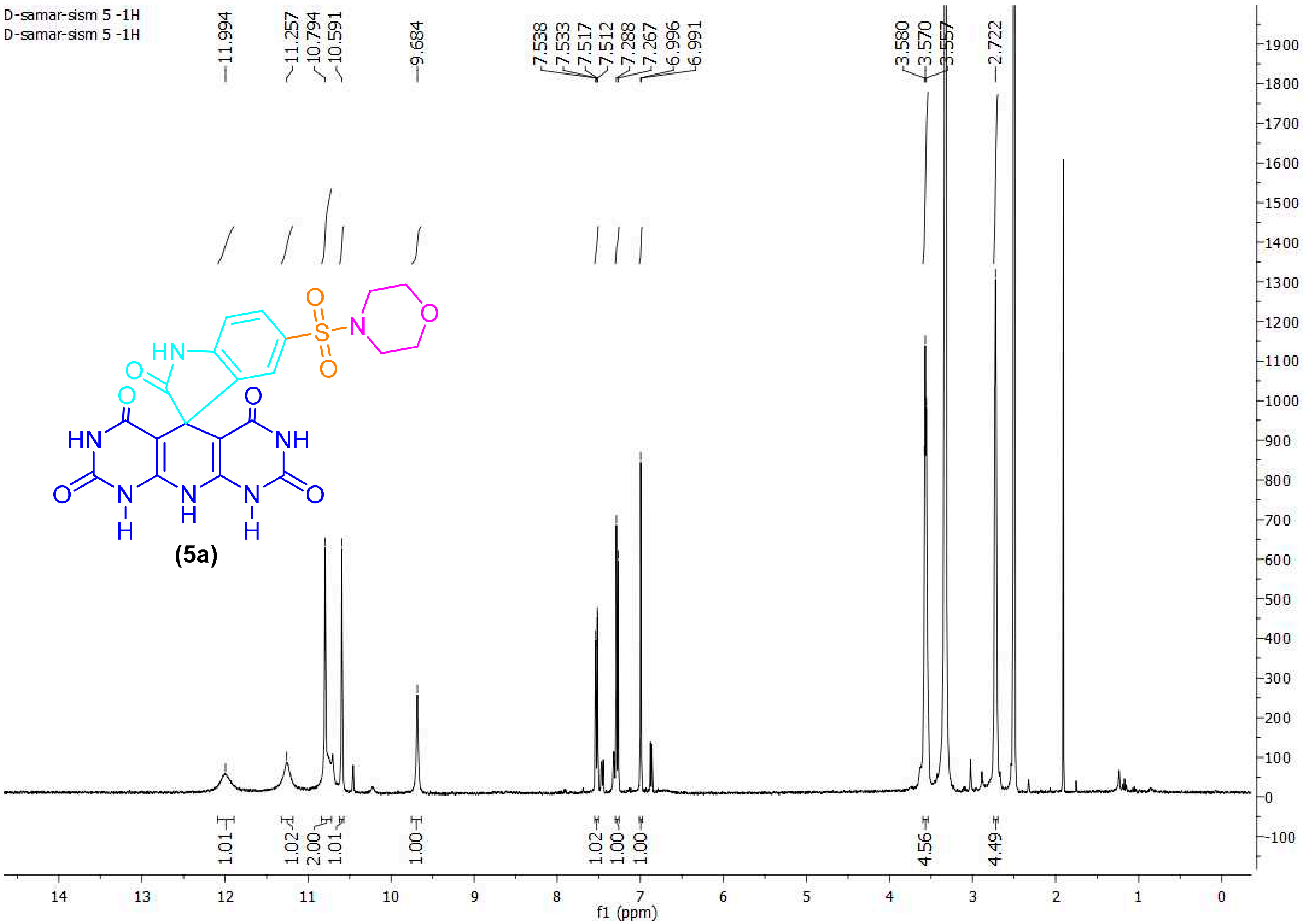

D-samar-sism 5 -1H
D-samar-sism 5 -1H
11.994
11.257
10.794
10.591
9.684
7.538
7.533
7.517
7.512
7.288
7.267
6.996
6.991
3.580
3.570
3.557
2.722
1.01
1.02
2.00
1.01
1.00
1.02
1.00
1.00
4.56
4.49
HN
HN
NH
N
H
O
S
(5a)
f1 (ppm)

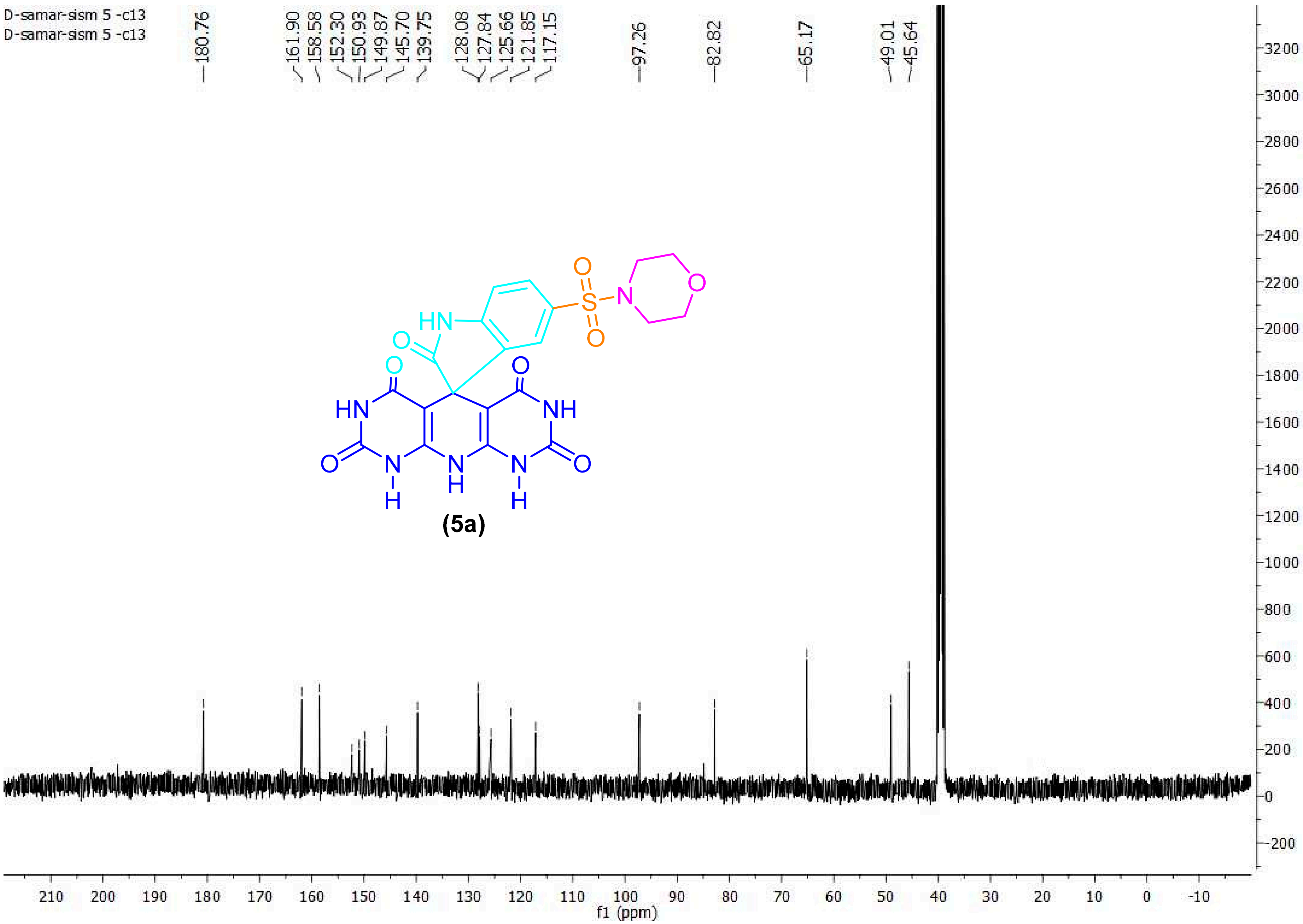

D-samar-sism 5 -c13
D-samar-sism 5 -c13
180.76
161.90
158.58
152.30
150.93
149.87
145.70
139.75
128.08
127.84
125.66
121.85
117.15
97.26
82.82
65.17
49.01
45.64
HN
O
S
N
NH
H
(5a)
f1 (ppm)

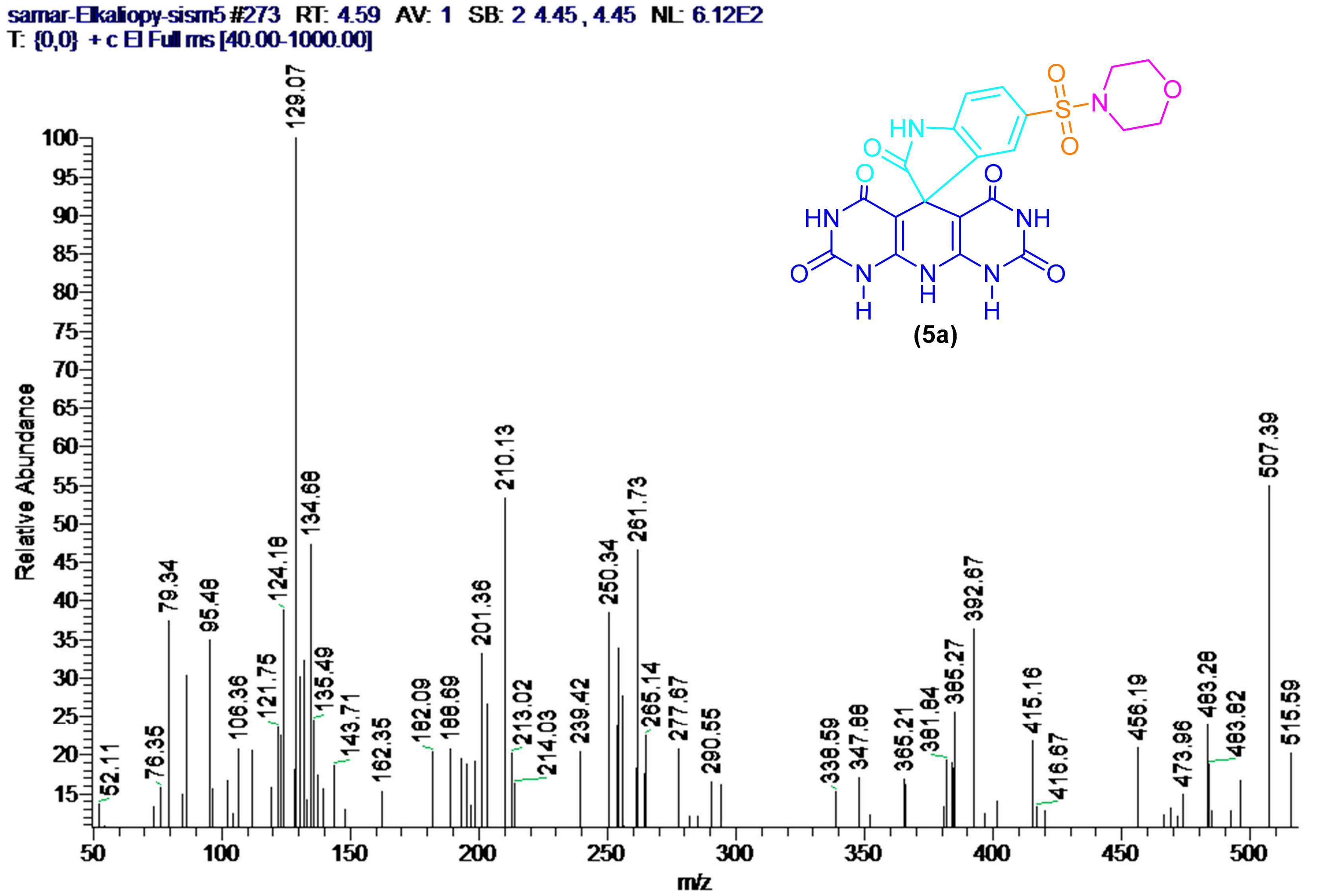
samar-Elkaliopy-sism5 #273 RT: 4.59 AV: 1 SB: 2 4.45 , 4.45 NL: 6.12E2
T: {0,0} + c EI Full ms [40.00-1000.00]
Relative Abundance
m/z
129.07
507.39
210.13
261.73
134.68
250.34
392.67
124.18
79.34
95.48
201.36
385.27
483.28
415.16
135.49
121.75
106.36
143.71
162.35
182.09
188.69
213.02
214.03
239.42
265.14
277.67
290.55
338.59
347.88
365.21
381.84
416.67
456.19
473.96
493.82
515.59
76.35
52.11
(5a)

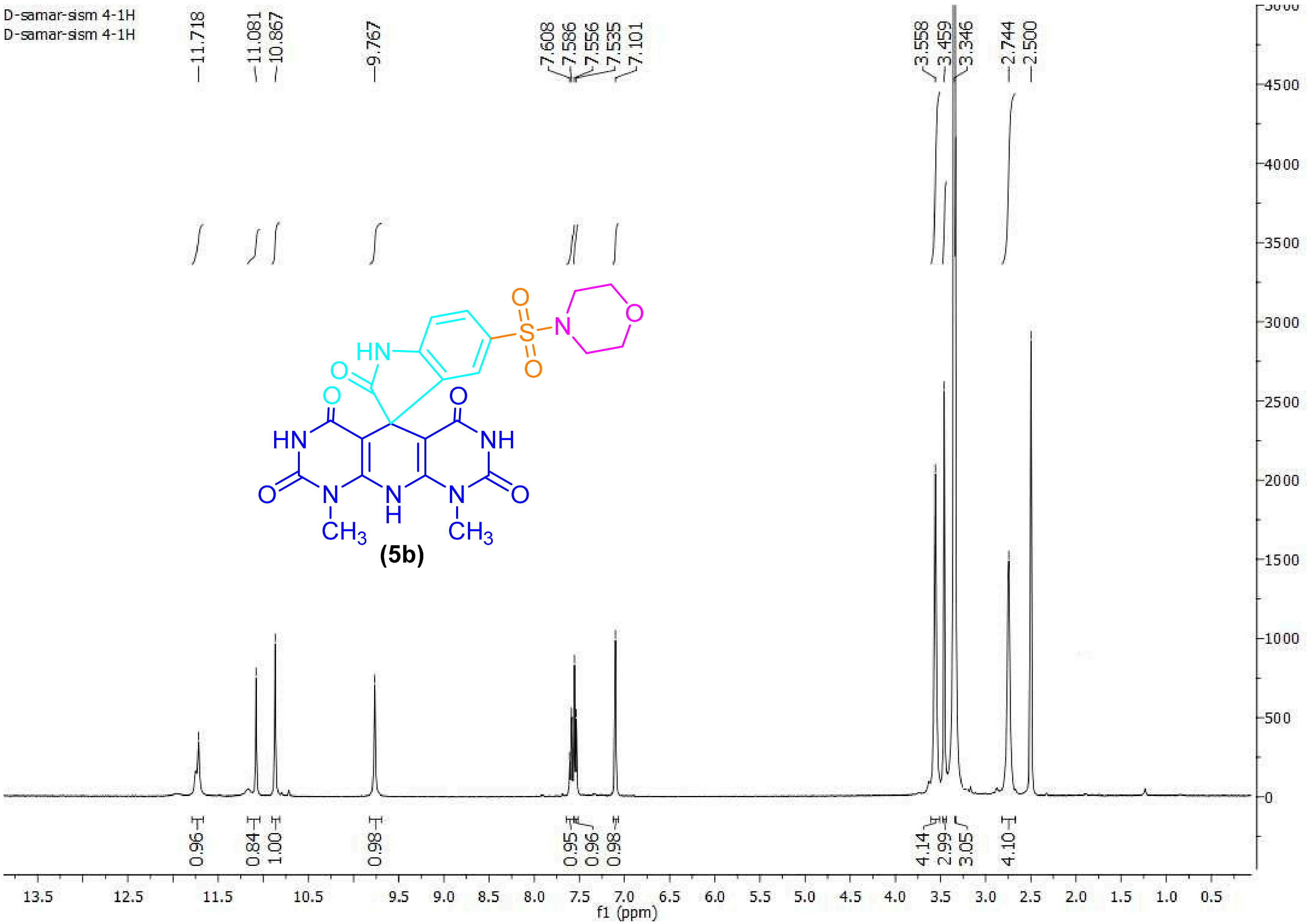
D-samar-sism 4-1H
D-samar-sism 4-1H
11.718
11.081
10.867
9.767
7.608
7.586
7.556
7.535
7.101
3.558
3.459
3.346
2.744
2.500
HN
O
S
N
O
HN
NH
N
N
H
CH3
CH3
(5b)
0.96
0.84
1.00
0.98
0.95
0.96
0.98
4.14
2.99
3.05
4.10
f1 (ppm)

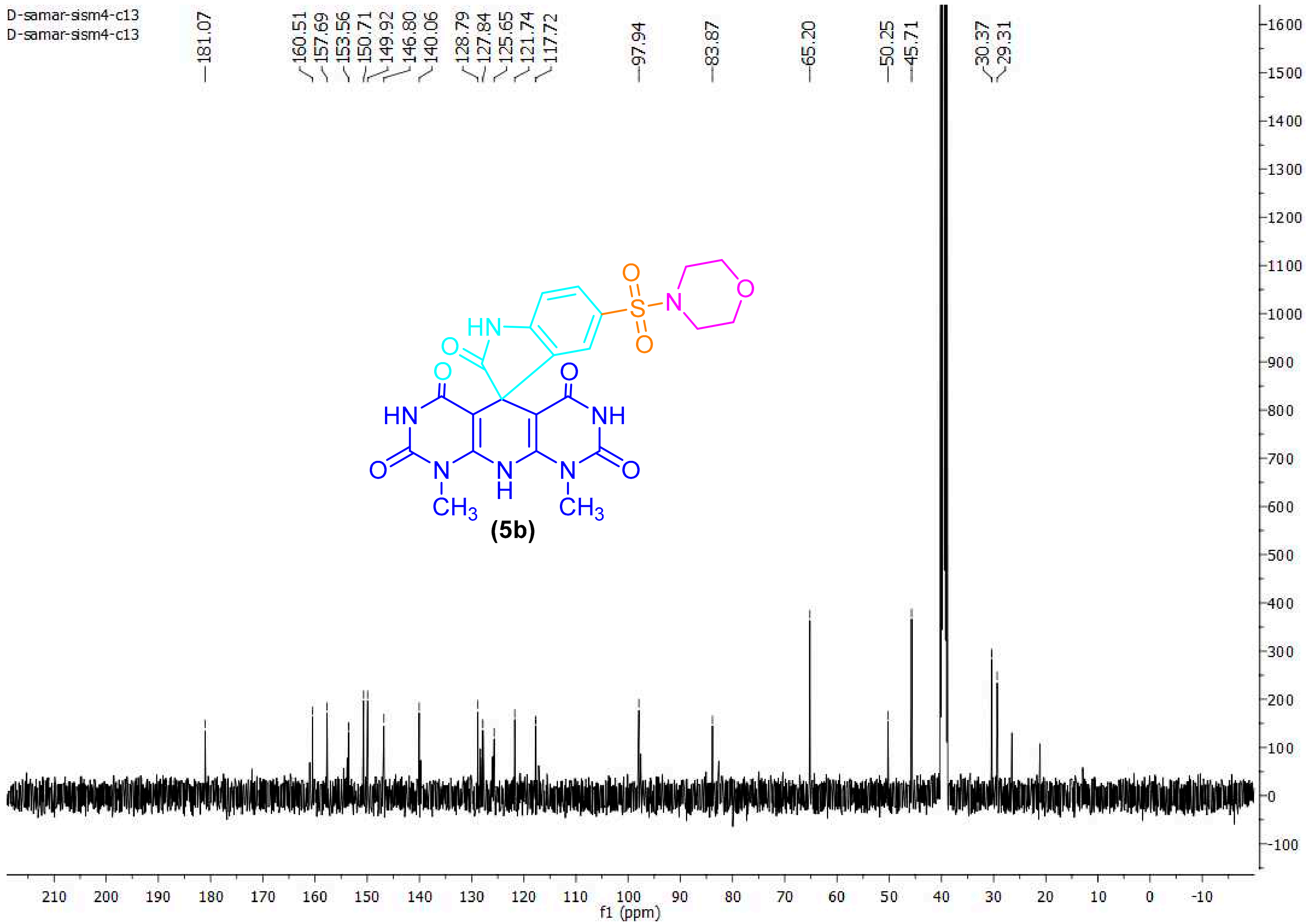
D-samar-sism4-c13
D-samar-sism4-c13
181.07
160.51
157.69
153.56
150.71
149.92
146.80
140.06
128.79
127.84
125.65
121.74
117.72
97.94
83.87
65.20
50.25
45.71
30.37
29.31
(5b)
f1 (ppm)

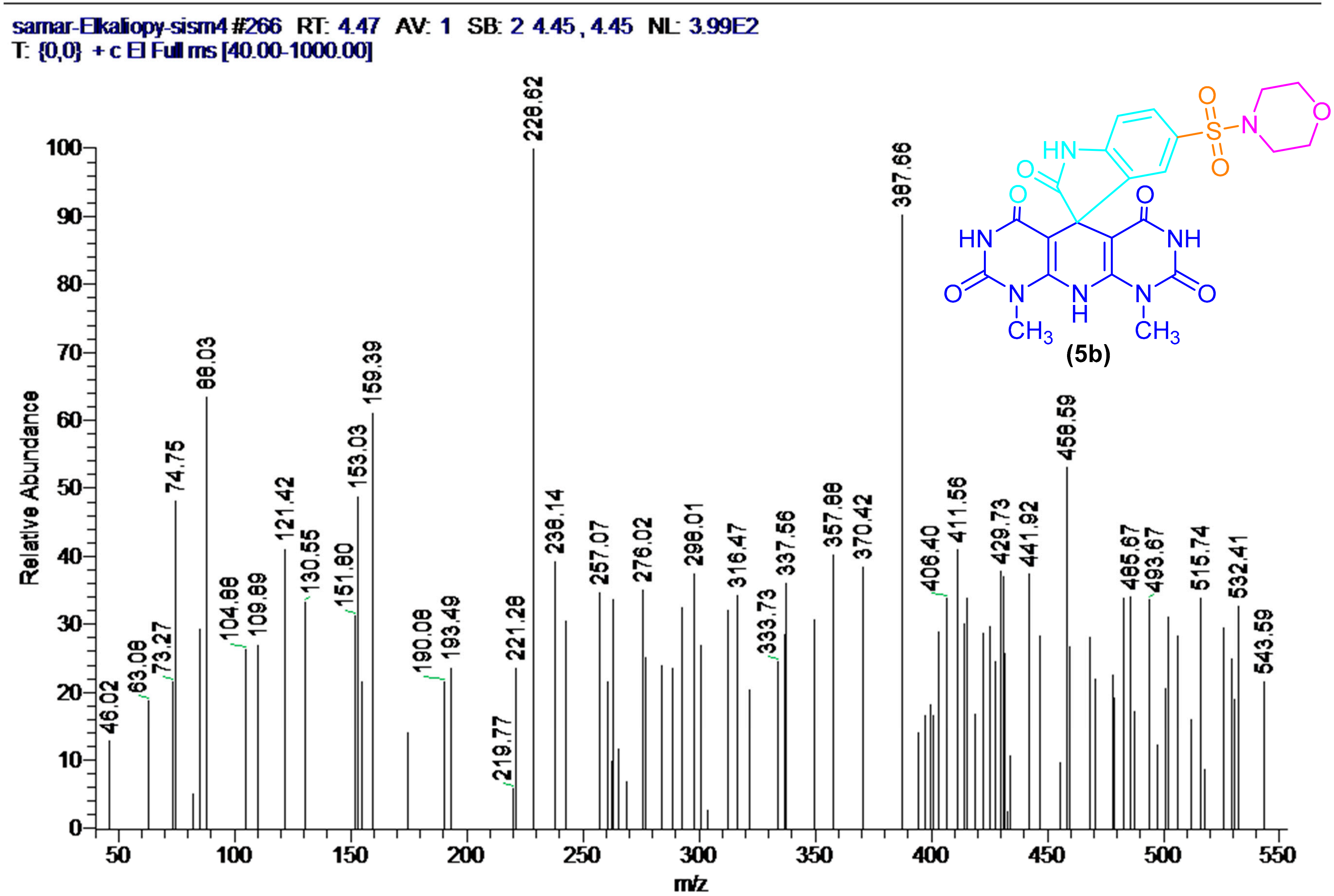

samar-Elkaliopy-sism4 #266 RT: 4.47 AV: 1 SB: 2 4.45 , 4.45 NL: 3.99E2
T: {0,0} + c EI Full ms [40.00-1000.00]
Relative Abundance
m/z
228.62
387.66
88.03
159.39
458.59
153.03
74.75
121.42
411.56
357.88
370.42
429.73
441.92
298.01
236.14
316.47
257.07
276.02
337.56
406.40
485.67
493.67
515.74
532.41
130.55
151.80
104.88
109.89
193.49
190.08
221.28
333.73
543.59
73.27
63.08
46.02
219.77
(5b)

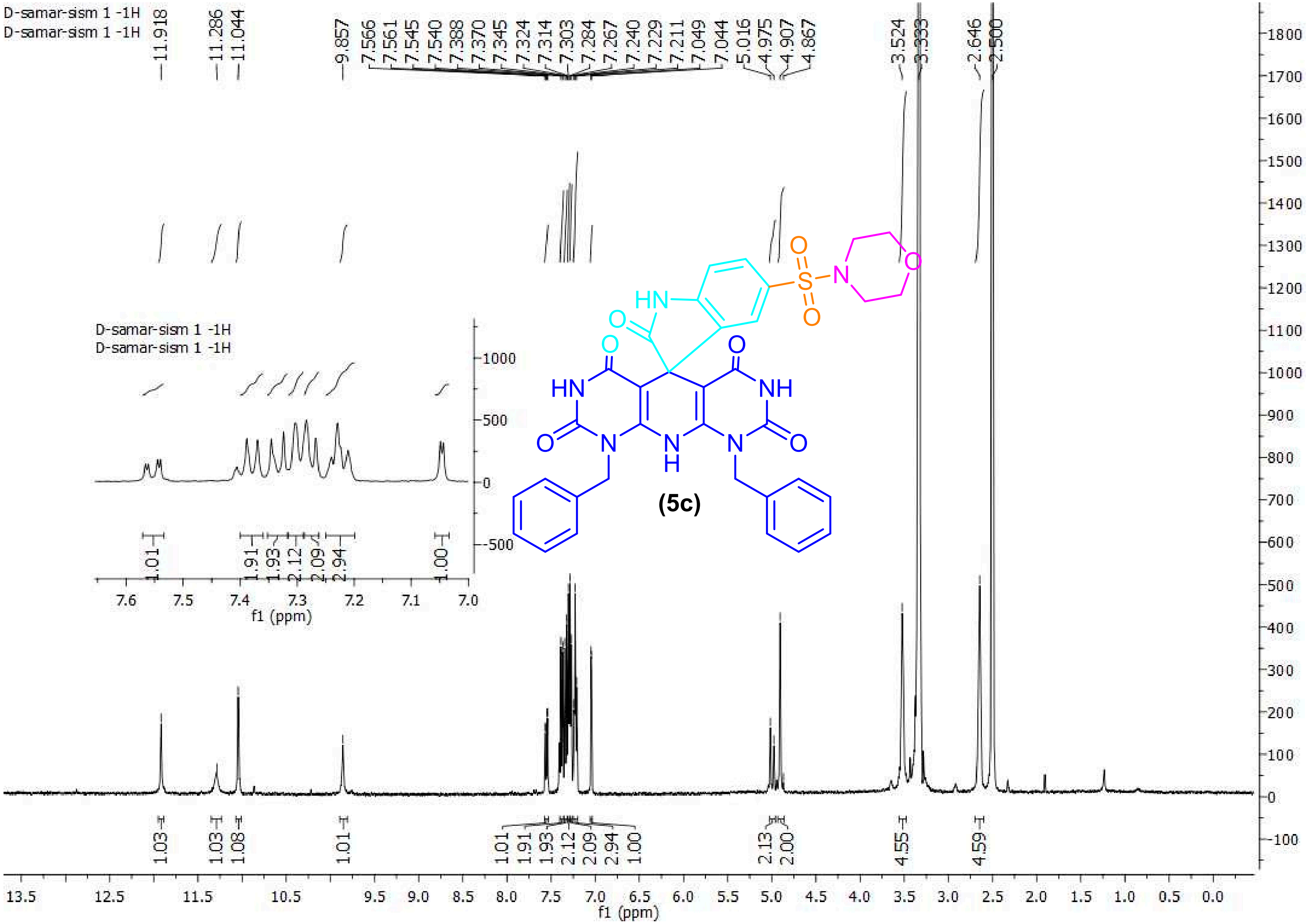
D-samar-sism 1 -1H
D-samar-sism 1 -1H
11.918
11.286
11.044
9.857
7.566
7.561
7.545
7.540
7.388
7.370
7.345
7.324
7.314
7.303
7.284
7.267
7.240
7.229
7.211
7.049
7.044
5.016
4.975
4.907
4.867
3.524
3.333
2.646
2.500
1.03
1.03
1.08
1.01
1.01
1.91
1.93
2.12
2.09
2.94
1.00
2.13
2.00
4.55
4.59
f1 (ppm)
(5c)

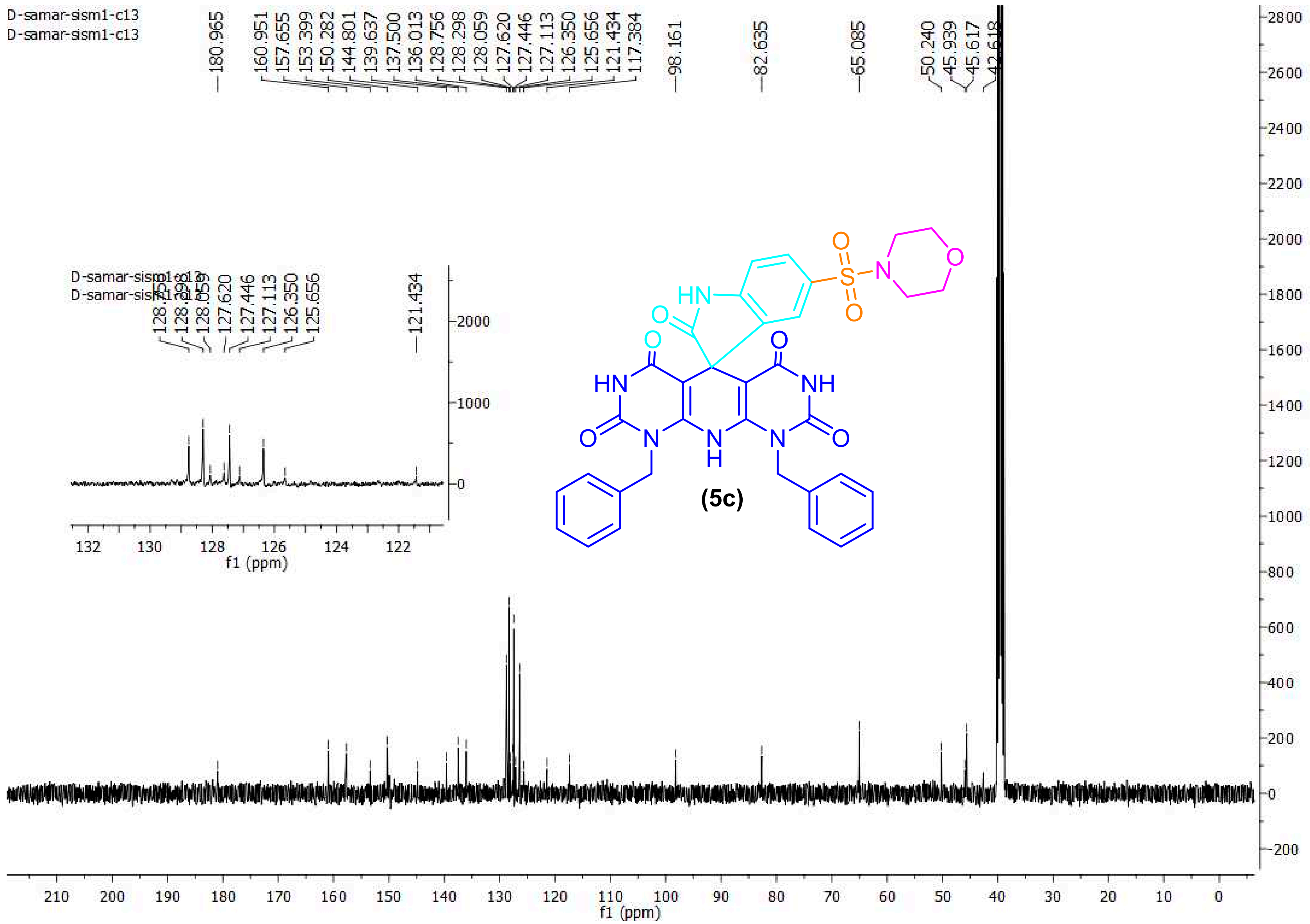

D-samar-sism1-c13
D-samar-sism1-c13
180.965
160.951
157.655
153.399
150.282
144.801
139.637
137.500
136.013
128.756
128.298
128.059
127.620
127.446
127.113
126.350
125.656
121.434
117.384
98.161
82.635
65.085
50.240
45.939
45.617
42.618
(5c)
f1 (ppm)

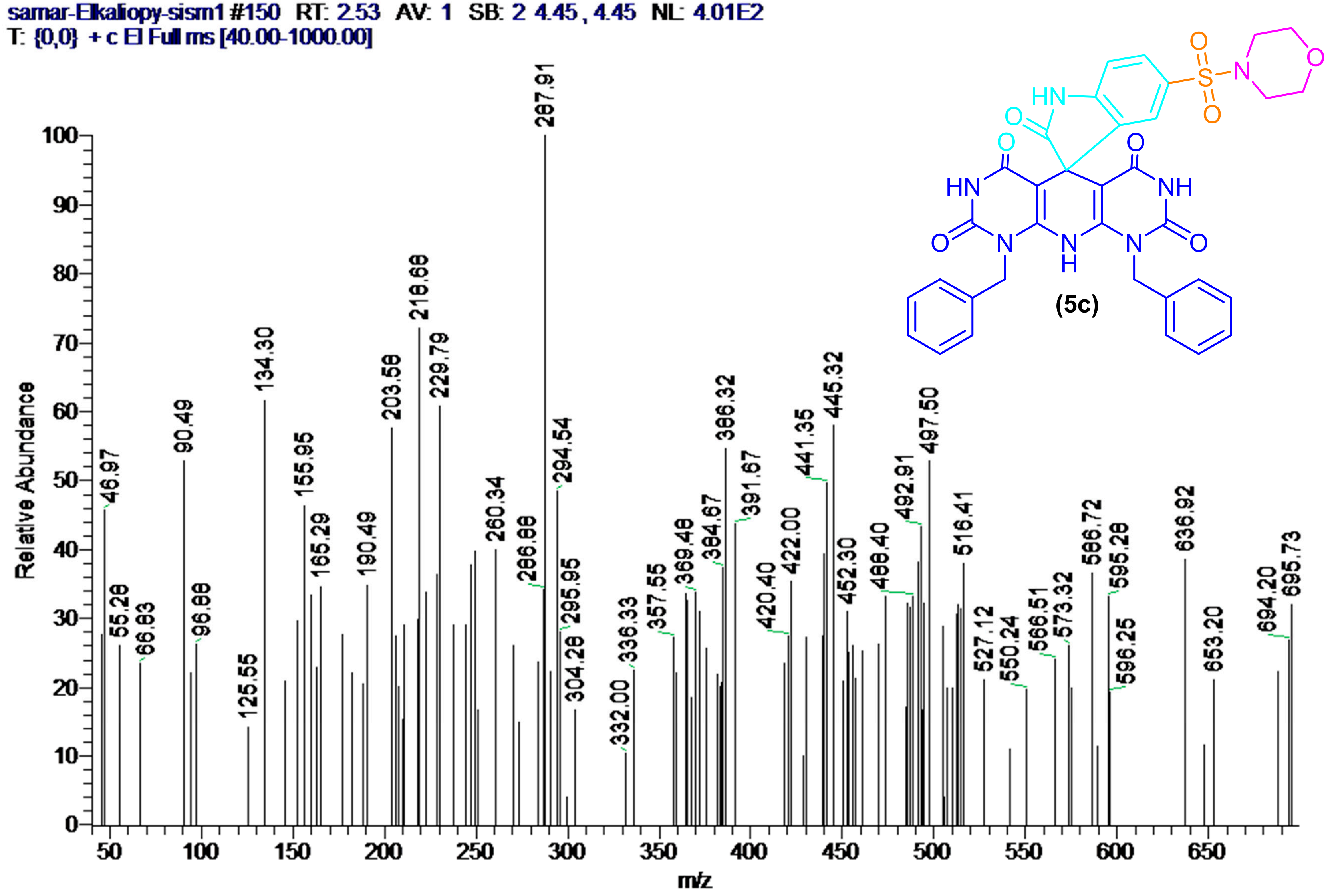
samar-Elkaliopy-sism1 #150 RT: 2.53 AV: 1 SB: 2 4.45 , 4.45 NL: 4.01E2
T: {0,0} + c EI Full ms [40.00-1000.00]
Relative Abundance
m/z
287.91
218.68
134.30
229.79
203.58
445.32
386.32
497.50
90.49
441.35
294.54
155.95
391.67
46.97
492.91
260.34
165.29
190.49
364.67
636.92
595.28
586.72
422.00
516.41
369.48
488.40
266.66
357.55
295.95
452.30
420.40
55.28
573.32
566.51
695.73
694.20
96.68
336.33
66.63
527.12
596.25
653.20
550.24
304.28
125.55
332.00
HN
O
S
N
O
NH
(5c)

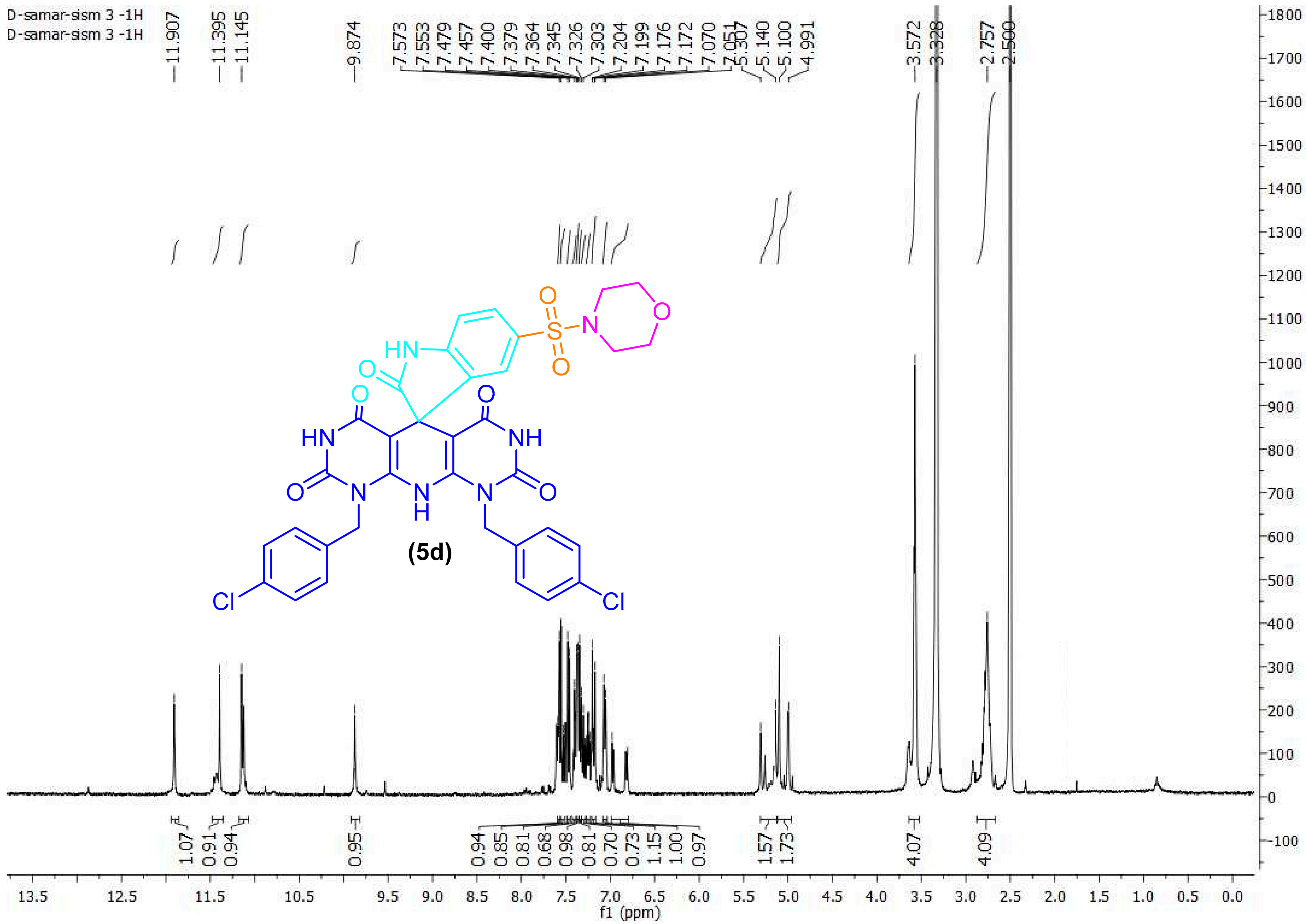

D-samar-sism 3 -1H
D-samar-sism 3 -1H
11.907
11.395
11.145
9.874
7.573
7.553
7.479
7.457
7.400
7.379
7.364
7.345
7.326
7.303
7.204
7.199
7.176
7.172
7.070
7.051
5.307
5.140
5.100
4.991
3.572
3.328
2.757
2.500
1.07
0.91
0.94
0.95
0.94
0.85
0.81
0.68
0.98
0.81
0.70
0.73
1.15
1.00
0.97
1.57
1.73
4.07
4.09
HN
O
S
N
O
O
O
O
O
HN
NH
O
N
N
H
N
O
(5d)
Cl
Cl
f1 (ppm)

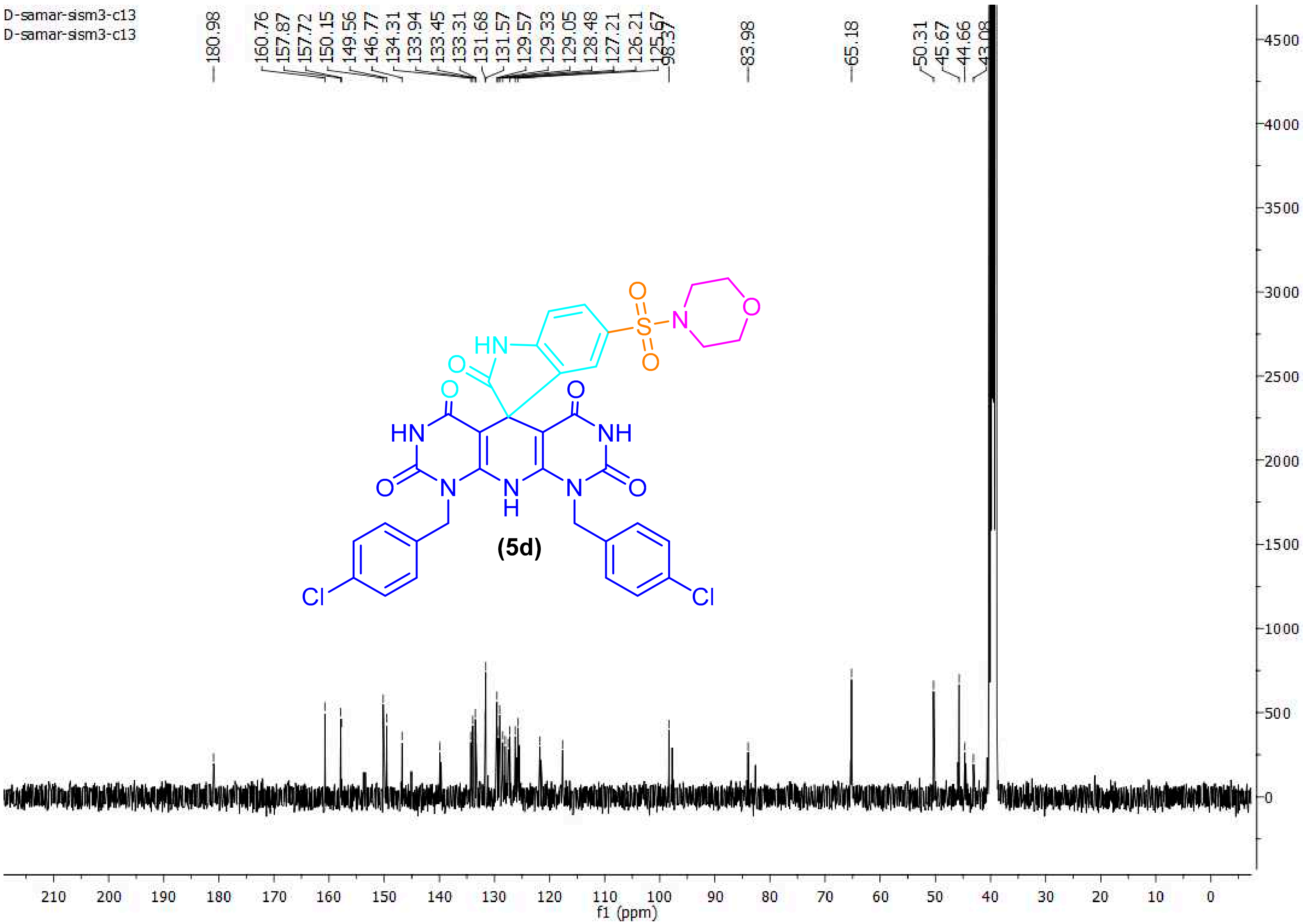

D-samar-sism3-c13
D-samar-sism3-c13
180.98
160.76
157.87
157.72
150.15
149.56
146.77
134.31
133.94
133.45
133.31
131.68
131.57
129.57
129.33
129.05
128.48
127.21
126.21
125.67
98.37
83.98
65.18
50.31
45.67
44.66
43.08
(5d)
f1 (ppm)

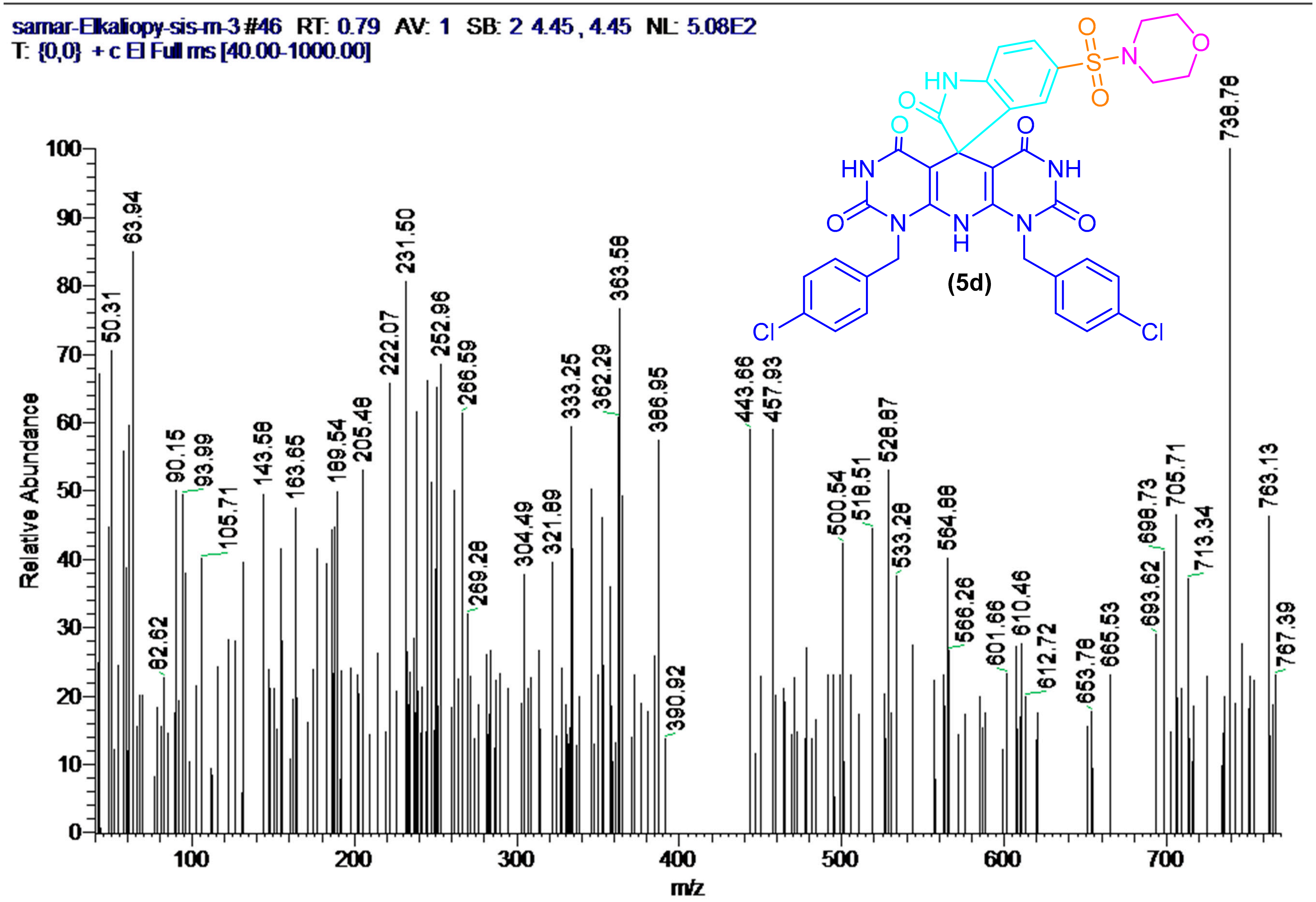
samar-Elkaliopy-sis-m-3 #46 RT: 0.79 AV: 1 SB: 2 4.45 , 4.45 NL: 5.08E2
T: {0,0} + c EI Full ms [40.00-1000.00]
Relative Abundance
m/z
(5d)
HN
O
S
N
O
NH
H
Cl
738.78
63.94
231.50
363.58
50.31
222.07
252.96
266.59
333.25
362.29
386.95
443.66
457.93
90.15
93.99
143.58
163.65
189.54
205.48
528.67
705.71
763.13
698.73
713.34
564.88
500.54
518.51
533.26
105.71
304.49
321.89
269.28
566.26
601.66
610.46
612.72
693.62
665.53
653.78
767.39
82.62
390.92

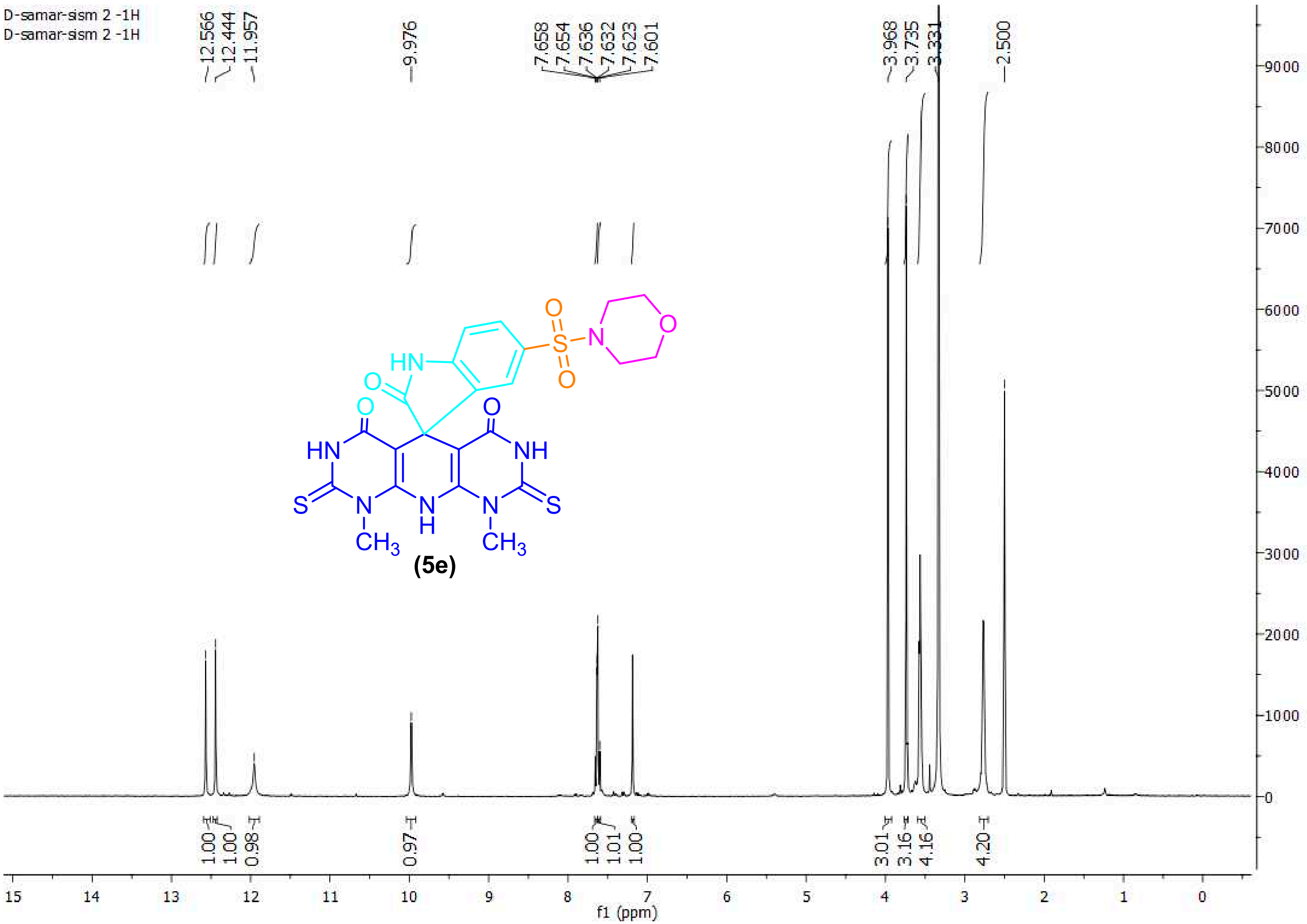
D-samar-sism 2 -1H
D-samar-sism 2 -1H
12.566
12.444
11.957
9.976
7.658
7.654
7.636
7.632
7.623
7.601
3.968
3.735
3.331
2.500
1.00
1.00
0.98
0.97
1.00
1.01
1.00
3.01
3.16
4.16
4.20
(5e)
f1 (ppm)

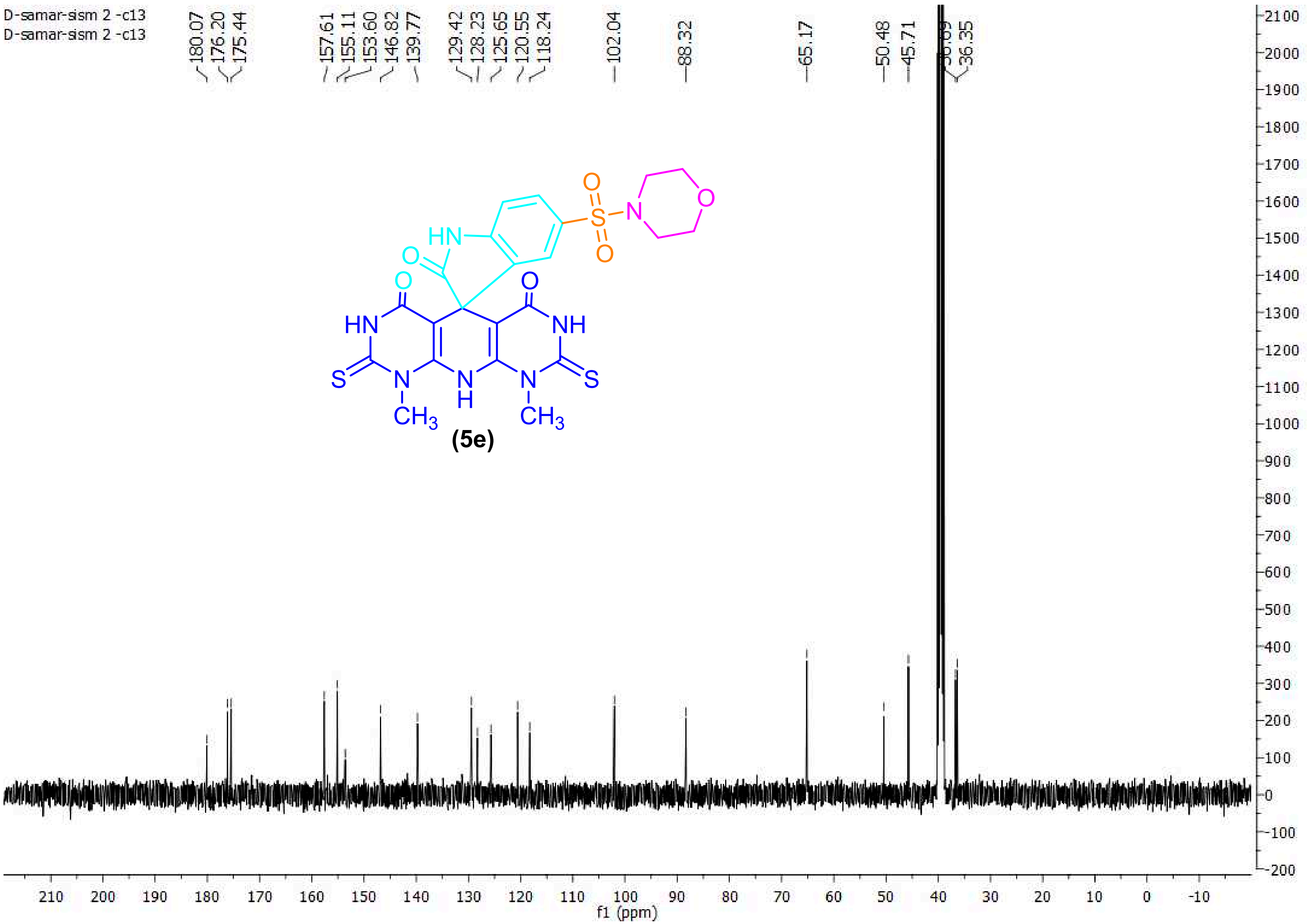
D-samar-sism 2 -c13
D-samar-sism 2 -c13
180.07
176.20
175.44
157.61
155.11
153.60
146.82
139.77
129.42
128.23
125.65
120.55
118.24
102.04
88.32
65.17
50.48
45.71
36.35
HN
O
S
N
NH
H
CH3
(5e)
f1 (ppm)

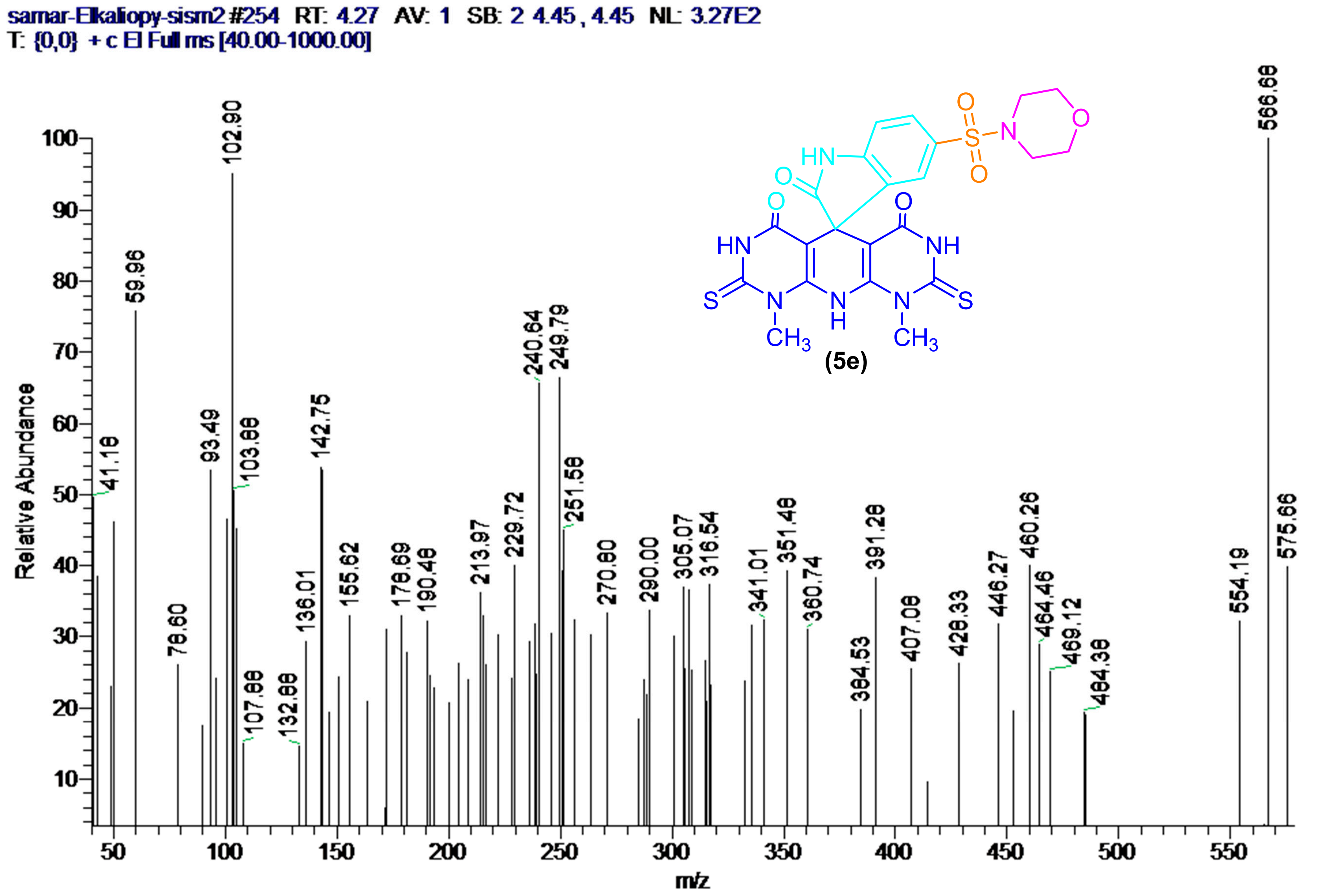
samar-Elkaliopy-sism2 #254 RT: 4.27 AV: 1 SB: 2 4.45 , 4.45 NL: 3.27E2
T: {0,0} + c EI Full ms [40.00-1000.00]
Relative Abundance
m/z
566.68
102.90
59.96
240.64
249.79
142.75
93.49
103.88
41.16
251.58
575.66
460.26
229.72
213.97
305.07
316.54
351.48
391.28
155.62
178.69
190.48
270.80
290.00
341.01
360.74
446.27
464.46
469.12
428.33
407.08
384.53
484.38
554.19
136.01
78.60
107.88
132.88
HN
O
O
S
O
N
O
HN
O
NH
S
N
N
H
N
S
CH3
CH3
(5e)

IC$_{50}$ graphs

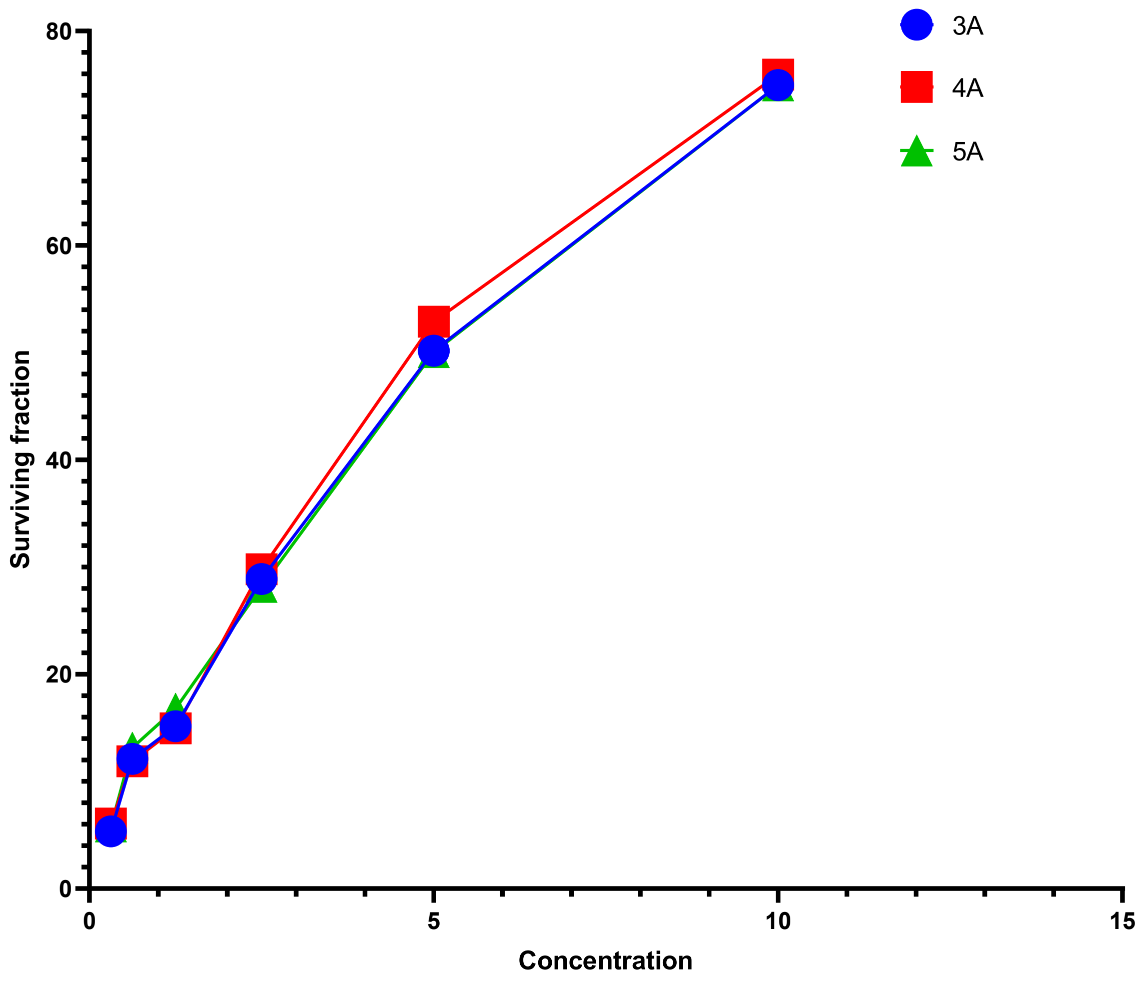

80
60
40
20
0
0
5
10
15
Surviving fraction
Concentration
3A
4A
5A

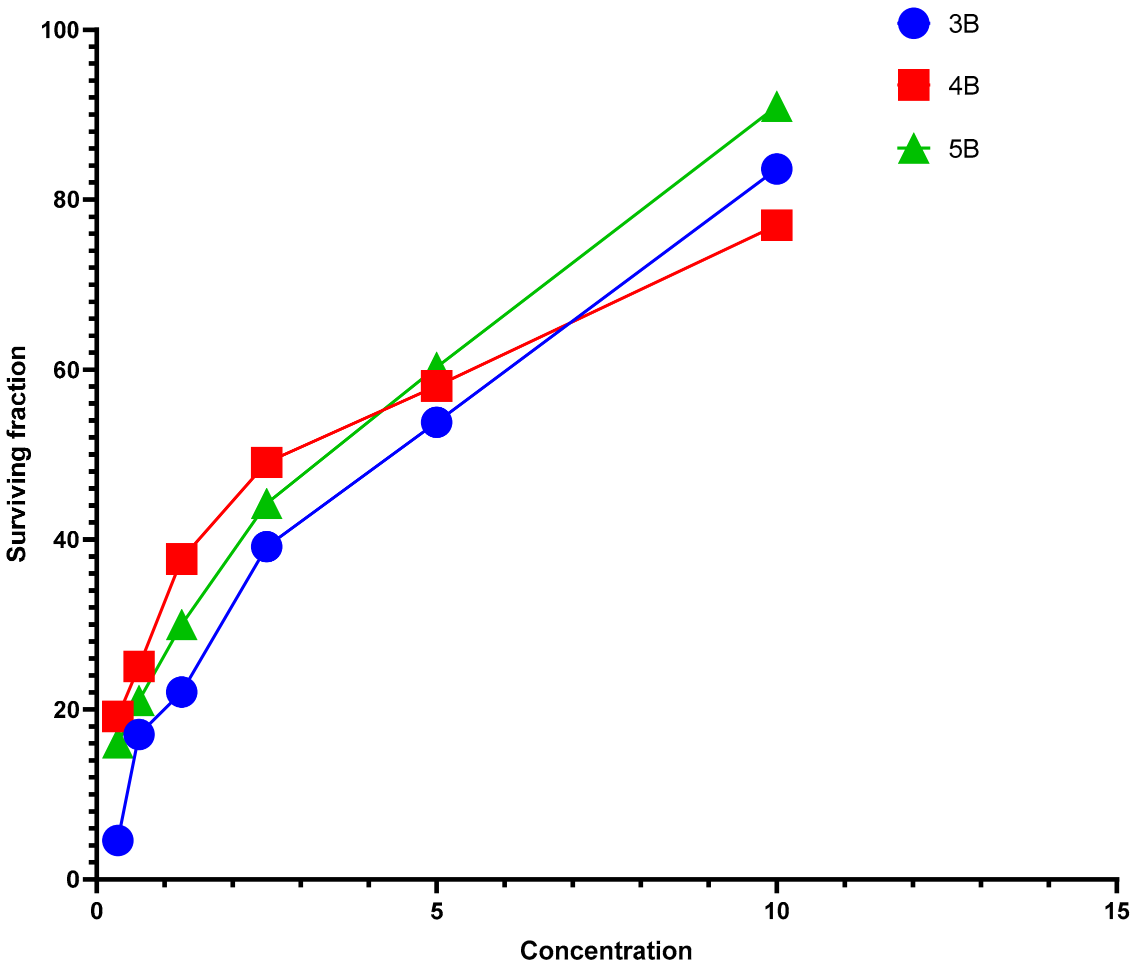

3B
4B
5B
Surviving fraction
100
80
60
40
20
0
0
5
10
15
Concentration

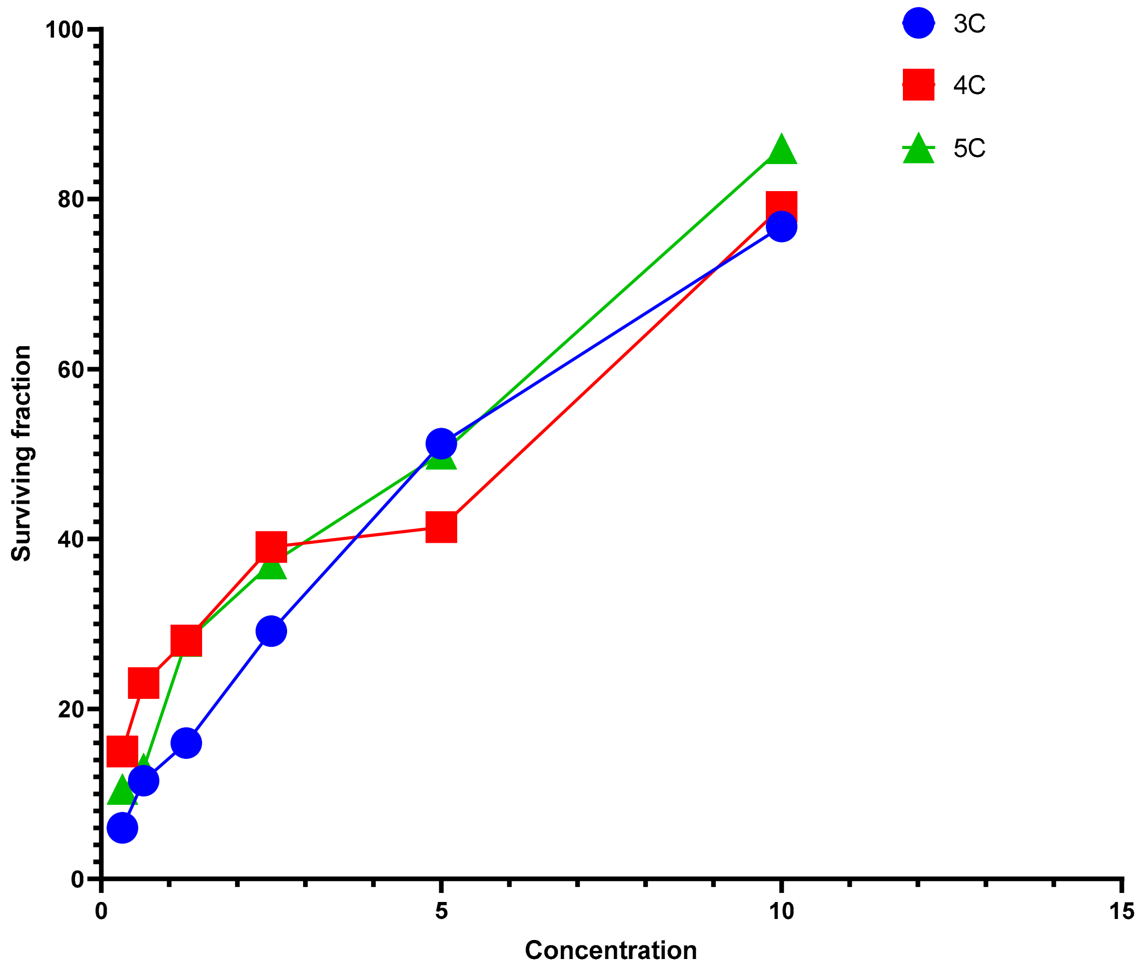
3C
4C
5C
Surviving fraction
100
80
60
40
20
0
0
5
10
15
Concentration

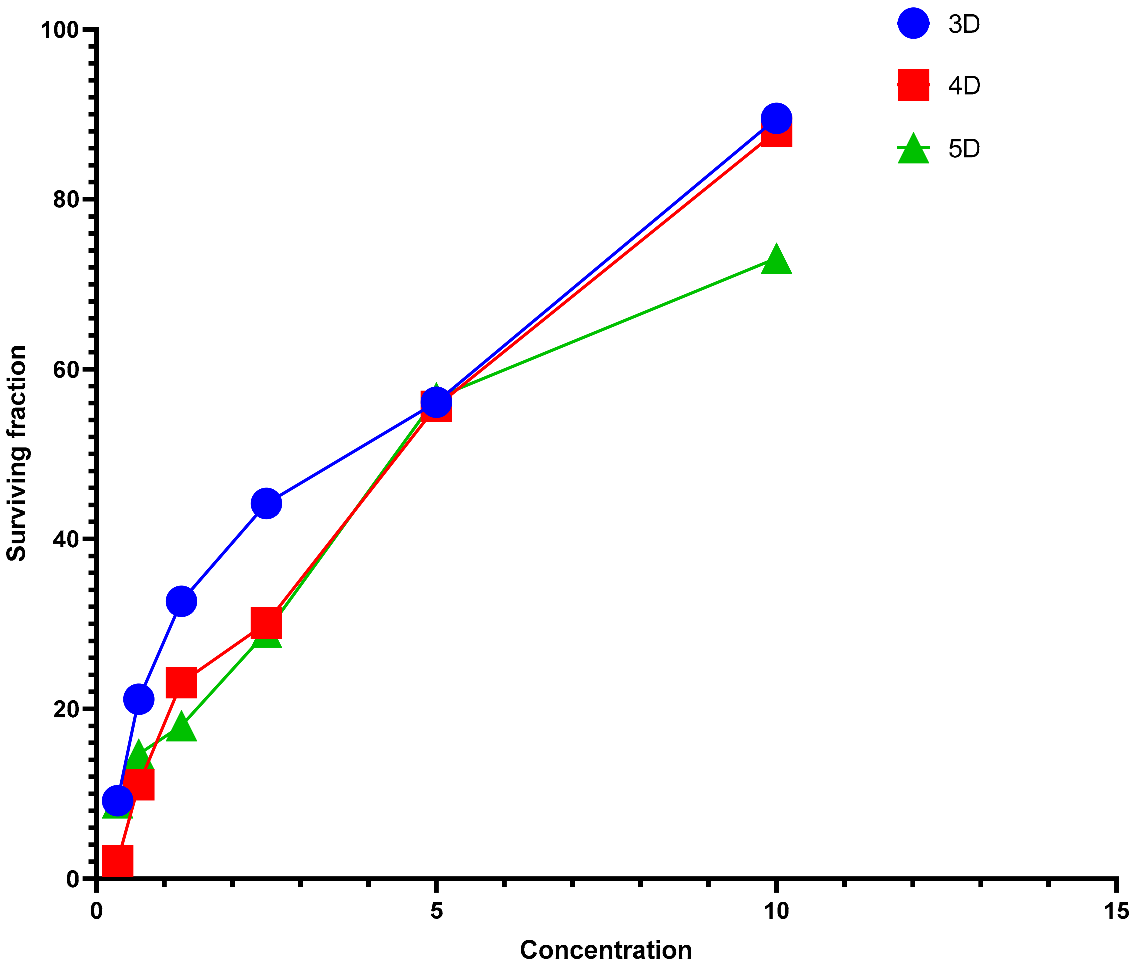

3D
4D
5D
Surviving fraction
100
80
60
40
20
0
0
5
10
15
Concentration

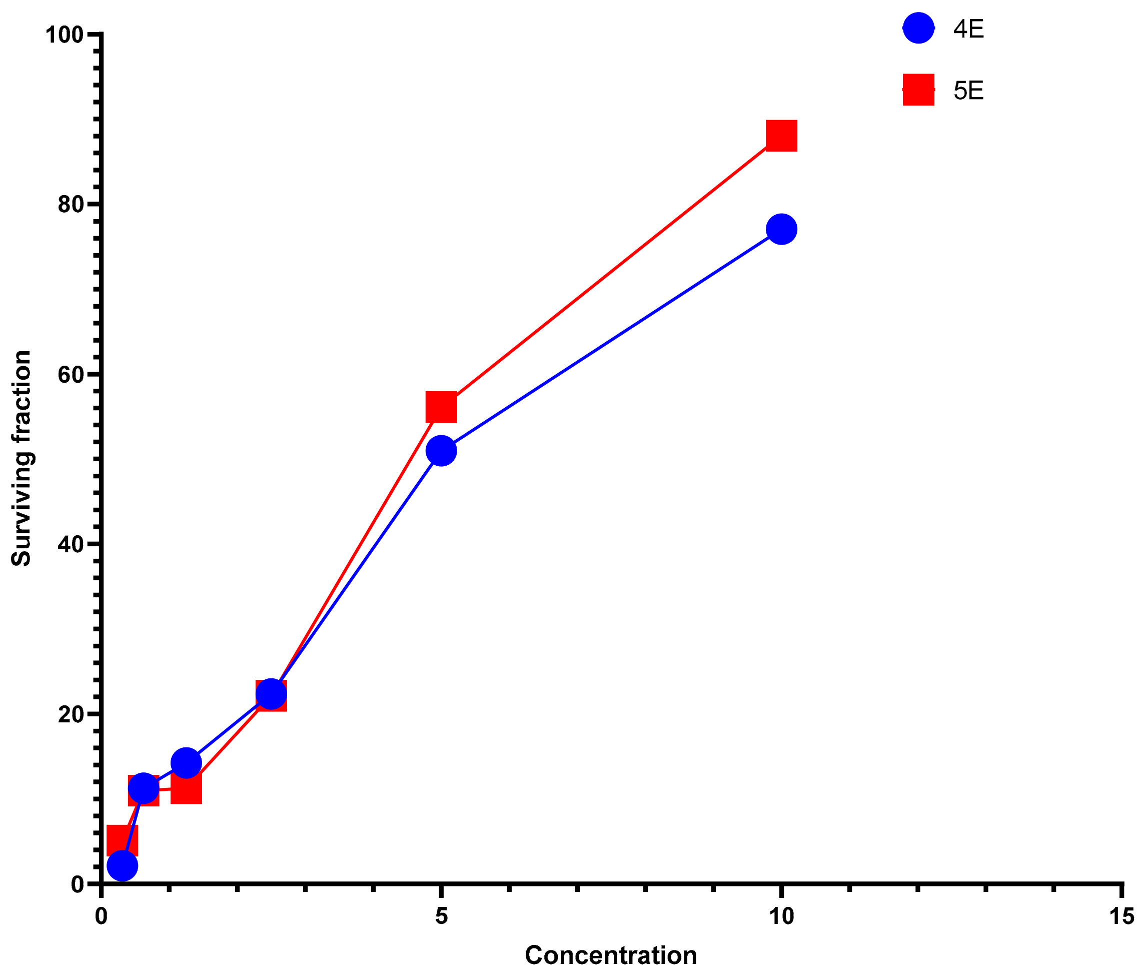

Surviving fraction
Concentration
0
20
40
60
80
100
0
5
10
15
4E
5E

Anti-COVID Cpd report

Cytotoxicity assay:

3A	Conc (micro g/µl)	Cytotoxicity%
	1000	74.9480
	500	50.2564
	250	28.876276
	125	15.212
	62.5	12.1323
	31.2	5.35241

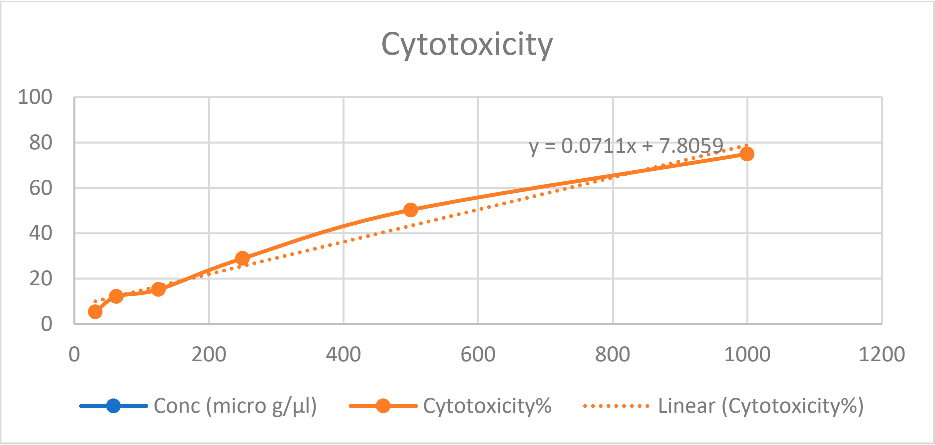

4A	Conc (micro g/µl)	Cytotoxicity%
	1000	75.9821
	500	52.8768
	250	29.78287
	125	14.97829
	62.5	11.87209
	31.2	6.16757

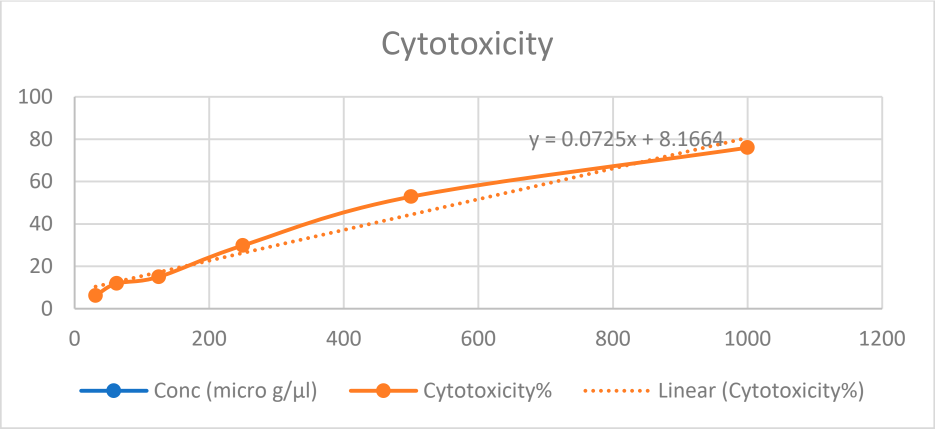

5A	Conc (micro g/µl)	Cytotoxicity%
	1000	74.8721
	500	50.13435
	250	28.23546
	125	16.8754
	62.5	13.12375
	31.2	5.83231

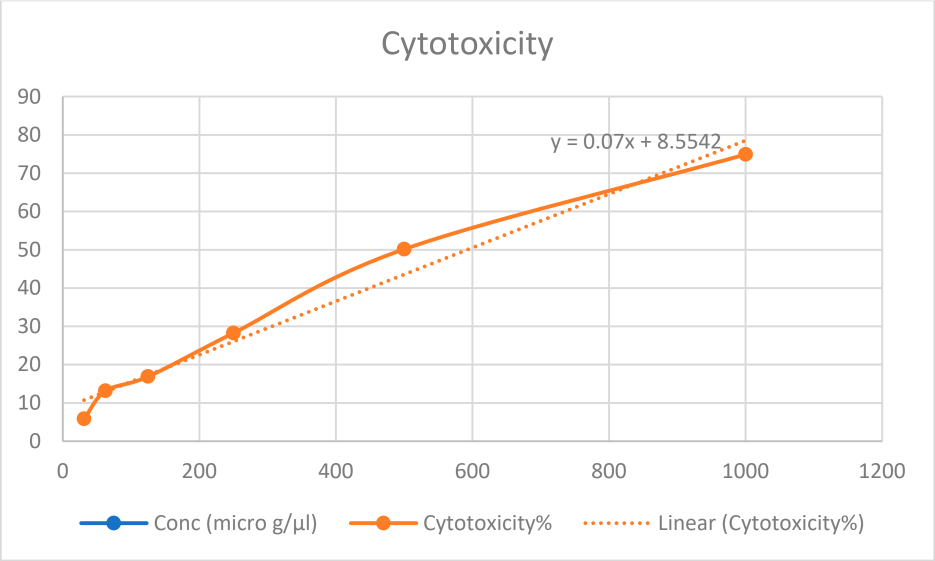

3C	Conc (micro g/µl)	Cytotoxicity%
	1000	76.7863
	500	51.2423
	250	29.2411
	125	15.95432
	62.5	11.6224
	31.2	6.02111

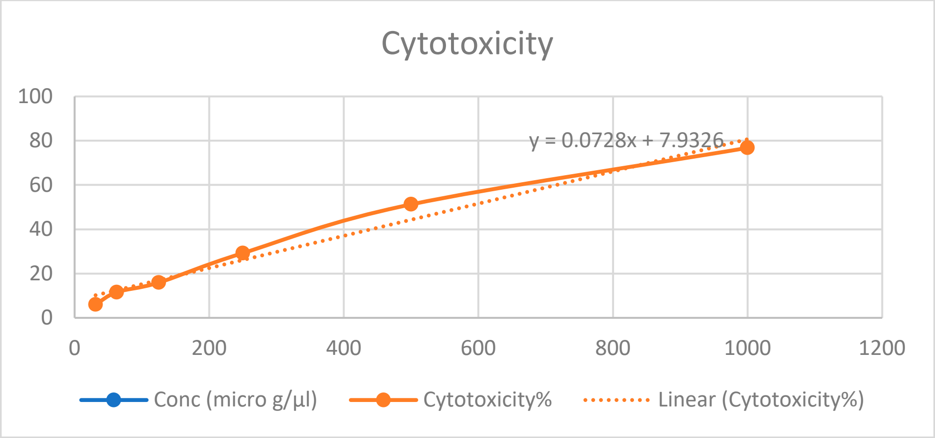

4C	Conc (micro g/µl)	Cytotoxicity%
	1000	79.12321
	500	41.45364
	250	39.12891
	125	28.11989
	62.5	23.12398
	31.2	15.02359

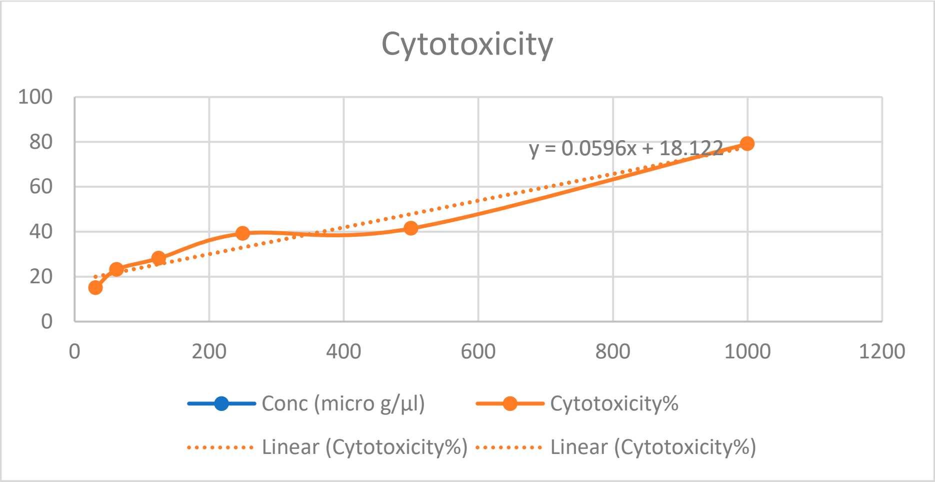

5C	Conc (micro g/µl)	Cytotoxicity%
	1000	85.9899
	500	50.11032
	250	37.12091
	125	28.00918
	62.5	12.97801
	31.2	10.65442

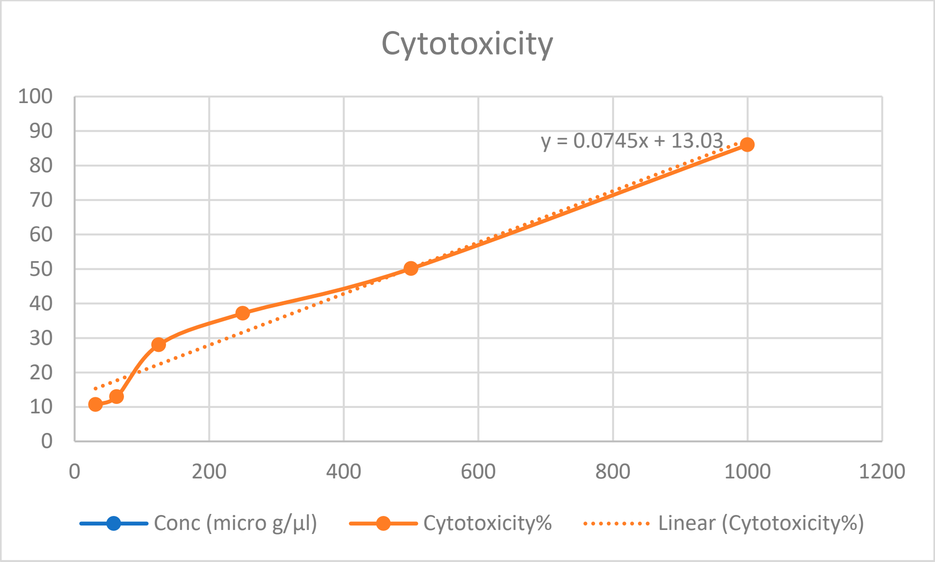

3B	Conc (micro g/µl)	Cytotoxicity%
	1000	83.6251
	500	53.8761
	250	39.1991
	125	22.0918
	62.5	17.1098
	31.2	4.65510

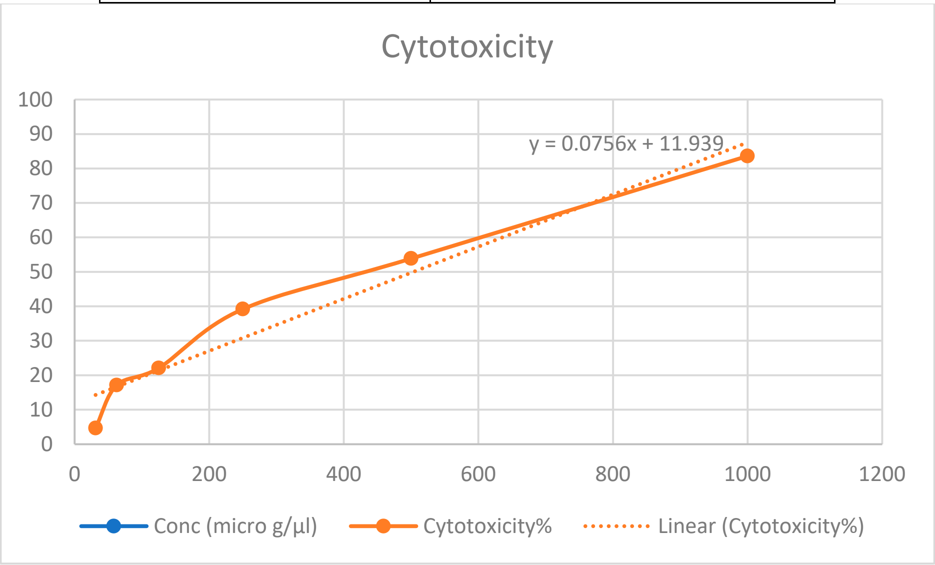

4B	Conc (micro g/µl)	Cytotoxicity%
	1000	77.0009
	500	58.0981
	250	49.1205
	125	37.76181
	62.5	25.1651
	31.2	19.18054

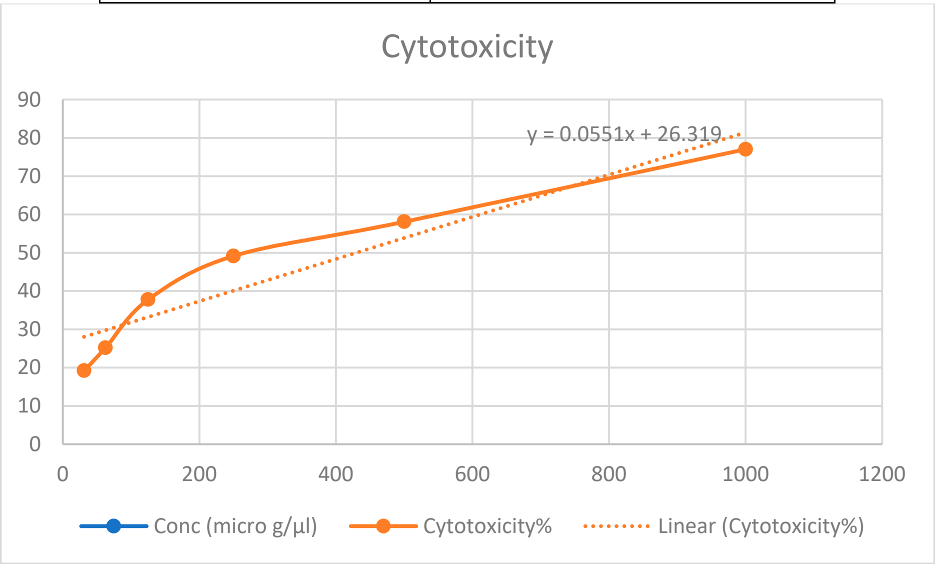

5B	Conc (micro g/µl)	Cytotoxicity%
	1000	90.9826
	500	60.2976
	250	44.2976
	125	30.0092
	62.5	21.14378
	31.2	16.1887

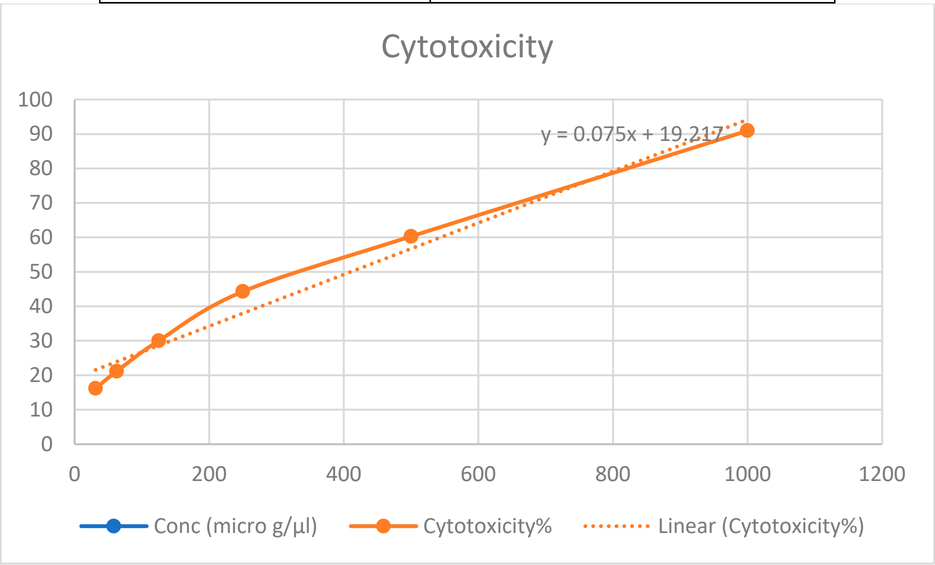

3D	Conc (micro g/µl)	Cytotoxicity%
	1000	89.5618
	500	56.1097
	250	44.2652
	125	32.7651
	62.5	21.1987
	31.2	9.19871

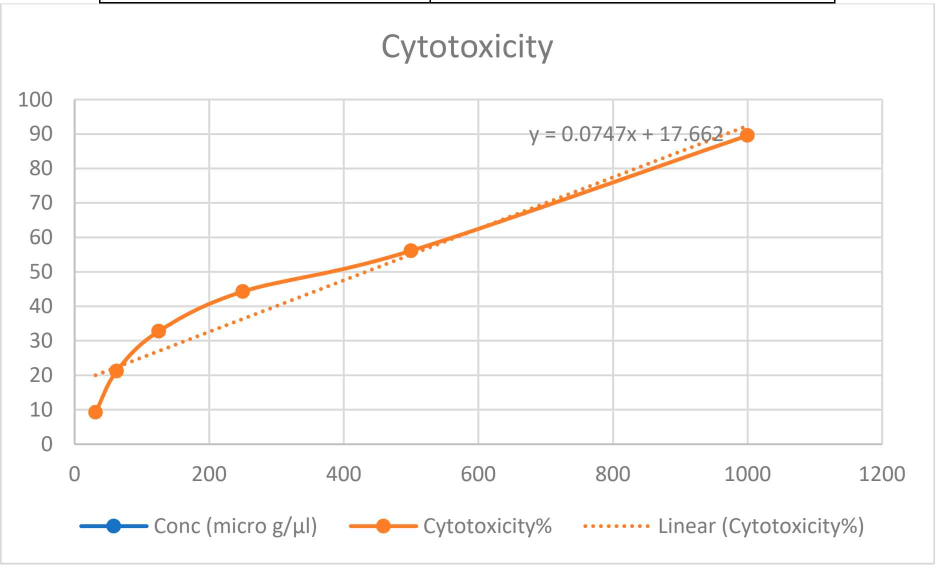

4D	Conc (micro g/µl)	Cytotoxicity%
	1000	88.01981
	500	55.66551
	250	30.19711
	125	23.19871
	62.5	11.18711
	31.2	2.187511

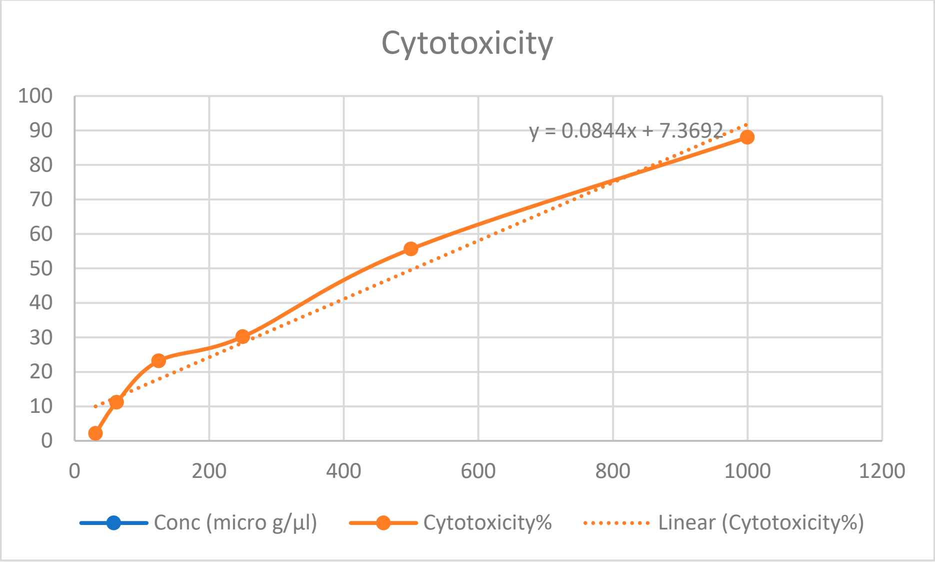

5D	Conc (micro g/µl)	Cytotoxicity%
	1000	73.14226
	500	56.7811
	250	29.0912
	125	18.07912
	62.5	14.7896
	31.2	8.90569

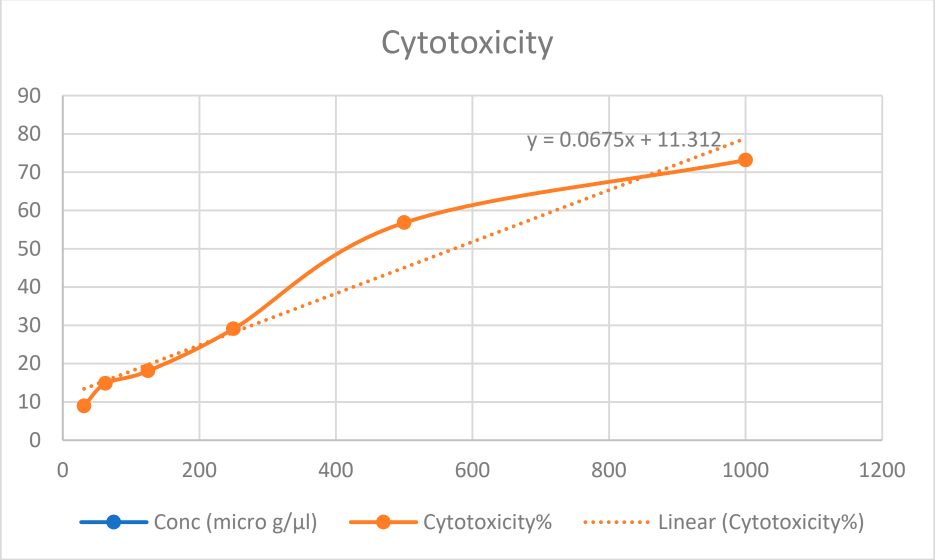

4E	Conc (micro g/µl)	Cytotoxicity%
	1000	77.09444
	500	50.9866
	250	22.45123
	125	14.3221
	62.5	11.3308
	31.2	2.30256

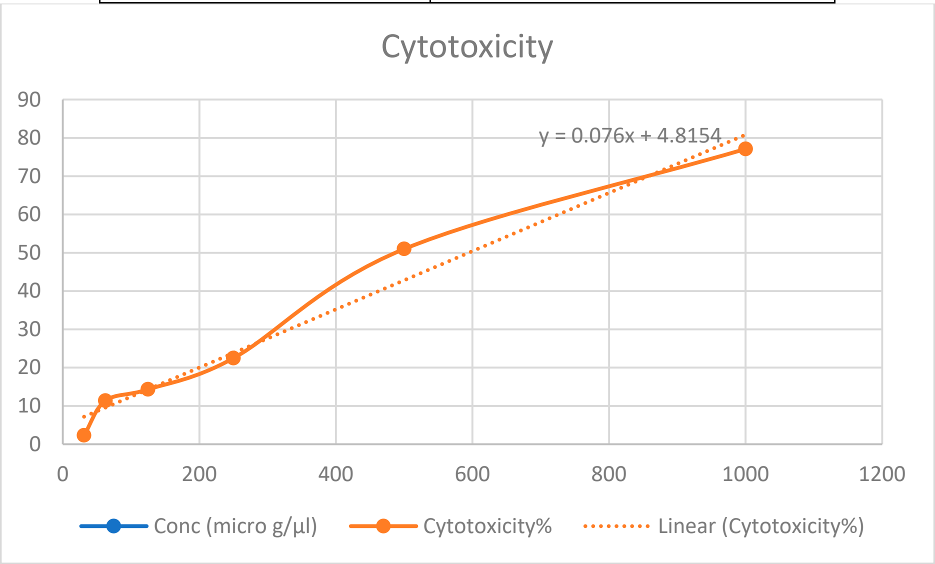

5E	Conc (micro g/µl)	Cytotoxicity%
	1000	88.1425
	500	56.2314
	250	22.2232
	125	11.33456
	62.5	10.98061
	31.2	5.18705

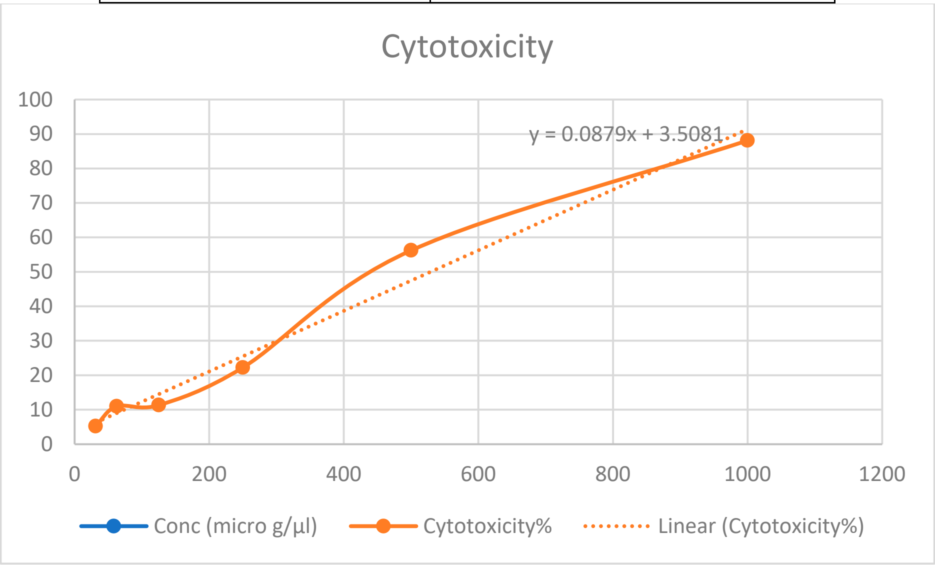

Docking study figures

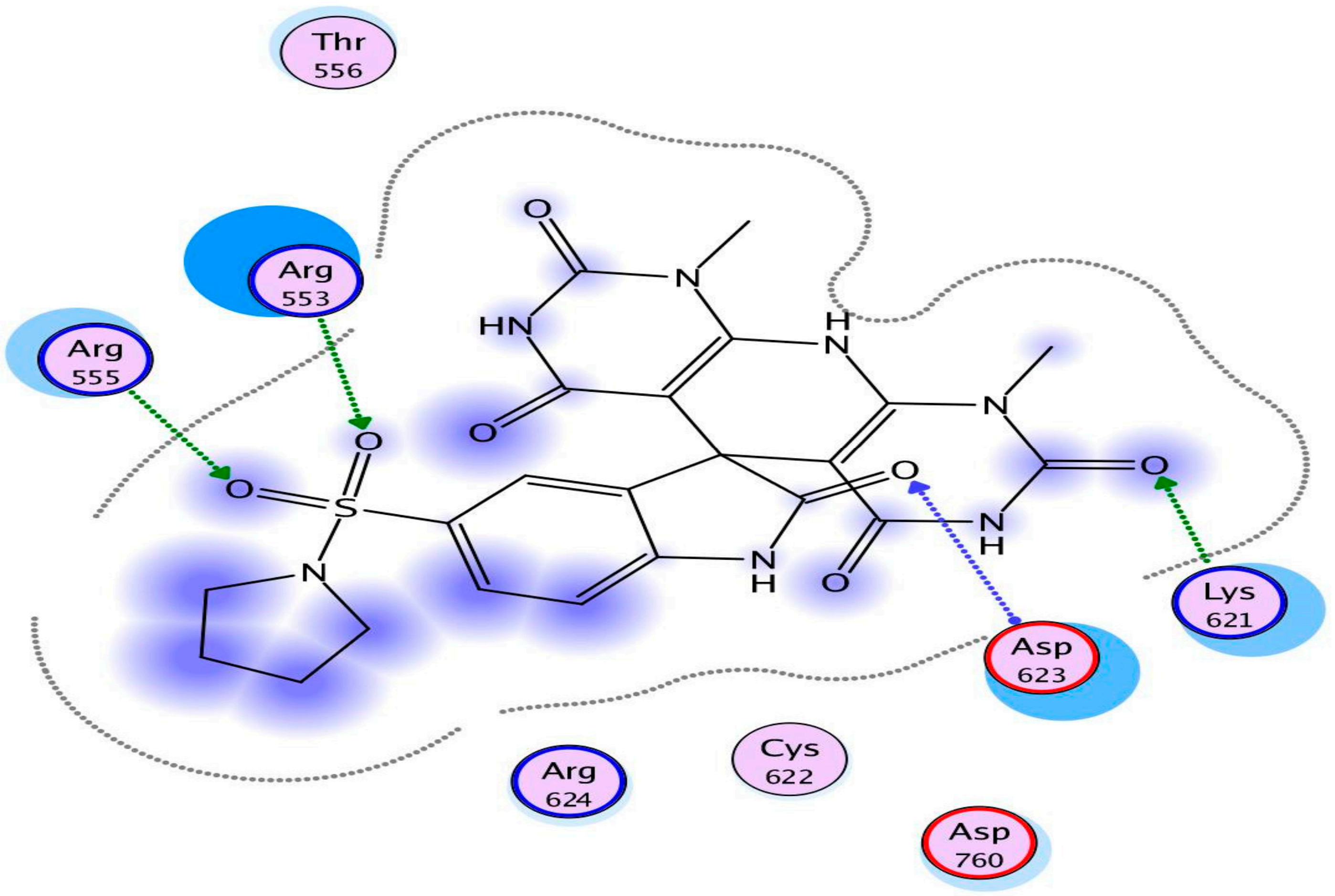

2D structure of compound **3a** inside the active site of RNA polymerase (RdRp) (PDB: 6m71)

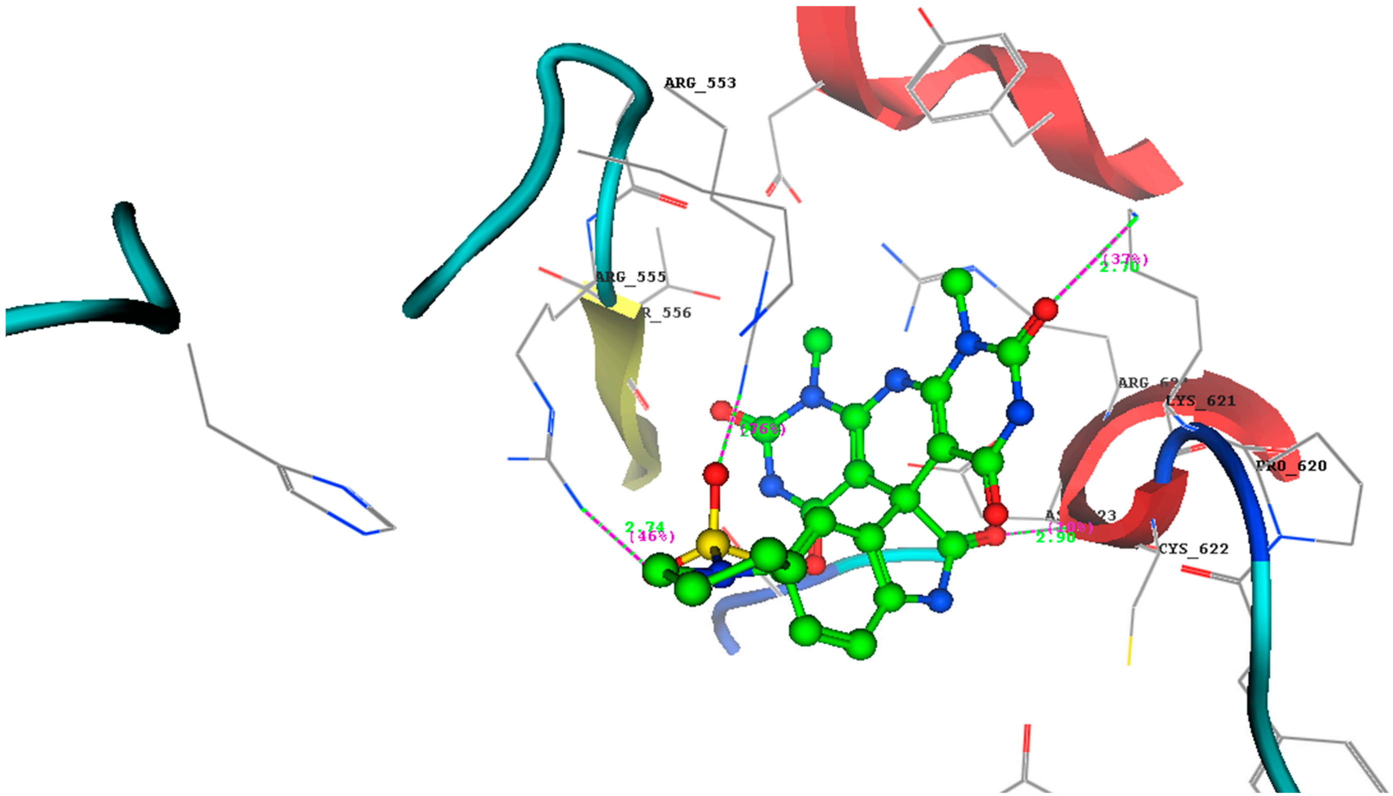

3D structure of compound **3a** inside the active site of RNA polymerase (RdRp) (PDB: 6m71)

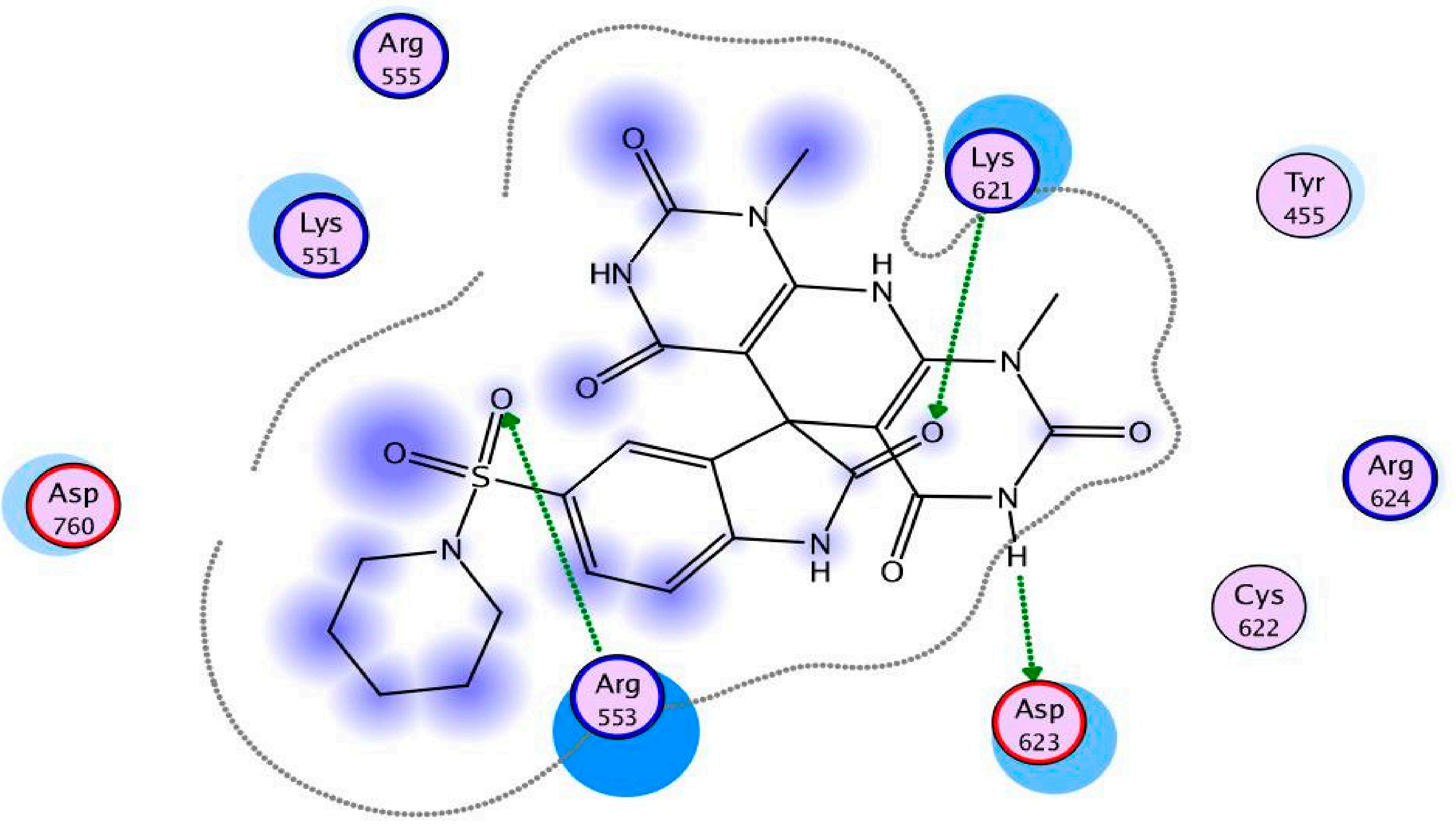

2D structure of compound **4b** inside the active site of RNA polymerase (RdRp) (PDB: 6m71)

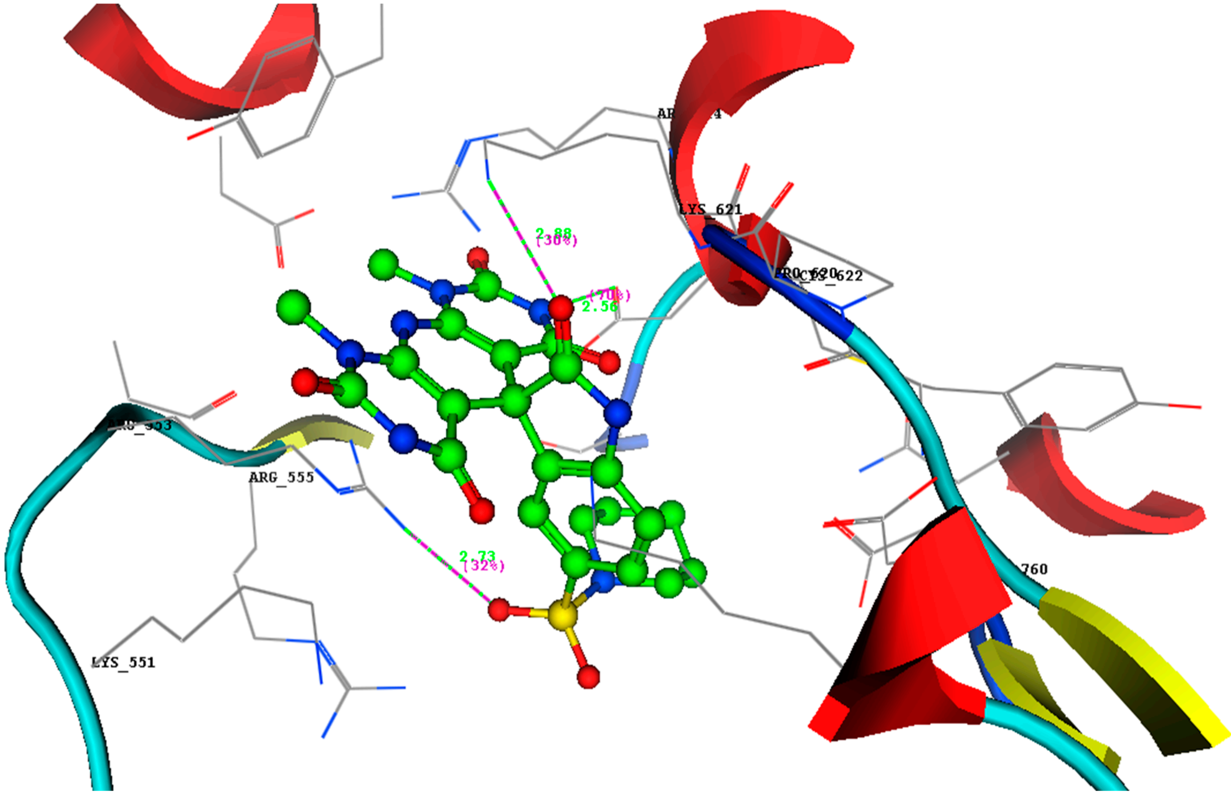

3D structure of compound **4b** inside the active site of RNA polymerase (RdRp) (PDB: 6m71)

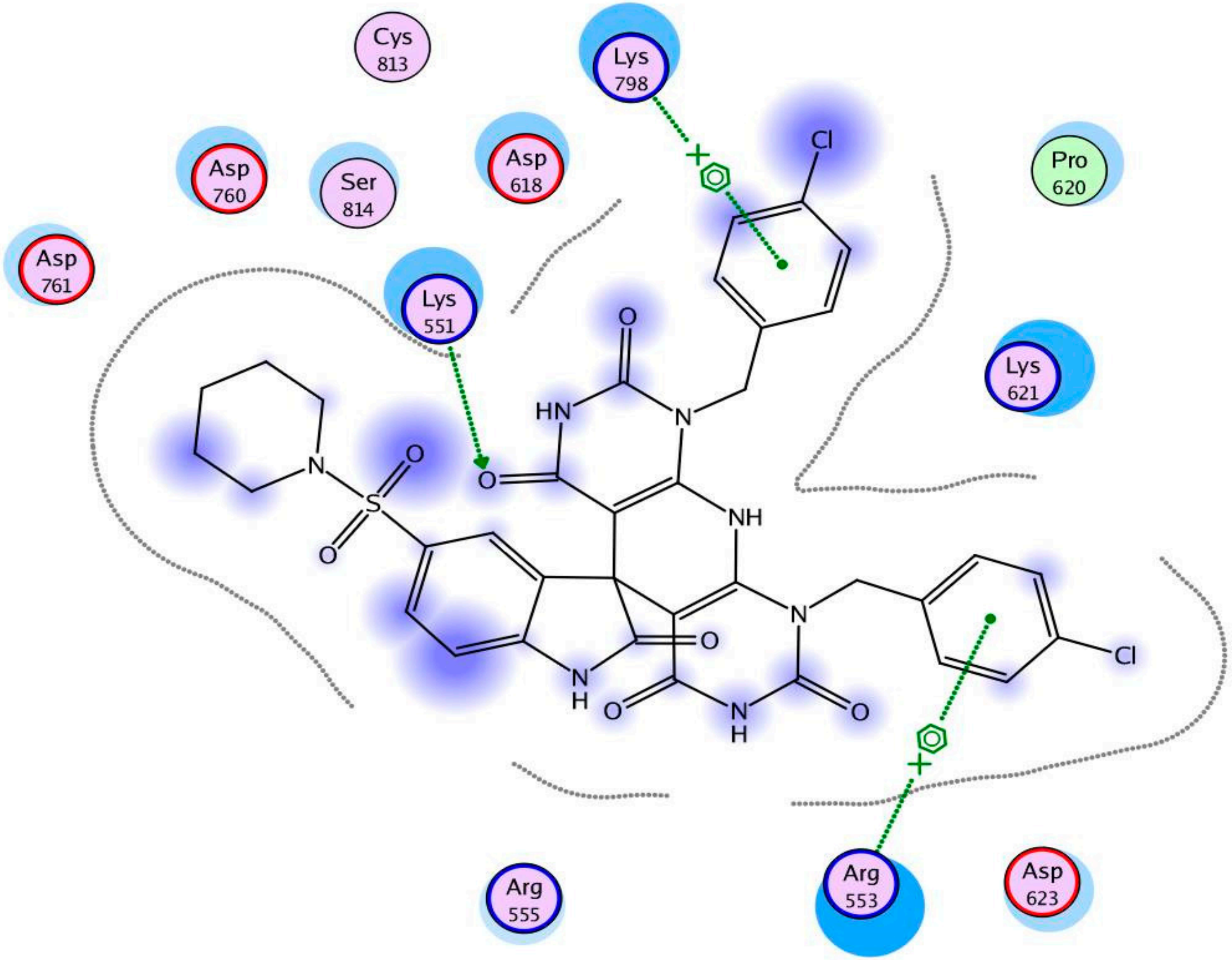

2D structure of compound **4d** inside the active site of RNA polymerase (RdRp) (PDB: 6m71)

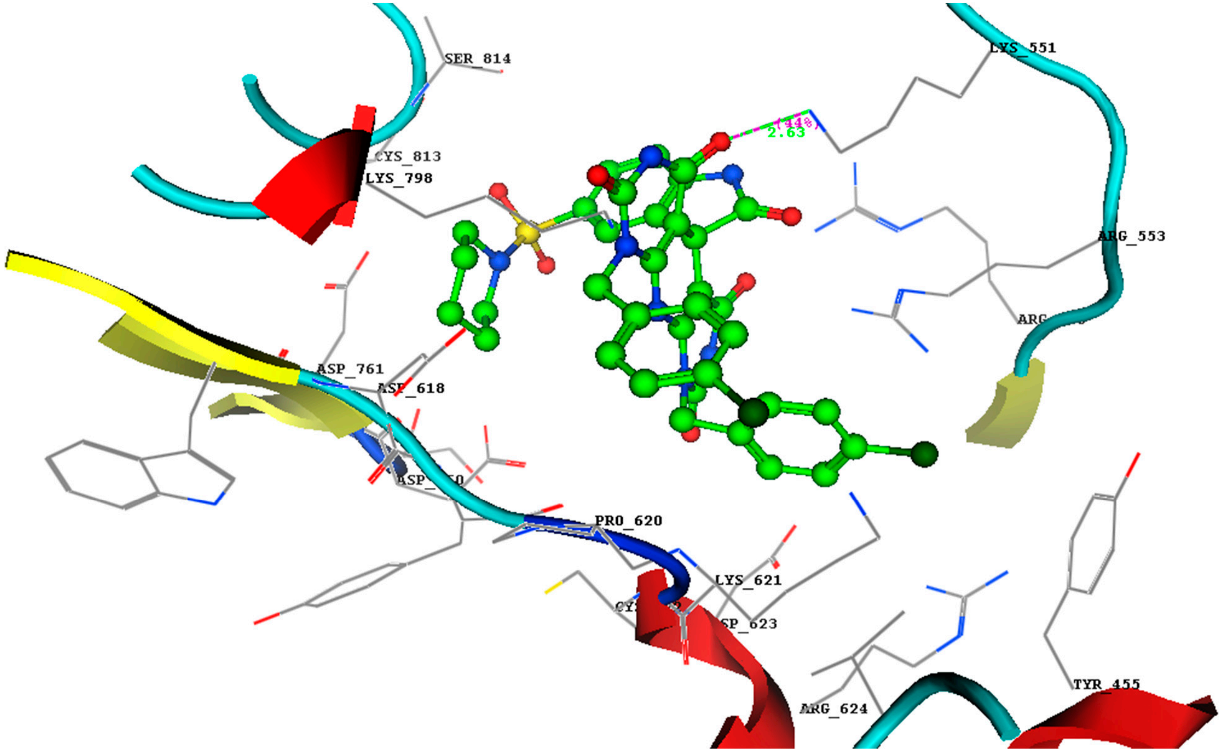

3D structure of compound **4d** inside the active site of RNA polymerase (RdRp) (PDB: 6m71)

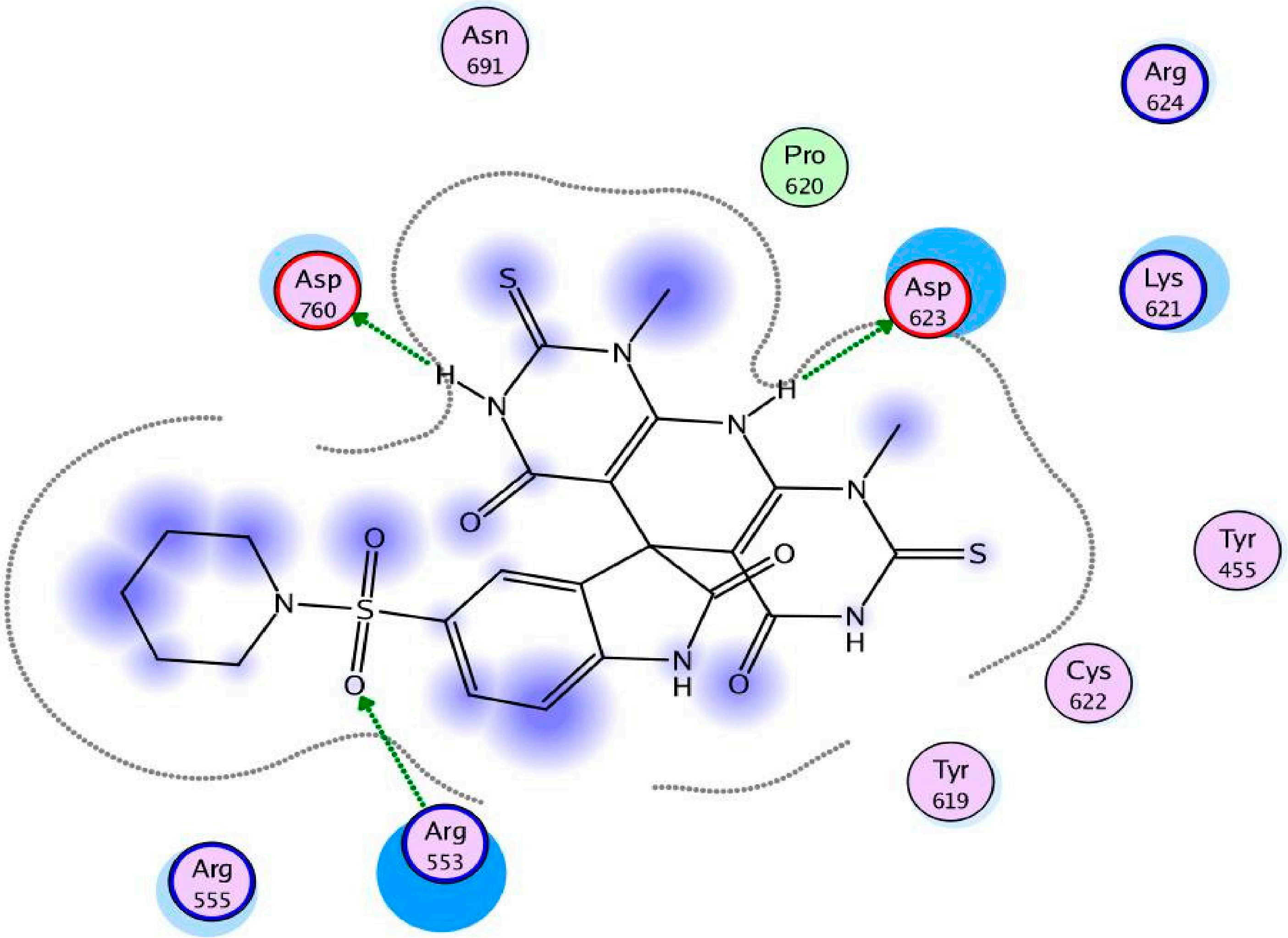

2D structure of compound **4e** inside the active site of RNA polymerase (RdRp) (PDB: 6m71)

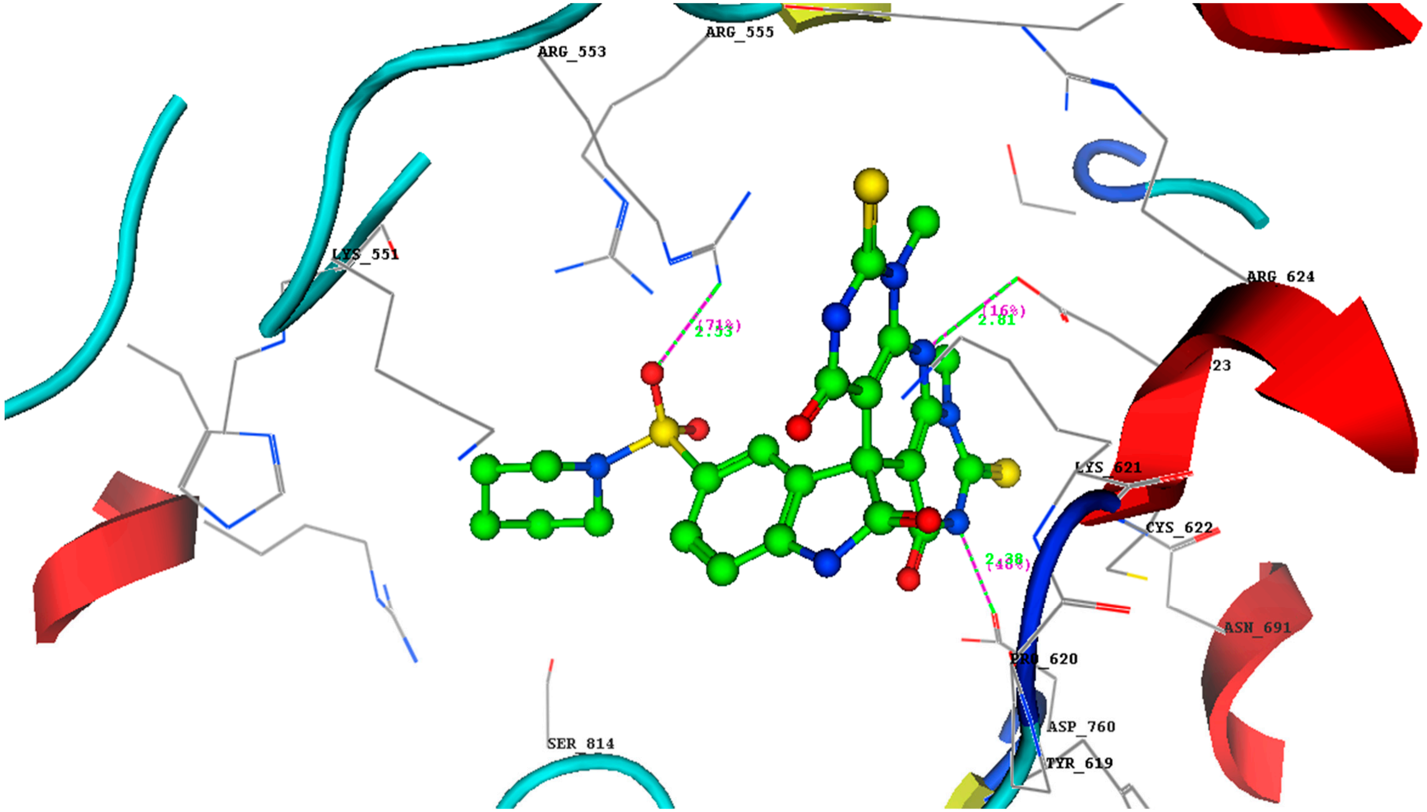

3D structure of compound **4e** inside the active site of RNA polymerase (RdRp) (PDB: 6m71)

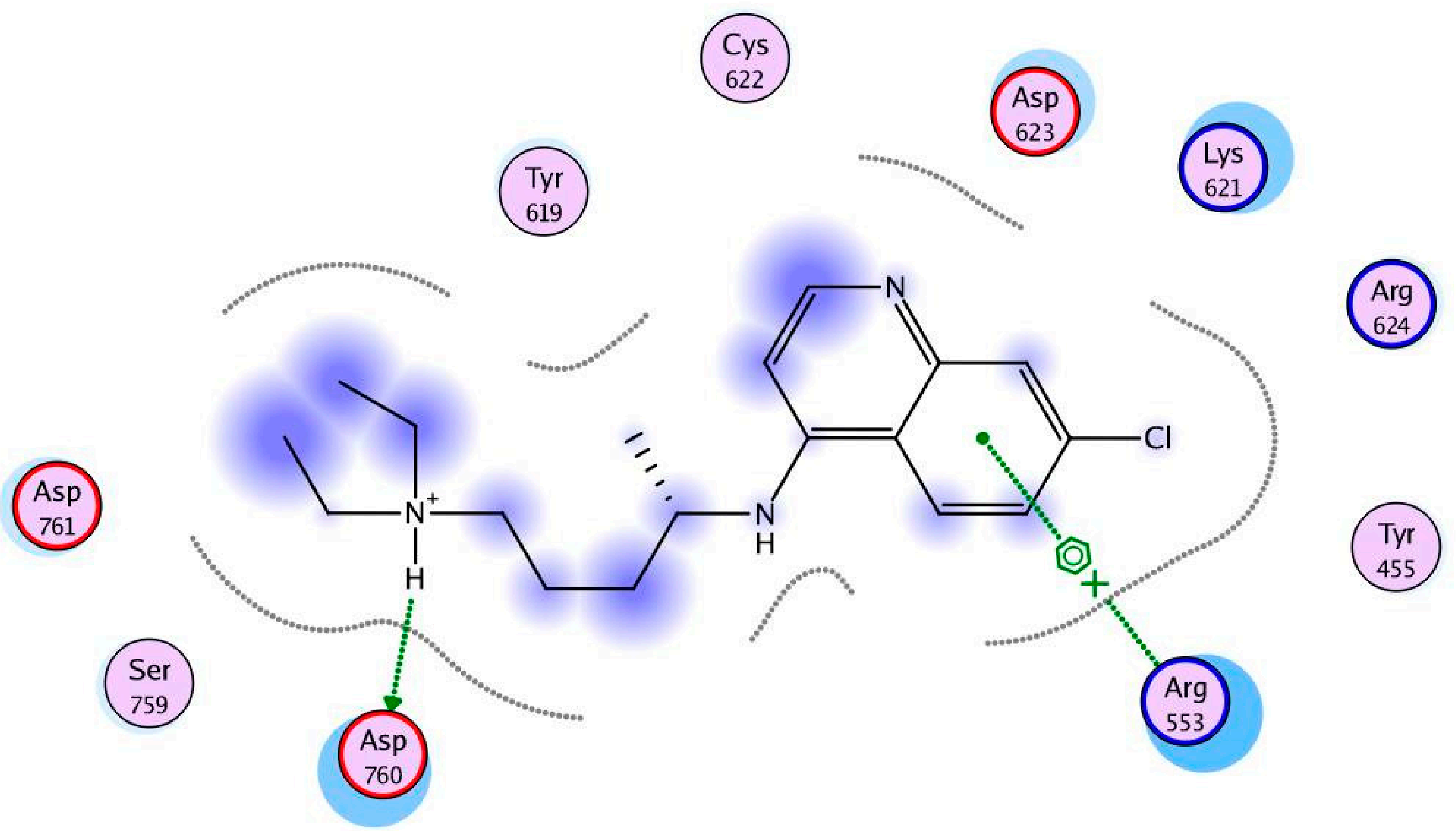

2D structure of **Chloroquine** inside the active site of RNA polymerase (RdRp) (PDB: 6m71)

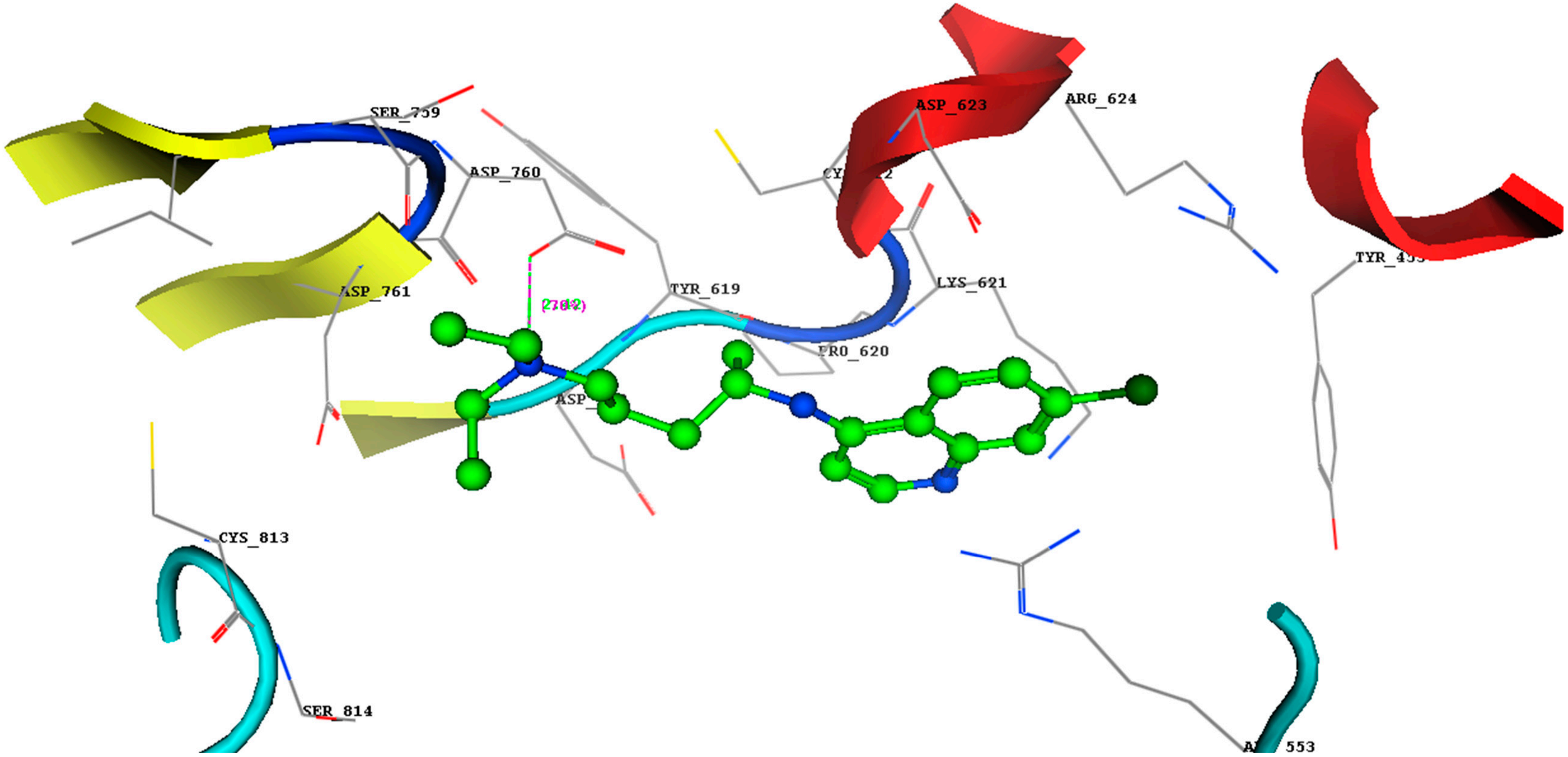

3D structure of **Chloroquine** inside the active site of RNA polymerase (RdRp) (PDB: 6m71)

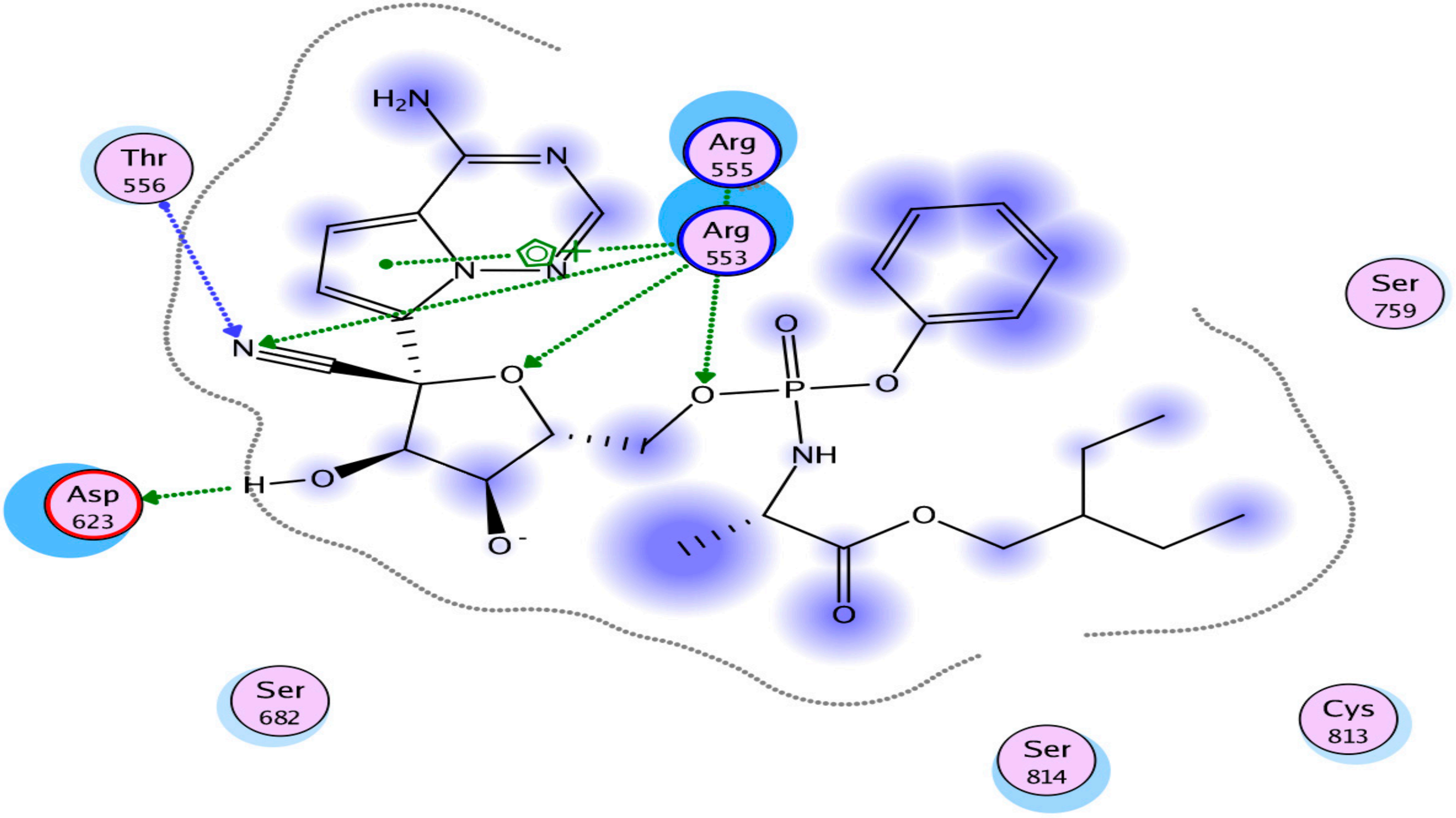

2D structure of **Remdesivir** inside the active site of RNA polymerase (RdRp) (PDB: 6m71)

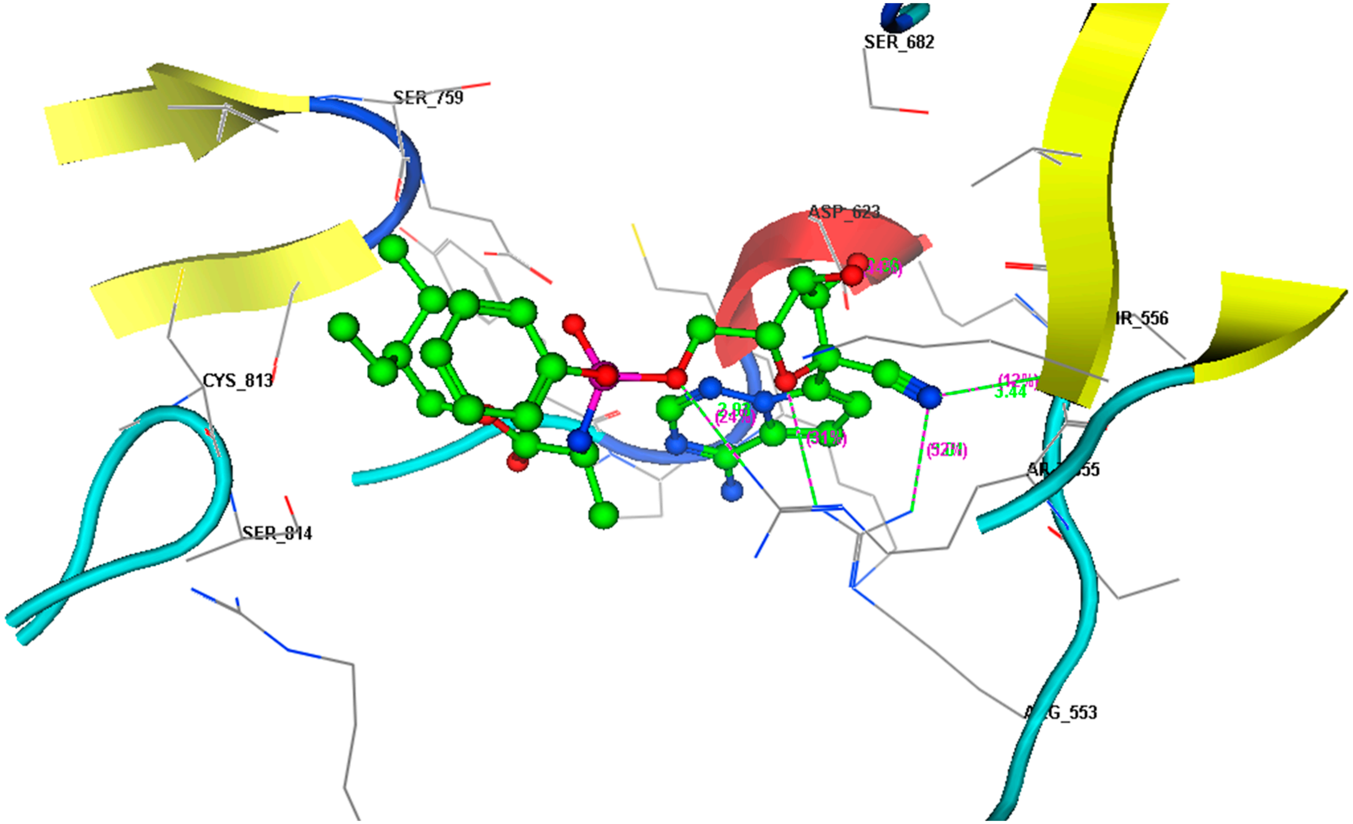

3D structure of **Remdesivir** inside the active site of RNA polymerase (RdRp) (PDB: 6m71)

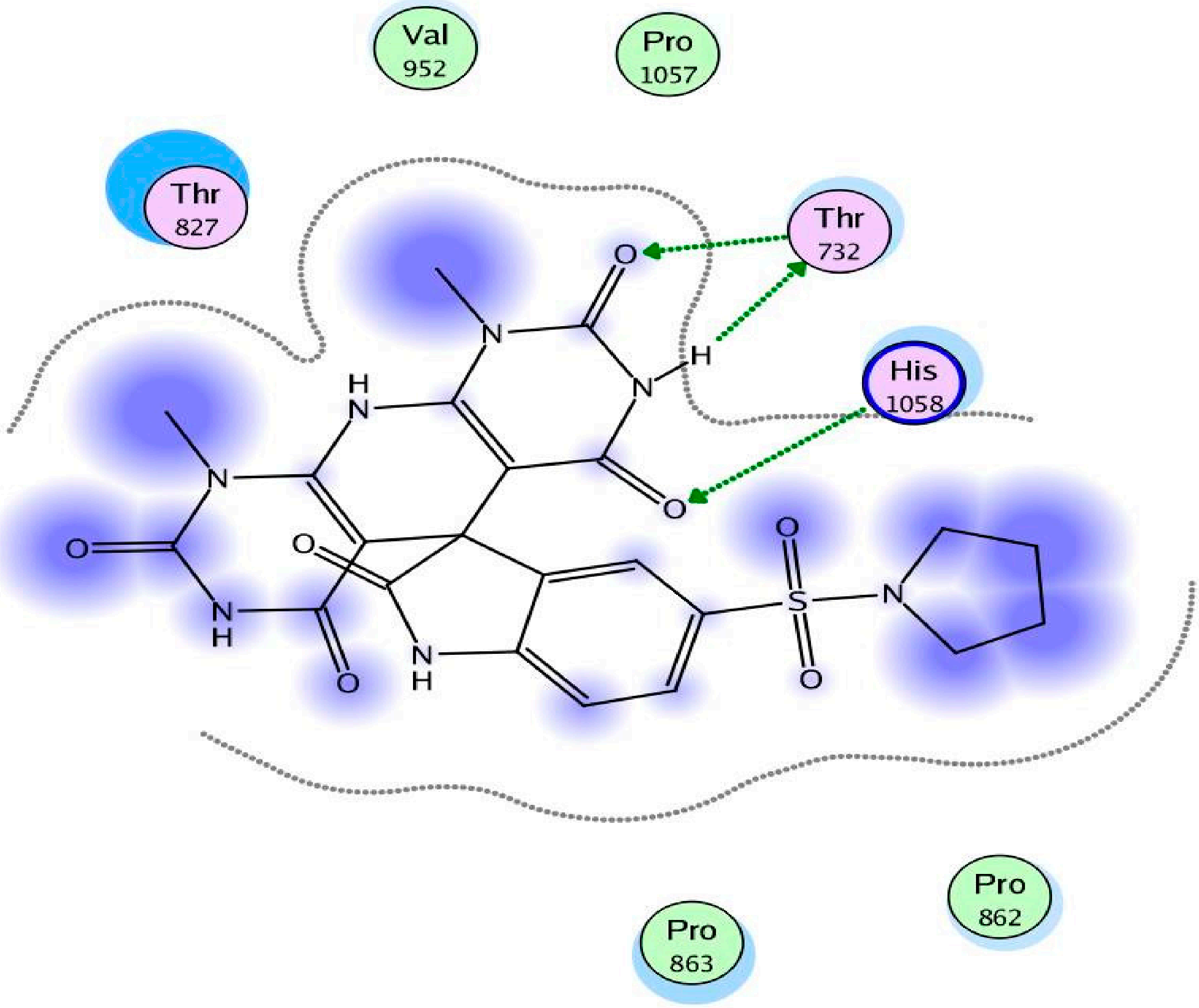

2D structure of compound **3a** inside the active site of spike glycoprotein (SGp) (PDB: 6VXX)

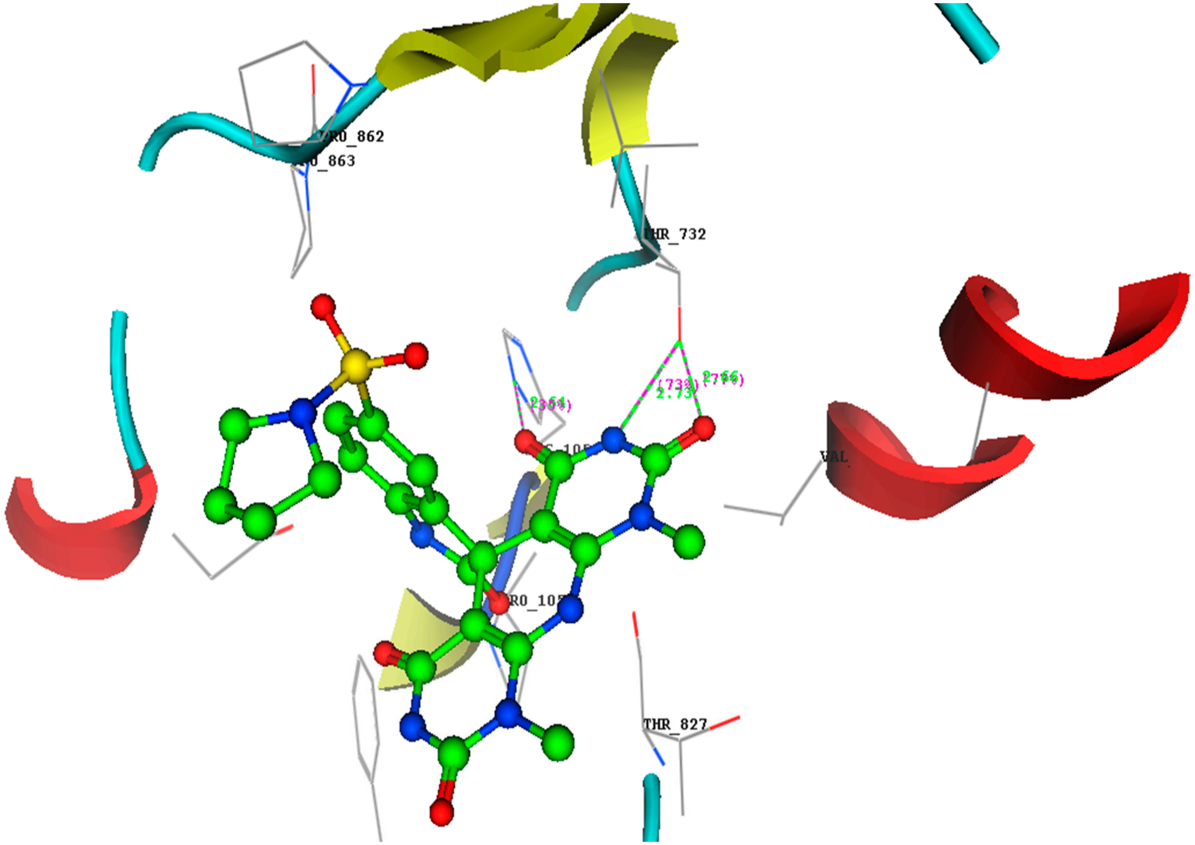

3D structure of compound **3a** inside the active site of spike glycoprotein (SGp) (PDB: 6VXX)

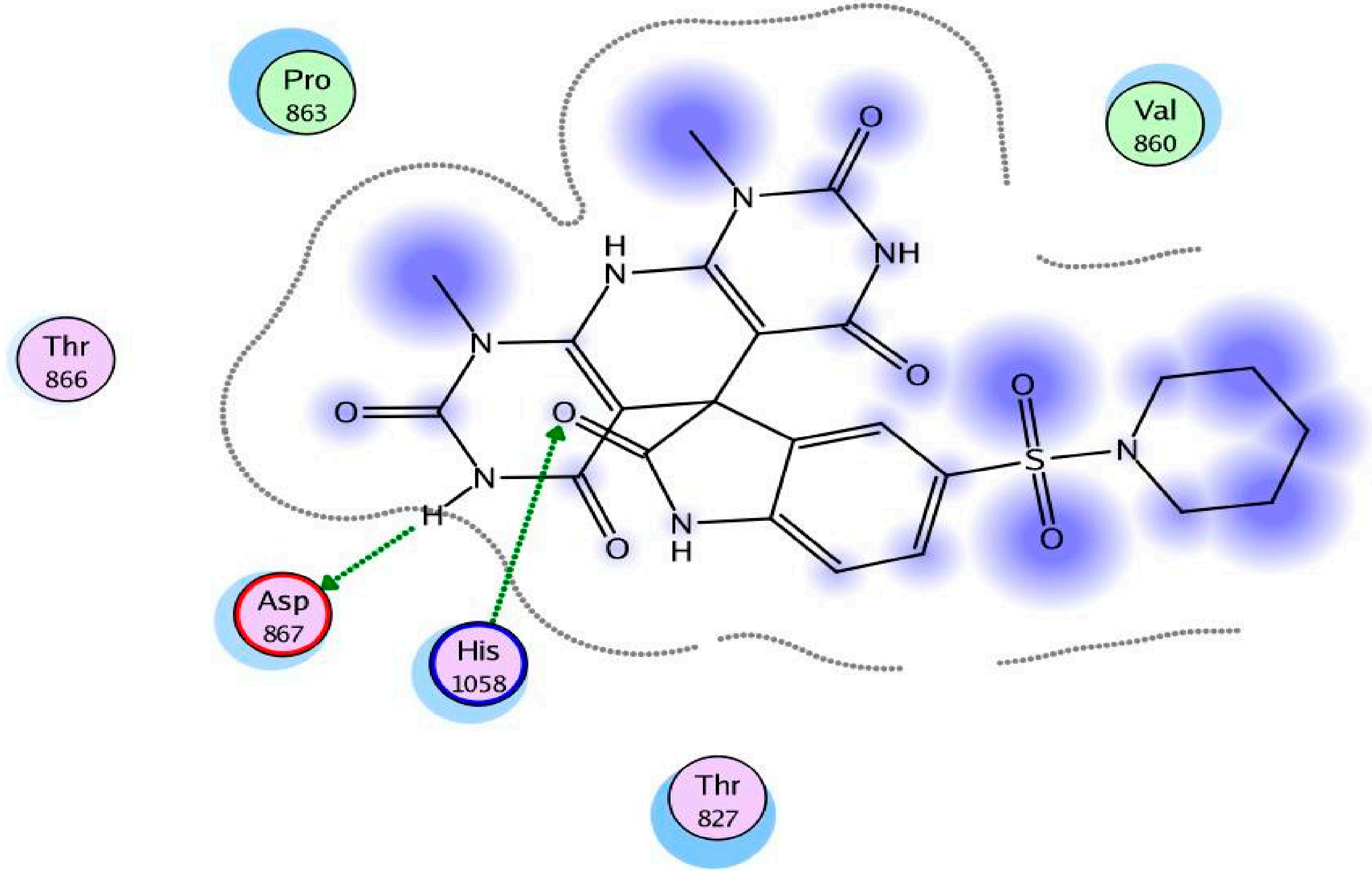

2D structure of compound **4b** inside the active site of spike glycoprotein (SGp) (PDB: 6VXX)

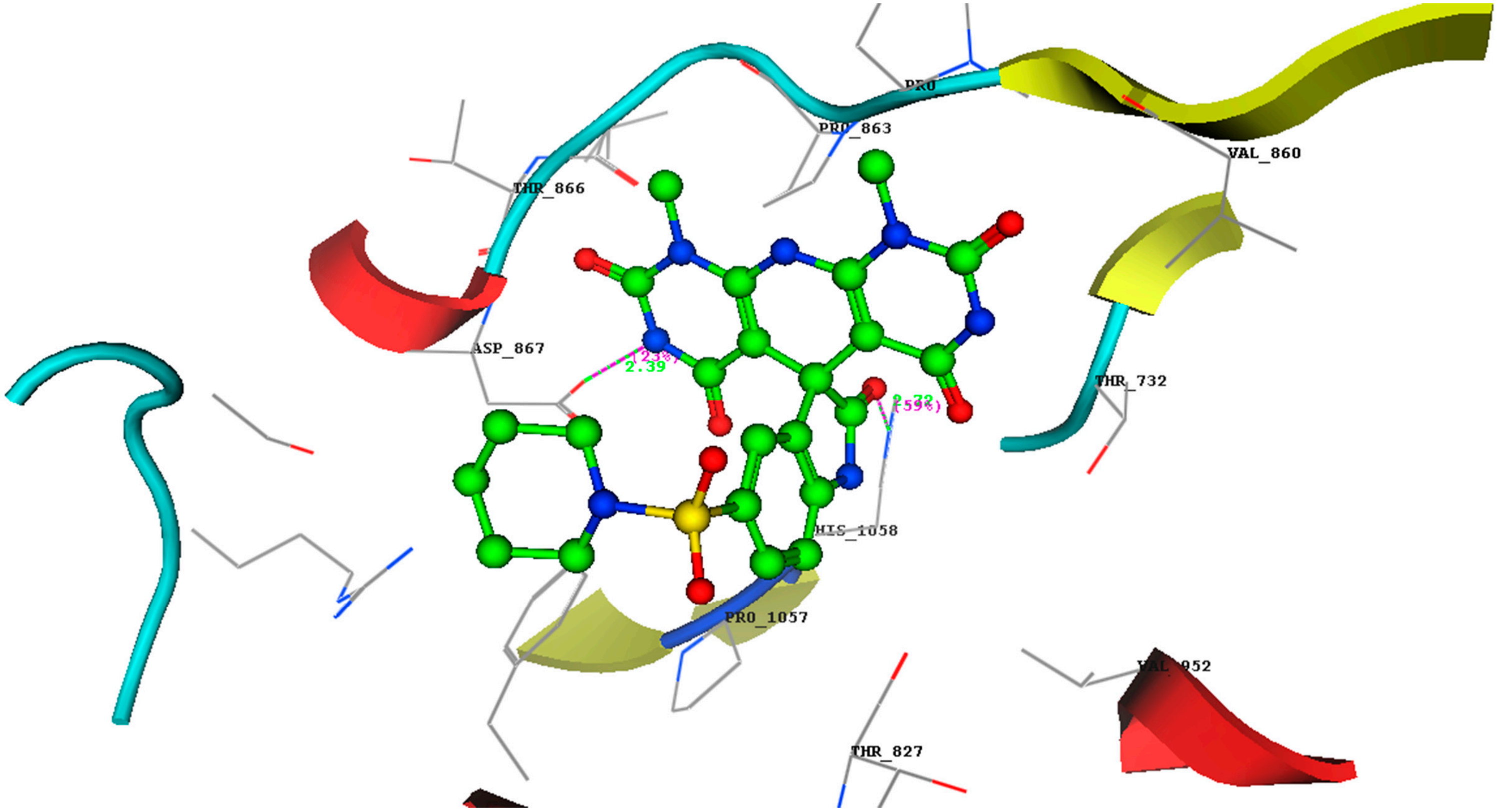

3D structure of compound **4b** inside the active site of spike glycoprotein (SGp) (PDB: 6VXX)

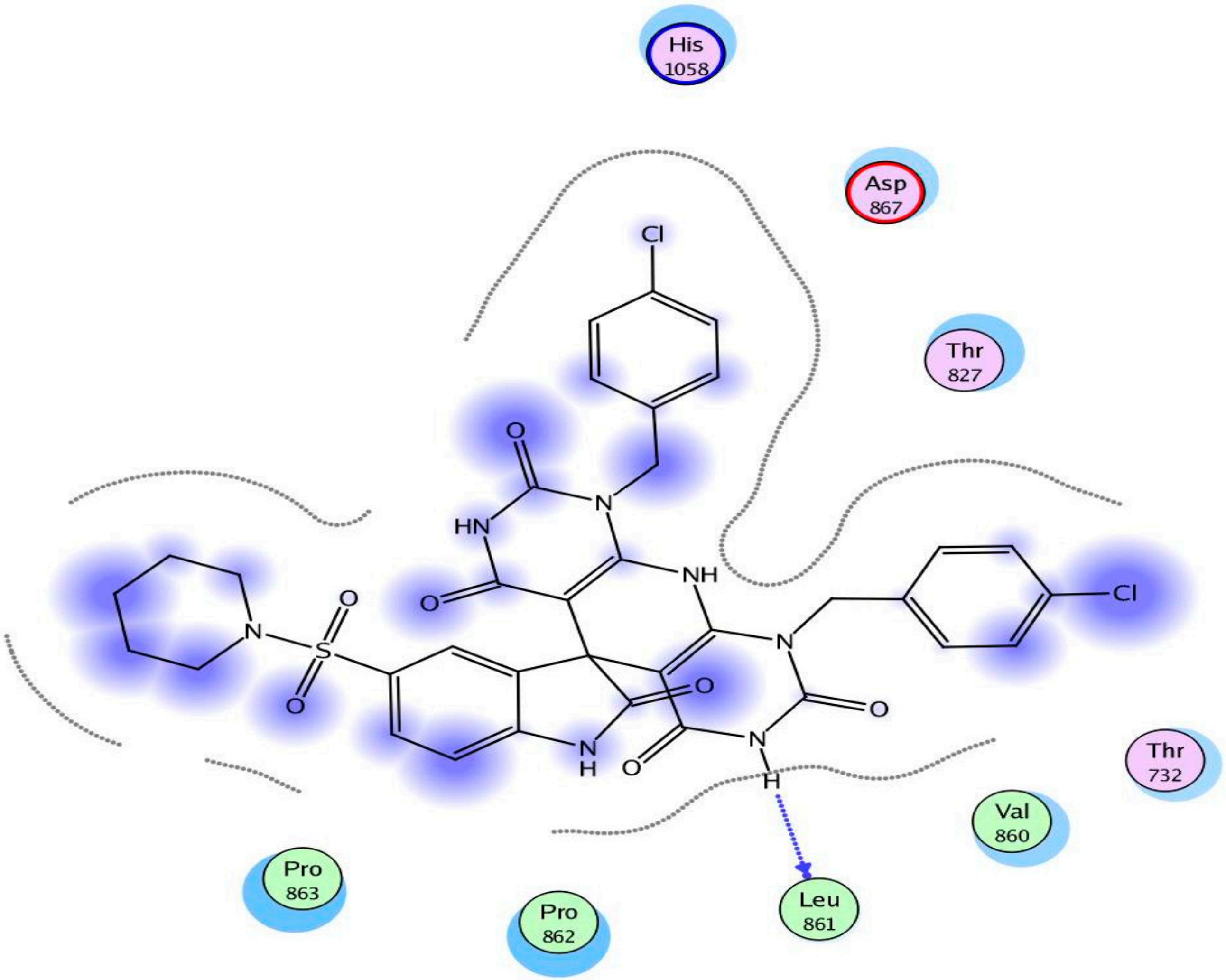

2D structure of compound **4d** inside the active site of spike glycoprotein (SGp) (PDB: 6VXX)

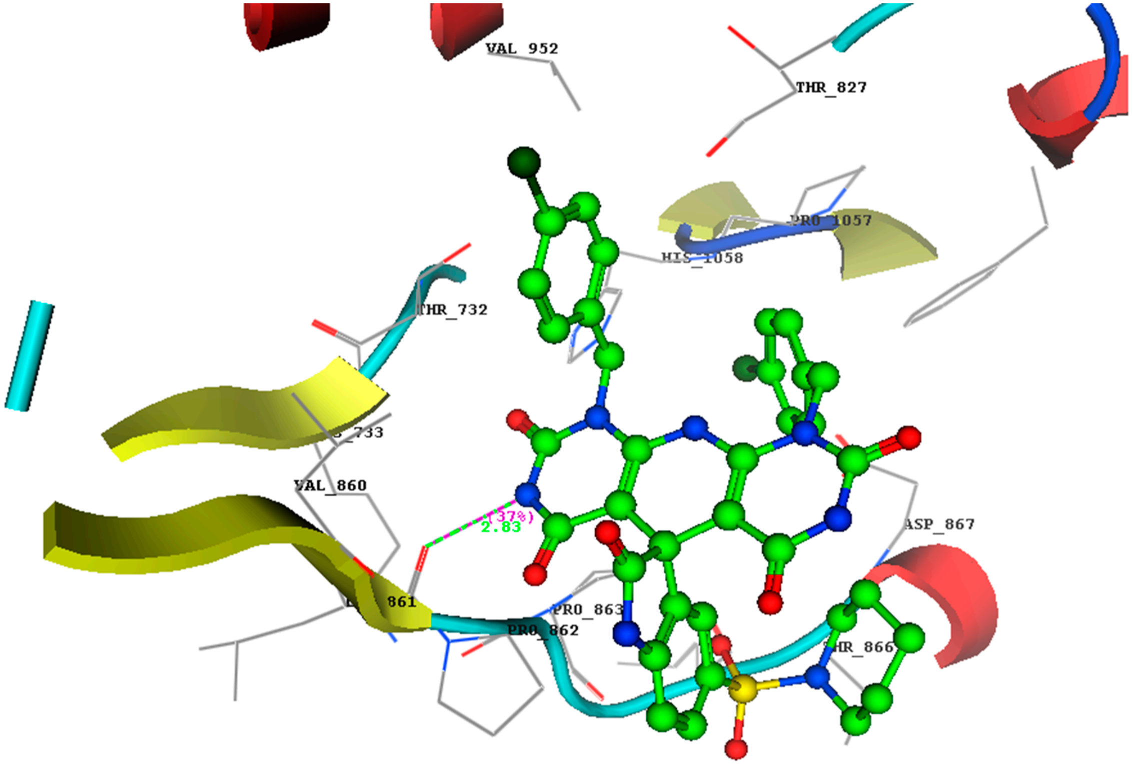

3D structure of compound **4d** inside the active site of spike glycoprotein (SGp) (PDB: 6VXX)

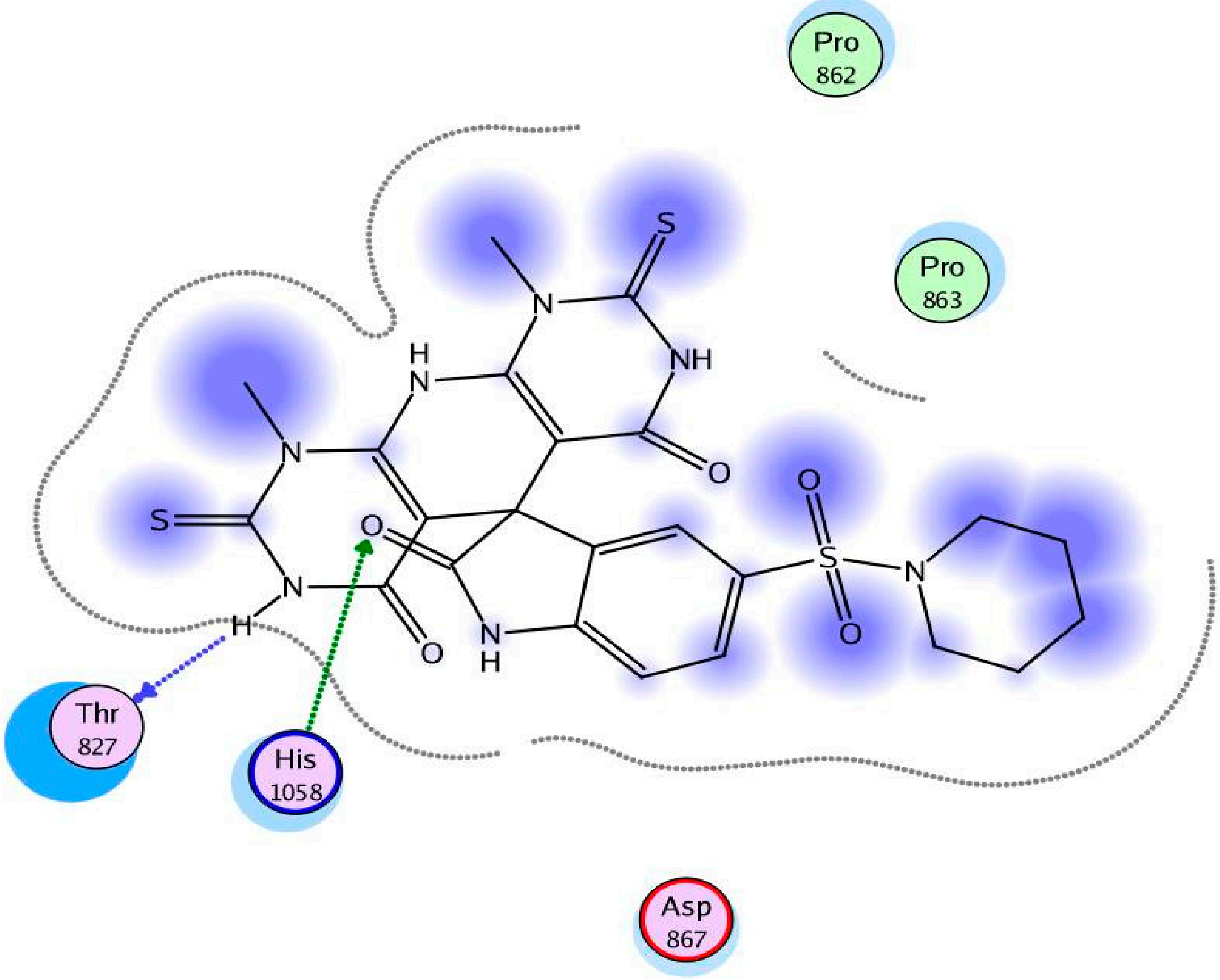

2D structure of compound **4e** inside the active site of spike glycoprotein (SGp) (PDB: 6VXX)

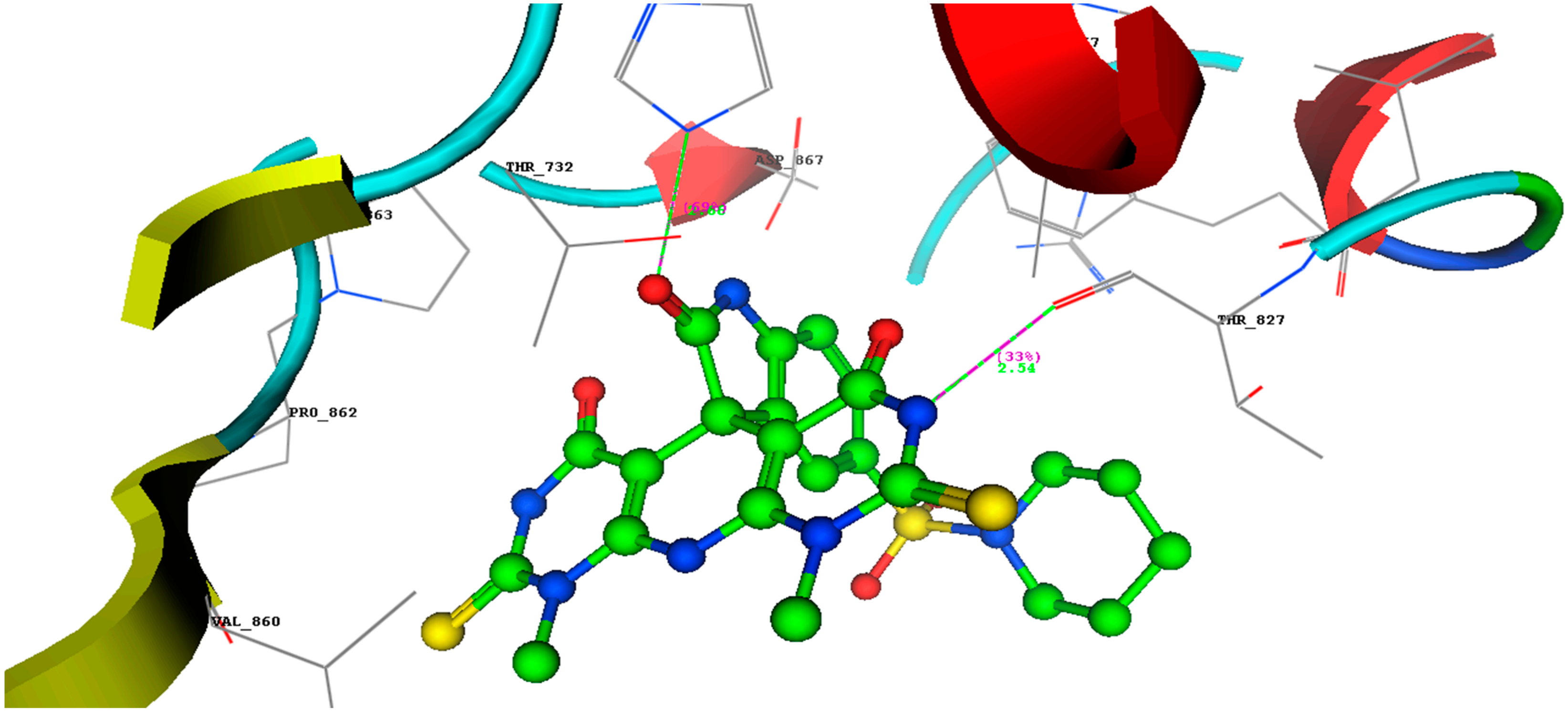

3D structure of compound **4e** inside the active site of spike glycoprotein (SGp) (PDB: 6VXX)

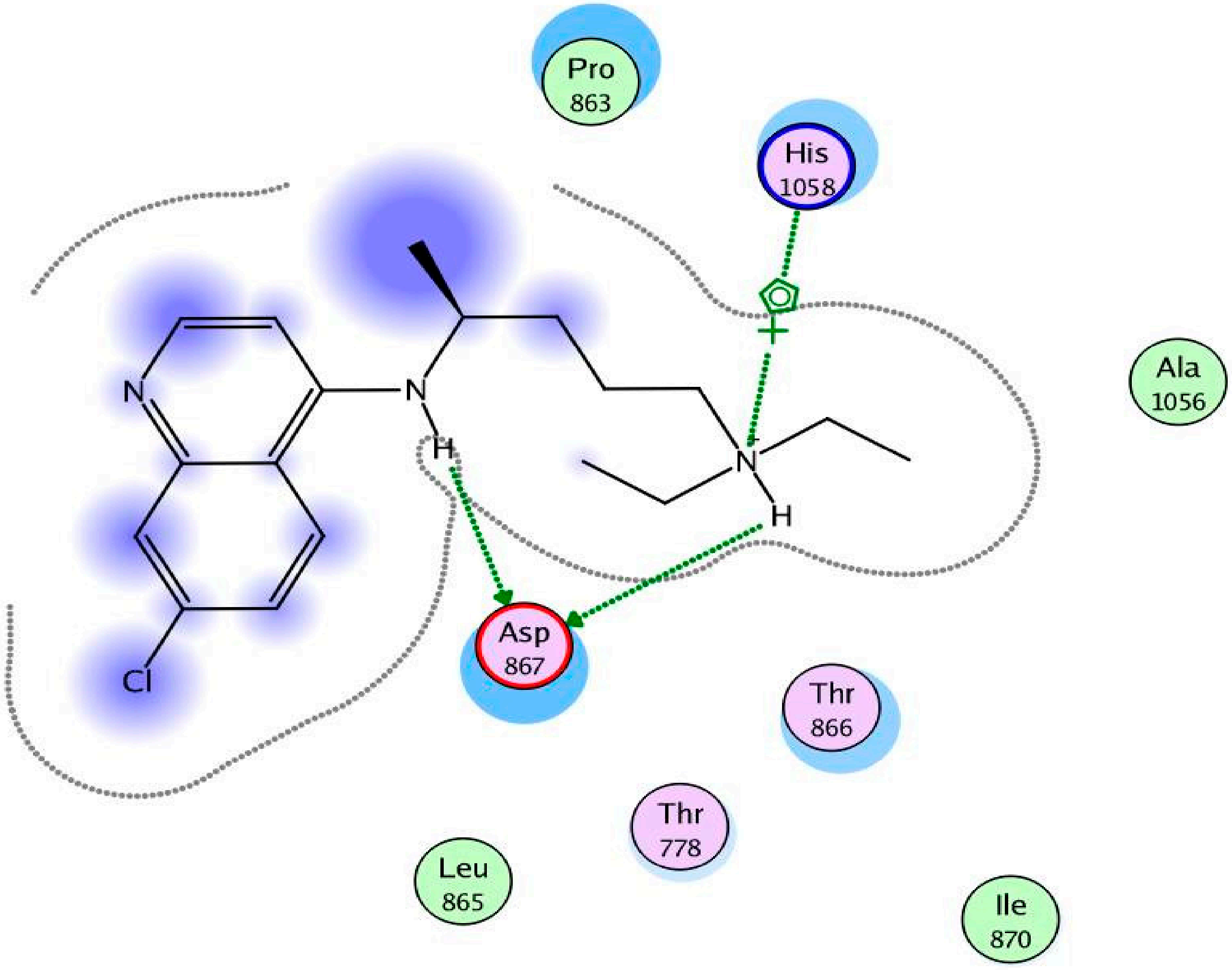

2D structure of **Chloroquine** inside the active site of spike glycoprotein (SGp) (PDB: 6VXX)

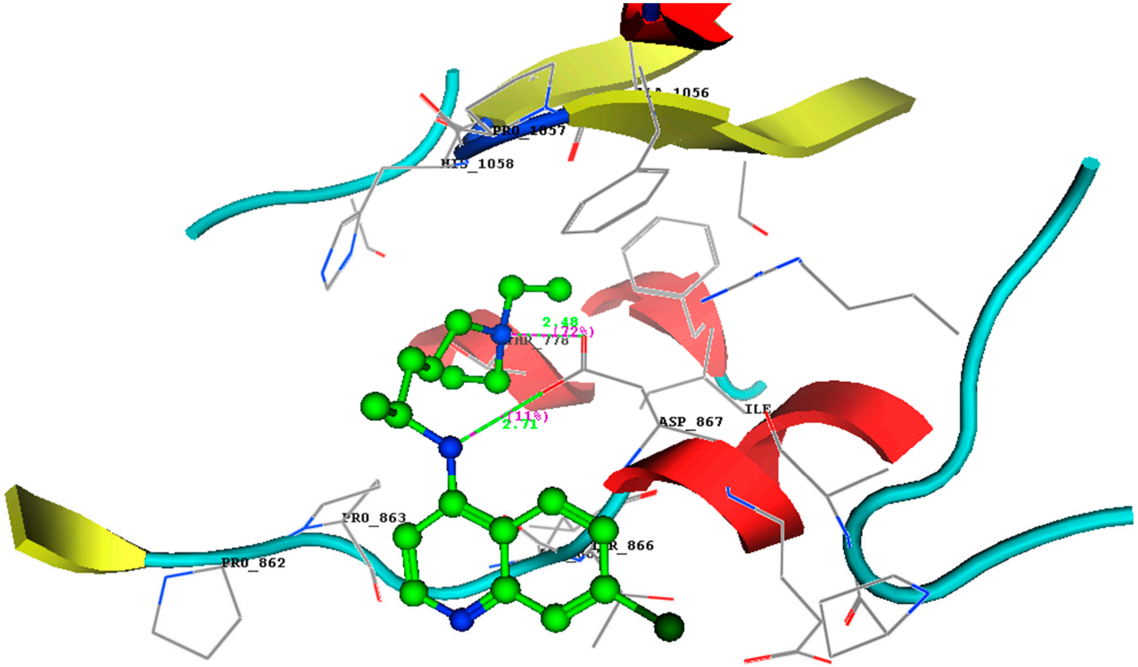

3D structure of **Chloroquine** inside the active site of spike glycoprotein (SGp) (PDB: 6VXX)

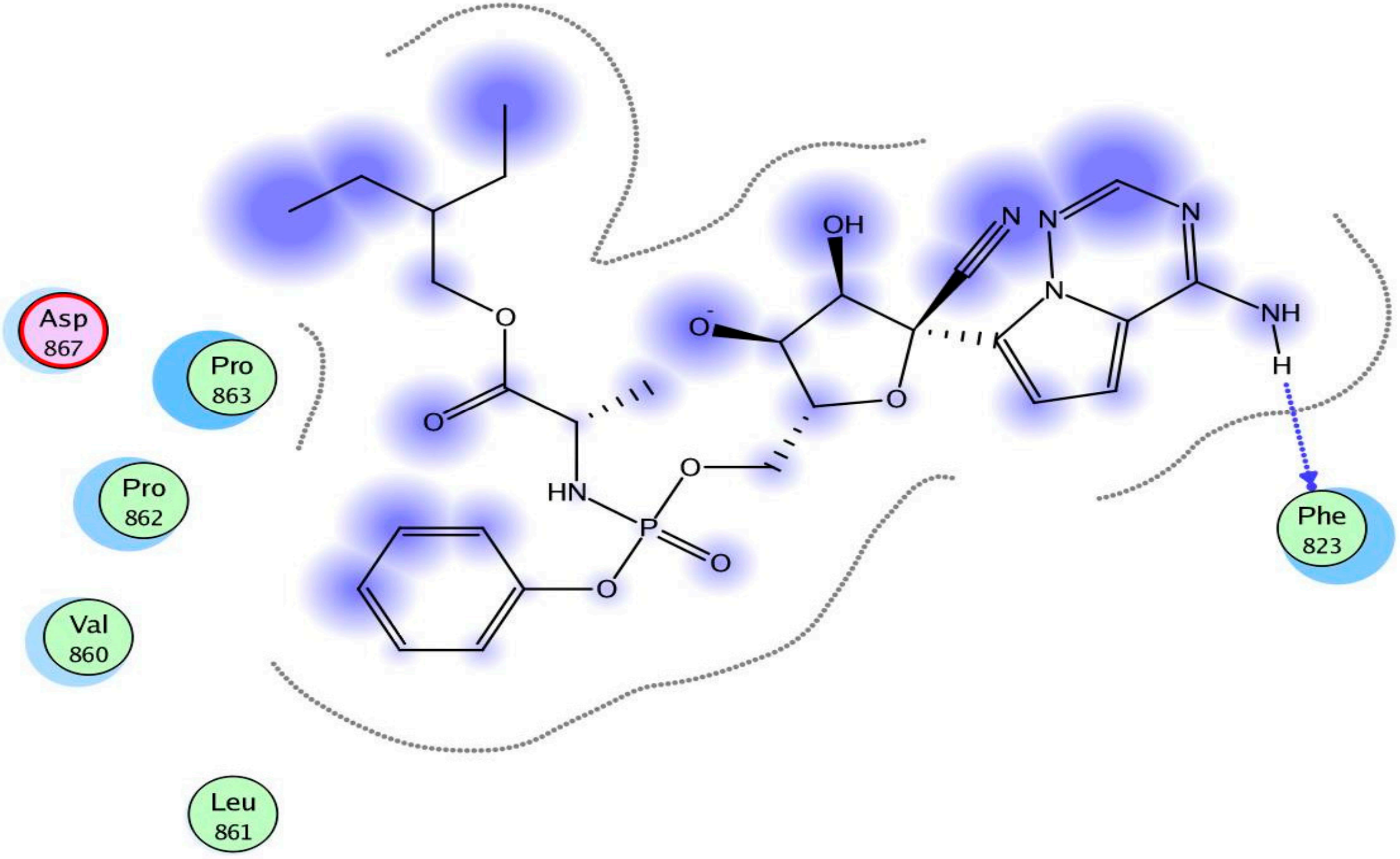

2D structure of **Remdesivir** inside the active site of spike glycoprotein (SGp) (PDB: 6VXX)

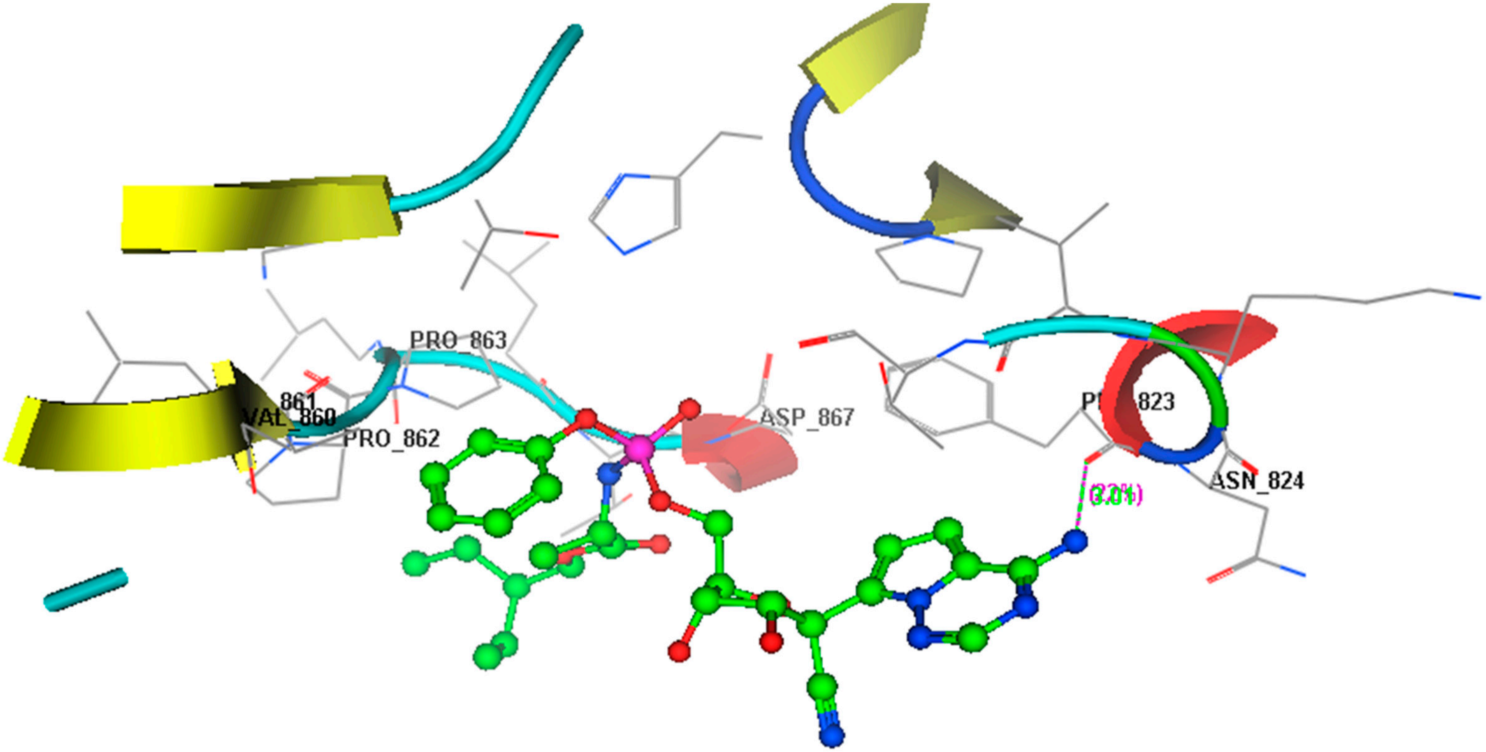

3D structure of **Remdesivir** inside the active site of spike glycoprotein (SGp) (PDB: 6VXX)
